# Temperature-Controlled Divergent Synthesis of Pyrazoles and 1-Tosyl-1*H*-pyrazoles under Transition-Metal-Catalyst- and Oxidant-Free Conditions

**DOI:** 10.3390/molecules29081706

**Published:** 2024-04-10

**Authors:** Kai Wang, Wenjing Xu, Chengcai Xia, Xianting Cao

**Affiliations:** 1 College of Medical Engineering & The Key Laboratory for Medical Functional Nanomaterials, Jining Medical University, Jining 272067, China; wangkaichem@mail.jnmc.edu.cn (K.W.); wenjing1230511@163.com (W.X.); 2 Institute of Pharmacology, Pharmacy College, Shandong First Medical University & Shandong Academy of Medical Sciences, Jinan 271016, China

**Keywords:** temperature-controlled, ionic liquids, divergent synthesis, pyrazole derivatives, green strategies

## Abstract

Herein, a general and practical temperature-controlled approach for the divergent synthesis of pyrazoles and 1-tosyl-1*H*-pyrazoles via electrophilic cyclization in the absence of transition-metal catalysts and oxidants was developed. The desired products were obtained in moderate to excellent yields from common starting materials in both ionic liquids and ethanol by simply tuning the reaction temperature. This strategy employs easily synthesized substrates, mild reaction conditions, and excellent functional-group tolerance.

## 1. Introduction

As an impressive nitrogen-containing heterocyclic compound, pyrazole and its derivatives have been widely studied owing to their diverse and potent biological activities, including analgesic [1,2,3], antibacterial [4], antidepressant [5], anti-inflammatory [6], antihypertensive [7], appetite suppressant [8], antihyperglycemic [9], and anti-cancer activities [10]. Thus, numerous studies have been conducted on the synthesis of pyrazoles. Traditional methods involve the reaction of hydrazines and *β*-di-functional compounds such as 1,3-dicarbonyl compounds [11,12,13,14,15,16] or the intermolecular cyclization reaction of diazoalkanes and nitrilimines with unsaturated hydrocarbons. However, the poor regioselectivity greatly limits the application scope of these methods.

Advances in the development of electrophilic cyclization strategies over the past few decades have led to many studies on the synthesis of substituted pyrazoles under mild reaction conditions with excellent regioselectivity [17,18,19,20]. In 2011, Zora and co-workers developed a method for the electrophilic cyclization of α,β-alkynic hydrazones mediated by CuI in the presence of trithylamine [21]. They also reported another study on the preparation of 4-iodopyrazoles promoted by molecular iodine [22]. In the same year, Liu, Xu, and co-workers declared a Au(I)-catalyzed tandem aminofluorination method to furnish fluoropyrazoles with the addition of selectfluor [23]. In 2017, Tsui et al. achieved a copper-mediated method for the synthesis of 4-(trifluoromethyl)pyrazoles [24]. In 2020, Niu and Gao et al. pioneered a facile method for the synthesis of 4-chalcogenylated pyrazoles [25]. Then, Wang and Ji’s group successfully utilized the strategy to synthesize 4-(arylselanyl)-1*H*-pyrazoles [26].

Despite such remarkable achievements, transition-metal catalysts and oxidants were always indispensable. Along with the concept of green chemistry, the significance of the green and sustainable development of chemical systems has gained increased attention; in particular, the development of eco-friendly synthesis methods that conform to the demands of green chemistry is of critical importance [27,28]. Thus, considerable effort has been devoted to the investigation of the green solvent system. Therefore, ionic liquids (ILs) have sparked great interest among chemists because of their unique physical and chemical properties, such as easy recyclability and high stability [29,30,31]. Inspired by the synthesis potential of ILs, we sought to develop a green and economical strategy to prepare useful pyrazoles with potential applications in various fields.

**Figure 1:**
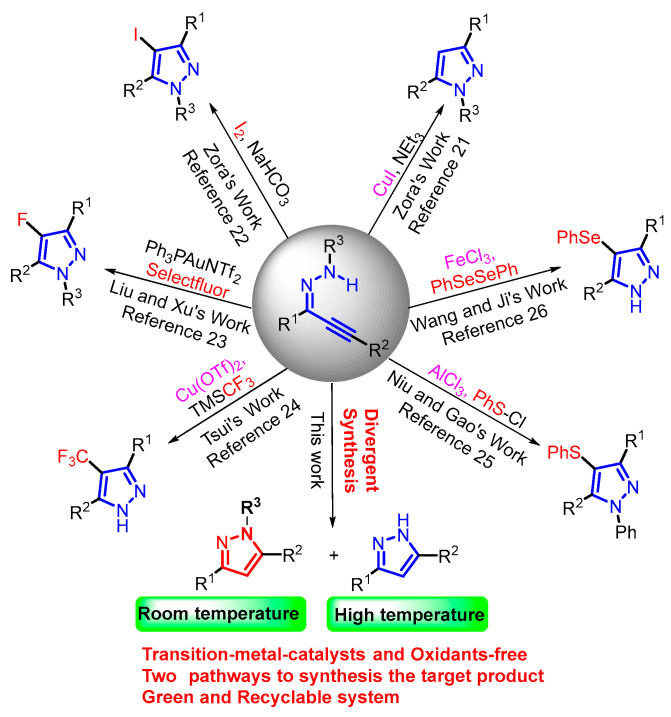
Strategies for the synthesis of pyrazole derivatives.

Herein, we report the solvent-switchable, metal- and oxidant-free divergent synthesis of 1*H*- and 1-tosyl-1*H*-pyrazoles via electrophilic cyclization, affording the desired products in moderate to excellent yields under mild conditions (Figure 1). 

## 2. Results and Discussion

The cyclization of 1,3-diphenylprop-2-yn-1-ylidene-4-methylbenzenesulfonohydrazide (**1a**) was initially investigated by changing the solvent amount. As exhibited in Table 1, the reaction did not proceed with conventional solvents such as CH_3_CN, EtOH, THF, or DMSO (entries 1–4). To our delight, a 95% product yield of 3,5-diphenyl-1-tosyl-1*H*-pyrazole (**2a**) was obtained when [HDBU][OAc] was screened. Encouraged by this result, ionic liquids containing different cations and anions like [HDBU][NHS], [HTMG][NHS], [HDBU][OAc][NHS], and [HTMG][HDBU][OAc]-[NHS] were then investigated, and excellent product yields were obtained at room temperature (entries 5–9). It was noticed that this transformation could also be realized in EtOH with the addition of 1.0 equivalent DBU (entry 4). Moreover, the desired product could be obtained via filtration rather than column chromatography, thus avoiding the wastage of organic solutions and silica gel (see Supporting Information). Since temperature is also a crucial factor in facilitating the reaction, different temperatures were investigated. To our surprise, when the temperature increased to 95 °C, no **2a** was detected, but **3a** was obtained in 85% yield, and further studies showed that 3a began to be produced at 40 °C. This result indicates that a divergent synthesis pathway could be achieved by regulating the reaction temperature. Further experiments showed that the transformation could also be achieved in different solvents, with [HDBU][OAc] demonstrating superior performance (entries 5–9). We also performed the reaction in the presence of different additives and reaction temperatures, and the yields decreased to varying degrees (see Supporting Information). Considering the green synthetic properties of EtOH, both pathways were executed in the following investigations.

With the aforementioned optimized reaction protocol in hand, the scope of cyclizations was first screened (Table 2). A wide range of substrates containing diverse substituents was evaluated using both optimal approaches, and the expected products were obtained in up to 98% (**2a**–**2u**) yields. No significant difference between substrates containing electron-donating (-Me, and -OMe) and electron-withdrawing groups (-F, -Cl, and -Br) on R1 and R2 rings was observed. Ulteriorly, 2-naphthyl, 2-thienyl, and saturated *t*-butyl (**2l**–**2n**) also gave satisfying yields of 67–83%. Furthermore, bis-pyrazoles (**2o**) could also be obtained with a maximum yield of 93%. However, it is a pity that the phenylhydrazine-substituted substrate (**2q**) could not take place in this transformation.

Subsequent studies were conducted to investigate the application scope for the synthesis of pyrazole derivatives under the standard reaction conditions (Table 3). Surprisingly, the reaction exhibited remarkable differences from the cyclization reaction, specifically manifested as follows: (1) the yields in ILs (35–88%) were significantly higher than those in EtOH (17–76%); (2) the substrates with electron-donating groups showed inferior reactivity to the electron-withdrawing ones, indicating that the substituent effect strongly influences the transformation; (3) none of corresponding products were obtained with aliphatic substrates and the corresponding cyclization product was retained.

**Scheme 1:**
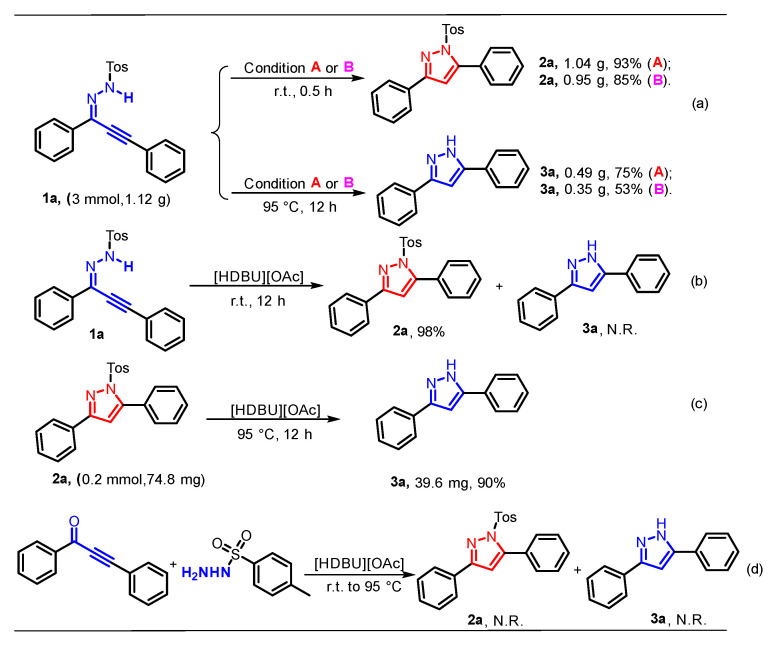
Gram-scale reaction and mechanistic studies.

Subsequently, a Gram-scale reaction was conducted under standard conditions, and the cyclization product **2a** was obtained in 93% and 85% yields. Additionally, the 1*H*-parazole (**3a**) could be furnished in 75% and 53% yields, respectively (Scheme 1a). Next, a range of control experiments were carried out to research the reaction mechanism. Initially, the cyclization was conducted in IL at room temperature for 12 h, and there was no **3a** produced, revealing that the reaction temperature is the key to triggering subsequent reactions (Scheme 1b). Then, **3a** was obtained at a 90% yield when **2a** was employed as the starting material under 95 °C, in IL for 12 h (Scheme 1c). Finally, none of the expected products were detected when 1,3-diphenylprop-2-yn-1-one and 4-methylbenzenesul-fonohydrazide were selected as initial materials (Scheme 1d).

**Figure 2:**
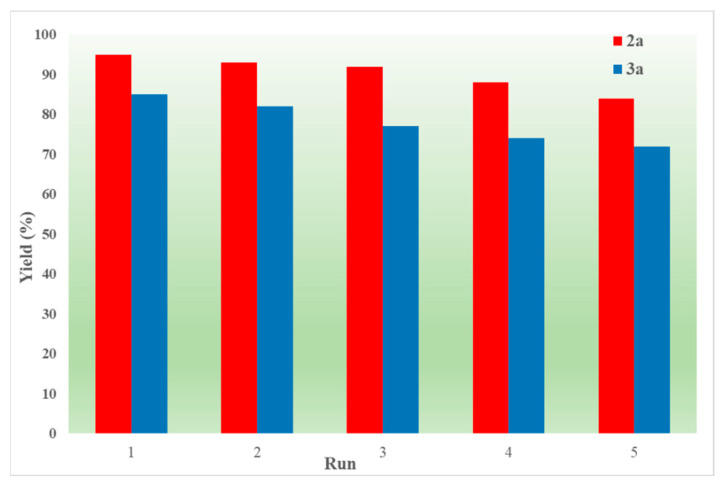
Recyclability test.

Finally, the recyclable experiment was further studied in order to assess the recyclability of IL (Figure 2). After the reaction was completed, the reaction mixture was poured into water and extracted with ethyl acetate. The organic layer containing the product was kept for purification, and the aqueous phase was placed in a drying cabinet to remove the excess water. Satisfactorily, only a minimal decline in activity was noted during five runs.

**Scheme 2:**
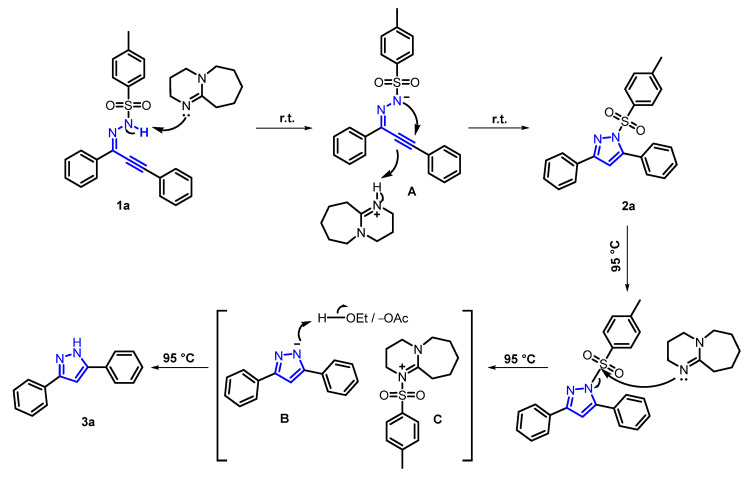
Plausible mechanistic pathway.

Based on the aforementioned experiments and previous related reports, a plausible mechanism for this reaction was proposed (Scheme 2). Primarily, in the presence of DBU, **1a** initiated electrophilic cyclization via nucleophilic attack of the secondary nitrogen atom to furnish protonated product **2a**, which ulteriorly underwent the nucleophilic attack of DBU triggered by thermal energy to obtain intermediates **B** and **C**. Eventually, B despoiled the hydrogen proton from the solvent to provide **3a**.

## 3. Experimental Section

All the chemicals were obtained commercially and used without any prior purification. ^1^H NMR and ^13^C NMR spectra were recorded on Bruker Avance II 400 or 500 spectrometers (See Supplementary Materials). All products were isolated by short chromatography on a silica gel (200–300 mesh) column using petroleum ether (60–90 °C) and ethyl acetate, unless otherwise noted. All compounds were characterized by ^1^H NMR and ^13^C NMR, which are consistent with those reported in the literature.

### Preparation of the Starting Materials

**Scheme 3:**
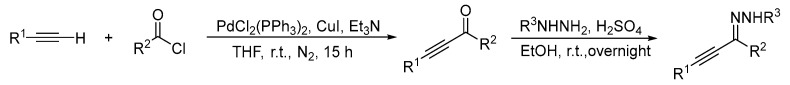
Preparation of the Starting Materials.

A mixture of acyl chloride (1a–1n, 1.3 equiv.; 1o 2.6 equiv.), PdCl_2_(PPh_3_)_2_ (1a–1n, 2 mol %; 1o 4 mol %), and Et_3_N (1a–1n, 1.5 equiv.; 1o 3.0 equiv.) in anhydrous THF were stirred for 10 min at room temperature under nitrogen. CuI (1a–1n, 4 mol %; 1o 8 mol %) was then added and stirred for another 10 min. Terminal alkyne (10 mmol) was then added and stirred at room temperature for 15 h. The resulting solution was extracted with EA and washed with 0.1N HCl. Next, the organic phase was dried over Na_2_SO_4_ and evaporated to give the crude product, which was purified by column chromatography using PE/EA as the eluent to give the desired *α*,*β*-alkynic ketones. Concentrated sulfuric acid (1a–1n, 1.1 equiv.; 1o 2.2 equiv.) was added dropwise to a slurry of *α*,*β*-alkynic ketone and hydrazine (1a–1n, 1.3 equiv.; 1o 2.6 equiv.) in EtOH at room temperature and stirred overnight. After the reaction was complete, the mixture was concentrated and the crude product was purified by column chromatography using PE/EA as the eluent to produce the *α*,*β*-alkynic hydrazone (Scheme 3).

**General procedure for synthesis of 2a**: Reaction conditions A: A mixture of the **1a** (0.2 mmol), [HDBU][OAc] (2.0 mL), stirred at r.t., under air, 0.5 h. Condition B: **1a** (0.2 mmol), DBU (1.0 equiv.), EtOH (2.0 mL), stirred at r.t., under air, 0.5 h. The product **2a** was purified by silica gel column flash chromatography using PE/AcOEt as an eluent.
**Procedure for Gram-scale synthesis of 2a:** Reaction conditions A: A mixture of the **1a** (3 mmol), [HDBU][OAc] (30.0 mL), r.t., under air, 0.5 h. Conditions B: A mixture of the **1a** (3 mmol), DBU (1.0 equiv.), EtOH (30.0 mL), r.t., under air, 0.5 h. The product **2a** was purified by silica gel column flash chromatography using PE/AcOEt as an eluent.
**Procedure for Gram-scale synthesis of 3a:** Reaction conditions A: A mixture of the **1a** (3 mmol), [HDBU][OAc] (30.0 mL), 95 °C, under air, 12 h. Conditions B: A mixture of the **1a** (3 mmol), DBU (1.0 equiv.), EtOH (30.0 mL), 95 °C, under air, 12 h. The product **3a** was purified by silica gel column flash chromatography using PE/AcOEt as an eluent.
**3,5-diphenyl-1-tosyl-1*H*-pyrazole (2a):** White solid, (A: 95%, 71.1 mg; B: 93%, 69.6 mg); ^1^H NMR (400 MHz, CDCl_3_) *δ* 7.87 (dd, *J* = 8.0, 1.4 Hz, 2H), 7.65 (d, *J* = 8.4 Hz, 2H), 7.53–7.39 (m, 8H), 7.22 (d, *J* = 8.2 Hz, 2H), 6.63 (s, 1H), 2.38 (s, 3H). ^13^C NMR (100 MHz, CDCl_3_) *δ* 155.19, 149.47, 145.33, 134.90, 131.38, 130.02, 129.66, 129.63, 129.48, 129.32, 128.72, 128.05, 127.83, 126.48, 109.52, 21.68. HRMS (ESI): Calculated for C_22_H_19_N_2_O_2_S: [M+H]^+^ 375.1162, Found 375.1165.
**5-phenyl-3-(p-tolyl)-1-tosyl-1*H*-pyrazole (2b):** White solid, (A: 98%, 76.0 mg; B: 95%, 73.7 mg); ^1^H NMR (400 MHz, CDCl_3_) *δ* 7.75 (d, *J* = 8.1 Hz, 2H), 7.63 (d, *J* = 8.4 Hz, 2H), 7.50–7.43 (m, 5H), 7.23–7.19 (m, 4H), 6.59 (s, 1H), 2.38 (s, 3H), 2.37 (s, 3H). ^13^C NMR (100 MHz, CDCl_3_) *δ* 155.34, 149.48, 145.22, 139.36, 134.93, 130.00, 129.70, 129.60, 129.42, 129.40, 128.56, 128.02, 127.80, 126.38, 109.53, 21.67, 21.40. HRMS (ESI): Calculated for C_23_H_21_N_2_O_2_S: [M+H]^+^ 389.1318, Found 389.1310.
**3-(4-chlorophenyl)-5-phenyl-1-tosyl-1*H*-pyrazole (2c):** White solid, (A: 92%, 75.1 mg; B: 90%, 73.4 mg) ^1^H NMR (400 MHz, CDCl_3_) *δ* 7.82–7.75 (m, 2H), 7.63 (d, *J* = 8.4 Hz, 2H), 7.52–7.42 (m, 5H), 7.41–7.34 (m, 2H), 7.22 (d, *J* = 8.1 Hz, 2H), 6.58 (s, 1H), 2.38 (s, 3H). ^13^C NMR (100 MHz, CDCl_3_) *δ* 153.94, 149.52, 145.47, 135.20, 134.79, 130.00, 129.92, 129.70, 129.56, 129.42, 128.93, 128.07, 127.85, 127.72, 109.25, 21.70. HRMS (ESI): Calculated for C_22_H_18_ClN_2_O_2_S: [M+H]^+^ 409.0772, Found 409.0775.
**3-(4-bromophenyl)-5-phenyl-1-tosyl-1*H*-pyrazole (2d):** White solid, (A: 93%, 84.1 mg; B: 89%, 80.4 mg); ^1^H NMR (400 MHz, CDCl_3_) *δ* 7.71 (d, *J* = 8.5 Hz, 2H), 7.62 (d, *J* = 8.3 Hz, 2H), 7.53 (d, *J* = 8.5 Hz, 2H), 7.47 (d, *J* = 3.3 Hz, 1H), 7.46–7.41 (m, 4H), 7.21 (d, *J* = 8.3 Hz, 2H), 6.57 (s, 1H), 2.37 (s, 3H). ^13^C NMR (100 MHz, CDCl_3_) *δ* 153.99, 149.53, 145.51, 134.72, 131.88, 130.34, 130.00, 129.72, 129.58, 129.37, 128.06, 127.98, 127.86, 123.48, 109.25, 21.72. HRMS (ESI): Calculated for C_22_H_18_BrN_2_O_2_S: [M+H]^+^ 453.0267, Found 453.0260.
**5-phenyl-3-(m-tolyl)-1-tosyl-1*H*-pyrazole (2e):** White solid, (A: 95%, 73.7 mg; B: 91%, 70.6 mg); ^1^H NMR (500 MHz, CDCl_3_) *δ* 7.71 (s, 1H), 7.61 (d, *J* = 8.2 Hz, 3H), 7.49–7.44 (m, 5H), 7.29 (t, *J* = 7.6 Hz, 1H), 7.19 (d, *J* = 8.2 Hz, 3H), 6.60 (s, 1H), 2.39 (s, 3H), 2.35 (s, 3H). ^13^C NMR (125 MHz, CDCl_3_) *δ* 155.41, 149.44, 145.28, 138.44, 134.88, 131.23, 130.13, 130.03, 129.64, 129.46, 128.60, 127.99, 127.81, 127.08, 123.64, 109.72, 21.69, 21.44. HRMS (ESI): Calculated for C_23_H_21_N_2_O_2_S: [M+H]^+^ 389.1318, Found 389.1317.
**3-(3-methoxyphenyl)-5-phenyl-1-tosyl-1*H*-pyrazole (2f):** White solid, (A: 97%, 78.3 mg; B: 93%, 75.1 mg); ^1^H NMR (400 MHz, CDCl_3_) *δ* 7.63 (d, *J* = 8.4 Hz, 2H), 7.51–7.43 (m, 5H), 7.41 (t, *J* = 4.9 Hz, 2H), 7.32 (t, *J* = 7.9 Hz, 1H), 7.21 (d, *J* = 8.2 Hz, 2H), 6.96–6.89 (m, 1H), 6.60 (s, 1H), 3.86 (s, 3H), 2.37 (s, 3H). ^13^C NMR (100 MHz, CDCl_3_) *δ* 159.89, 155.08, 149.44, 145.35, 134.83, 132.71, 130.01, 129.75, 129.66, 129.58, 129.48, 128.05, 127.82, 119.03, 115.17, 111.65, 109.70, 55.44, 21.70. HRMS (ESI): Calculated for C_23_H_21_N_2_O_3_S: [M+H]^+^ 405.1267, Found 405.1265.
**3-(3-fluorophenyl)-5-phenyl-1-tosyl-1*H*-pyrazole (2g):** White solid, (A: 93%, 72.9 mg; B: 91%, 71.3 mg); ^1^H NMR (400 MHz, CDCl_3_) *δ* 7.64 (d, *J* = 8.4 Hz, 2H), 7.62–7.55 (m, 2H), 7.52–7.43 (m, 5H), 7.40–7.35 (m, 1H), 7.23 (d, *J* = 8.2 Hz, 2H), 7.09–7.04 (m, 1H), 6.59 (s, 1H), 2.38 (s, 3H). ^13^C NMR (100 MHz, CDCl_3_) *δ* 164.26 (d, *J* = 245.0 Hz), 153.85, 149.45, 145.50, 134.75, 133.63 (d, *J* = 8.0 Hz), 130.31 (d, *J* = 8.0 Hz), 129.99, 129.70, 129.56, 129.38, 128.10, 127.84, 122.11 (d, *J* = 2.0 Hz), 116.21 (d, *J* = 21.0 Hz), 113.43 (d, *J* = 21.0 Hz), 109.31, 21.69. HRMS (ESI): Calculated for C_22_H_18_FN_2_O_2_S: [M+H]^+^ 393.1068, Found 393.1066.
**5-(4-methoxyphenyl)-3-phenyl-1-tosyl-1*H*-pyrazole (2h):** White solid, (A: 94%, 76.0 mg; B: 92%, 74.3 mg); ^1^H NMR (400 MHz, CDCl_3_) *δ* 7.89–7.82 (m, 2H), 7.62 (d, *J* = 8.4 Hz, 2H), 7.45–7.35 (m, 5H), 7.20 (d, *J* = 8.2 Hz, 2H), 7.02–6.92 (m, 2H), 6.57 (s, 1H), 3.88 (s, 3H), 2.36 (s, 3H). ^13^C NMR (100 MHz, CDCl_3_) *δ* 160.56, 155.27, 149.56, 145.26, 134.88, 131.43, 131.38, 129.63, 129.29, 128.70, 127.99, 126.47, 121.72, 113.29, 109.29, 55.38, 21.69. HRMS (ESI): Calculated for C_23_H_21_N_2_O_3_S: [M+H]^+^ 405.1267, Found 405.1260.
**5-(3-fluorophenyl)-3-phenyl-1-tosyl-1*H*-pyrazole (2i):** White solid, (A: 91%, 71.3 mg; B: 88%, 69.0 mg); ^1^H NMR (500 MHz, CDCl_3_) *δ* 7.77–7.75 (m, 2H), 7.58 (d, *J* = 8.4 Hz, 2H), 7.37–7.29 (m, 4H), 7.19–7.14 (m, 3H), 7.13–7.06 (m, 2H), 6.55 (s, 1H), 2.30 (s, 3H). ^13^C NMR (125 MHz, CDCl_3_) *δ* 161.89 (d, *J* = 245.0 Hz), 154.15, 146.80 (d, *J* = 2.5 Hz), 144.50, 133.63, 130.45 (d, *J* = 7.5 Hz), 130.09, 128.69, 128.41, 128.36 (d, *J* = 2.5 Hz), 127.69, 126.99, 125.39, 124.88, 124.86, 116.09 (d, *J* = 22.5 Hz), 115.47 (d, *J* = 20.0 Hz), 108.69, 20.64. HRMS (ESI): Calculated for C_22_H_18_FN_2_O_2_S: [M+H]^+^ 393.1068, Found 393.1064.
**5-(3,4-dichlorophenyl)-3-phenyl-1-tosyl-1*H*-pyrazole (2j):** White solid, (A: 92%, 81.3 mg; B: 87%, 76.9 mg); ^1^H NMR (500 MHz, CDCl_3_) *δ* 7.76–7.74 (m, 2H), 7.59 (d, *J* = 8.4 Hz, 2H), 7.46 (d, *J* = 8.3 Hz, 1H), 7.41 (d, *J* = 2.0 Hz, 1H), 7.37–7.31 (m, 3H), 7.27 (dd, *J* = 8.3, 2.0 Hz, 1H), 7.20–7.16 (m, 2H), 6.55 (s, 1H), 2.32 (s, 3H). ^13^C NMR (125 MHz, CDCl_3_) *δ* 154.22, 145.55, 144.70, 133.52, 132.86, 131.10, 130.40, 129.93, 128.88, 128.79, 128.46, 128.39, 127.72, 127.01, 125.39, 108.81, 20.68. HRMS (ESI): Calculated for C_22_H_17_Cl_2_N_2_O_2_S: [M+H]^+^ 443.0382, Found 443.0381.
**5-(3,5-dimethoxyphenyl)-3-phenyl-1-tosyl-1*H*-pyrazole (2k):** White solid, (A: 98%, 85.0 mg; B: 96%, 83.3 mg); ^1^H NMR (400 MHz, DMSO-*_d6_*) *δ* 7.82 (d, *J* = 7.0 Hz, 2H), 7.59 (d, *J* = 8.1 Hz, 2H), 7.46–7.32 (m, 5H), 7.11 (s, 1H), 6.61–6.57 (m, 3H), 3.75 (s, 6H), 2.29 (s, 3H). ^13^C NMR (100 MHz, DMSO-*_d6_*) *δ* 160.50, 159.78, 154.74, 149.12, 145.92, 134.01, 130.86, 130.72, 130.17, 129.64, 128.99, 127.54, 126.16, 110.14, 108.06, 101.35, 55.45, 21.16. HRMS (ESI): Calculated for C_24_H_23_N_2_O_4_S: [M+H]^+^ 435.1373, Found 435.1377.
**3-(naphthalen-2-yl)-5-phenyl-1-tosyl-1*H*-pyrazole (2l):** White solid, (A: 98%, 83.1 mg; B: 95%, 80.5 mg); ^1^H NMR (500 MHz, CDCl_3_) *δ* 8.30 (s, 1H), 8.04 (dd, *J* = 8.6, 1.5 Hz, 1H), 7.89 (d, *J* = 8.6 Hz, 2H), 7.85 (dd, *J* = 6.6, 2.7 Hz, 1H), 7.67 (d, *J* = 8.4 Hz, 2H), 7.54–7.46 (m, 7H), 7.22 (d, *J* = 8.3 Hz, 2H), 6.76 (s, 1H), 2.36 (s, 3H). ^13^C NMR (125 MHz, CDCl_3_) *δ* 154.14, 148.51, 144.29, 133.77, 132.68, 132.18, 128.97, 128.60, 128.52, 128.45, 127.67, 127.39, 127.34, 126.96, 126.77, 126.73, 125.58, 125.43, 124.75, 122.98, 108.71, 20.61. HRMS (ESI): Calculated for C_26_H_21_N_2_O_2_S: [M+H]^+^ 425.1318, Found 425.1311.
**5-phenyl-3-(thiophen-2-yl)-1-tosyl-1*H*-pyrazole (2m):** White solid, (A: 95%, 71.0 mg; B: 86%, 64.3 mg); ^1^H NMR (400 MHz, DMSO-*_d6_*) *δ* 7.61–7.58 (m, 2H), 7.52–7.46 (m, 7H), 7.36 (d, *J* = 7.3 Hz, 2H), 7.11 (t, *J* = 4.9 Hz, 1H), 7.04 (s, 1H), 2.31 (s, 3H). ^13^C NMR (100 MHz, DMSO-*_d6_*) *δ* 150.76, 149.67, 145.92, 133.83, 133.35, 130.17, 129.77, 128.95, 128.04, 127.95, 127.39, 110.16, 21.14. HRMS (ESI): Calculated for C_22_H_19_N_2_O_2_S: [M+H]^+^ 375.1089, Found 375.1082.
**3-(tert-butyl)-5-phenyl-1-tosyl-1*H*-pyrazole (2n):** White solid, (A: 90%, 63.7 mg; B: 85%, 60.2 mg); ^1^H NMR (500 MHz, CDCl_3_) *δ* 7.55 (d, *J* = 8.3 Hz, 2H), 7.44–7.40 (m, 5H), 7.19 (d, *J* = 8.2 Hz, 2H), 6.19 (s, 1H), 2.38 (s, 3H), 1.26 (s, 9H). ^13^C NMR (125 MHz, CDCl_3_) *δ* 166.01, 148.46, 143.78, 133.74, 128.92, 128.77, 128.23, 128.15, 126.82, 126.65, 108.86, 31.60, 28.73, 20.60. HRMS (ESI): Calculated for C_20_H_23_N_2_O_2_S: [M+H]^+^ 355.1475, Found 355.1479.
**1,3-bis(5-phenyl-1-tosyl-1*H*-pyrazol-3-yl)benzene (2o):** White solid, (A: 93%, 124.6 mg; B: 84%, 112.5 mg); ^1^H NMR (400 MHz, CDCl_3_) *δ* 8.24 (s, 1H), 7.89 (d, *J* = 7.6 Hz, 2H), 7.62 (d, *J* = 8.1 Hz, 4H), 7.49–7.46 (m, 11H), 7.21 (d, *J* = 8.1 Hz, 4H), 6.69 (s, 2H), 2.37 (s, 6H). ^13^C NMR (100 MHz, CDCl_3_) *δ* 154.77, 149.55, 145.44, 134.77, 131.92, 130.04, 129.70, 129.54, 129.48, 129.15, 128.03, 127.85, 127.34, 124.49, 109.78, 21.71. HRMS (ESI): Calculated for C_38_H_31_N_4_O_4_S_2_: [M+H]^+^ 671.1781, Found 671.1788.
**3,5-diphenyl-1-(phenylsulfonyl)-1*H*-pyrazole (2p):** White solid, (A: 93%, 66.9 mg; B: 85%, 61.2 mg); ^1^H NMR (400 MHz, CDCl_3_) *δ* 7.86 (dd, *J* = 7.9, 1.4 Hz, 2H), 7.77–7.73 (m, 2H), 7.58–7.55 (m, 1H), 7.51–7.48 (m, 1H), 7.57–7.45 (m, 5H), 7.43–7.39 (m, 4H), 6.63 (s, 1H). ^13^C NMR (100 MHz, CDCl_3_) *δ* 155.35, 149.56, 137.80, 134.12, 131.25, 130.00, 129.53, 129.47, 129.38, 129.01, 128.72, 127.97, 127.85, 126.47, 109.63. HRMS (ESI): Calculated for C_21_H_17_N_2_O_2_S: [M+H]^+^ 361.1005, Found 361.1008.
**General procedure for synthesis of 3a**: Reaction conditions A: A mixture of the **1a** (0.2 mmol), [HDBU][OAc] (2.0 mL), stirred at 95 °C, under air, 12 h. Reaction conditions A for **3a**: A mixture of the **1a** (0.2 mmol), DBU (1.0 equiv.), EtOH (2.0 mL), stirred at 95 °C, under air, 12 h. The product **3a** was purified by silica gel column flash chromatography using PE/AcOEt as an eluent.
**3,5-diphenyl-1*H*-pyrazole (3a):** White solid, (A: 85%, 37.4 mg; B: 65%, 28.6 mg); ^1^H NMR (400 MHz, DMSO-*_d6_*) *δ* 13.33 (s, 1H), 7.81 (d, *J* = 7.4 Hz, 2H), 7.74 (d, *J* = 7.5 Hz, 2H), 7.41–7.33 (m, 4H), 7.29–7.21 (m, 2H), 7.12 (s, 1H). ^13^C NMR (100 MHz, DMSO-*_d6_*) *δ* 151.34, 143.39, 133.69, 129.36, 129.06, 128.67, 128.17, 127.49, 125.14, 99.66. HRMS (ESI): Calculated for C_15_H_13_N_2_: [M+H]^+^ 221.1073, Found 221.1079.
**5-phenyl-3-(p-tolyl)-1*H*-pyrazole (3b):** White solid, (A: 70%, 32.7 mg; B: 53%, 24.8 mg); ^1^H NMR (400 MHz, DMSO-*_d6_*) *δ* 13.24 (s, 1H), 7.79 (d, *J* = 7.5 Hz, 2H), 7.68 (d, *J* = 7.9 Hz, 2H), 7.40 (t, *J* = 7.6 Hz, 2H), 7.28 (t, *J* = 7.3 Hz, 1H), 7.21 (d, *J* = 7.9 Hz, 2H), 7.08 (s, 1H), 2.28 (s, 3H). ^13^C NMR (100 MHz, DMSO-*_d6_*) *δ* 137.12, 129.40, 128.91, 128.82, 127.71, 125.10, 125.05, 99.29, 20.86. HRMS (ESI): Calculated for C_16_H_15_N_2_: [M+H]^+^ 235.1230, Found 235.1233.
**3-(4-chlorophenyl)-5-phenyl-1*H*-pyrazole (3c):** White solid, (A: 83%, 42.1 mg; B: 61%, 30.9 mg); ^1^H NMR (400 MHz, DMSO-*_d6_*) *δ* 13.44 (s, 1H), 7.89–7.78 (m, 4H), 7.65–7.62 (m, 2H), 7.49–7.47 (m, 2H), 7.35 (s, 1H), 7.22 (s, 1H). ^13^C NMR (100 MHz, DMSO-*_d6_*) *δ* 131.64, 128.99, 127.09, 125.12, 99.86. HRMS (ESI): Calculated for C_15_H_12_ClN_2_: [M+H]^+^ 255.0684, Found 255.0689.
**5-phenyl-3-(m-tolyl)-1*H*-pyrazole (3d):** White solid, (A: 74%, 34.6 mg; B: 58%, 27.1 mg); ^1^H NMR (400 MHz, DMSO-*_d6_*) *δ* 13.25 (s, 1H), 7.71 (d, *J* = 7.1 Hz, 2H), 7.55 (s, 1H), 7.50 (d, *J* = 7.1 Hz, 1H), 7.29 (t, *J* = 7.5 Hz, 2H), 7.17 (t, *J* = 7.3 Hz, 2H), 7.03 (s, 1H), 6.98 (d, *J* = 7.3 Hz, 1H), 2.20 (s, 3H). ^13^C NMR (100 MHz, DMSO-*_d6_*) *δ* 138.02, 128.91, 127.84, 125.79, 125.18, 122.38, 99.67, 21.21. HRMS (ESI): Calculated for C_16_H_15_N_2_: [M+H]^+^ 235.1230, Found 235.1234.
**3-(3-methoxyphenyl)-5-phenyl-1*H*-pyrazole (3e):** White solid, (A: 69%, 34.5 mg; B: 49%, 24.5 mg); ^1^H NMR (400 MHz, DMSO-*_d6_*) *δ* 13.30 (s, 1H), 7.77 (d, *J* = 7.4 Hz, 2H), 7.39–7.35 (m, 4H), 7.30–7.23 (m, 2H), 7.14 (s, 1H), 6.83 (d, *J* = 7.6 Hz, 1H), 3.74 (s, 3H). ^13^C NMR (100 MHz, DMSO-*_d6_*) *δ* 159.70, 129.96, 128.84, 127.79, 125.14, 117.56, 113.44, 110.53, 99.91, 55.17. HRMS (ESI): Calculated for C_16_H_15_N_2_O: [M+H]^+^ 251.1179, Found 251.1171.
**3-(3-chlorophenyl)-5-phenyl-1*H*-pyrazole (3f):** White solid, (A: 80%, 40.6 mg; B: 70%, 35.5 mg); ^1^H NMR (400 MHz, DMSO-*_d6_*) *δ* 13.33 (s, 1H), 7.78–7.35 (m, 4H), 7.40 (d, *J* = 8.3 Hz, 2H), 7.35 (t, *J* = 7.6 Hz, 2H), 7.24 (t, *J* = 7.4 Hz, 1H), 7.10 (s, 1H). ^13^C NMR (100 MHz, DMSO-*_d6_*) *δ* 132.23, 129.40, 128.90, 128.85, 127.97, 126.82, 125.16, 125.07, 99.90. HRMS (ESI): Calculated for C_15_H_12_ClN_2_: [M+H]^+^ 255.0684, Found 255.0679.
**3-(3-fluorophenyl)-5-phenyl-1*H*-pyrazole (3g):** White solid, (A: 88%, 41.8 mg; B: 75%, 35.7 mg); ^1^H NMR (400 MHz, DMSO-*_d6_*) *δ* 13.38 (s, 1H), 7.74 (d, *J* = 7.2 Hz, 2H), 7.62–7.57 (m, 2H), 7.41–7.34 (m, 3H), 7.24 (t, *J* = 7.3 Hz, 1H), 7.17 (s, 1H), 7.06 (t, *J* = 7.7 Hz, 1H). ^13^C NMR (100 MHz, DMSO-*_d6_*) *δ* 163.87 (d, *J* = 240.0 Hz), 130.84, 128.93, 128.02, 125.17, 121.21 (d, *J* = 3.0 Hz), 114.49 (d, *J* = 20.0 Hz), 111.82 (d, *J* = 23.0 Hz), 100.24. HRMS (ESI): Calculated for C_15_H_12_FN_2_: [M+H]^+^ 239.0979, Found 239.0977.
**5-(4-methoxyphenyl)-3-phenyl-1*H*-pyrazole (3h):** White solid, (A: 70%, 35.0 mg; B: 55%, 27.5 mg); ^1^H NMR (400 MHz, DMSO-*_d6_*) *δ* 13.16 (s, 1H), 7.81–7.60 (m, 4H), 7.32 (t, *J* = 7.3 Hz, 2H), 7.20 (t, *J* = 7.0 Hz, 1H), 6.95 (s, 1H), 6.90 (d, *J* = 8.1 Hz, 2H), 3.66 (s, 3H). ^13^C NMR (100 MHz, DMSO-*_d6_*) *δ* 159.06, 151.20, 143.36, 133.72, 128.81, 127.65, 126.52, 125.13, 114.28, 98.87, 55.16. HRMS (ESI): Calculated for C_16_H_15_N_2_O: [M+H]^+^ 251.1179, Found 251.1173.
**5-(4-ethylphenyl)-3-phenyl-1*H*-pyrazole (3i):** White solid, (A: 78%, 38.6 mg; B: 58%, 28.7 mg); ^1^H NMR (400 MHz, DMSO-*_d6_*) *δ* 13.22 (s, 1H), 7.71 (d, *J* = 7.1 Hz, 2H), 7.60 (d, *J* = 7.5 Hz, 2H), 7.28 (t, *J* = 7.5 Hz, 2H), 7.16 (t, *J* = 7.3 Hz, 1H), 7.10 (d, *J* = 7.8 Hz, 2H), 6.97 (s, 1H), 2.43 (q, *J* = 7.6 Hz, 2H), 1.01 (t, *J* = 7.6 Hz, 3H). ^13^C NMR (100 MHz, DMSO-*_d6_*) *δ* 143.53, 128.88, 128.30, 127.77, 125.21, 125.18, 99.35, 28.07, 15.63. HRMS (ESI): Calculated for C_17_H_17_N_2_: [M+H]^+^ 249.1386, Found 249.1388.
**5-(3-fluorophenyl)-3-phenyl-1*H*-pyrazole (3j):** White solid, (A: 87%, 41.4 mg; B: 66%, 31.4 mg); ^1^H NMR (400 MHz, DMSO-*_d6_*) *δ* 13.41 (s, 1H), 7.85–7.55 (m, 4H), 7.39–7.30 (m, 3H), 7.23 (d, *J* = 24.2 Hz, 2H), 7.07 (s, 1H). ^13^C NMR (100 MHz, DMSO-*_d6_*) *δ* 163.96, (d, *J* = 242.0 Hz), 130.92 (d, *J* = 3.0 Hz), 128.99, 128.06, 125.25, 121.26, 114.36 (d, *J* = 21.0 Hz), 111.91 (d, *J* = 23.0 Hz), 100.27. HRMS (ESI): Calculated for C_15_H_12_FN_2_: [M+H]^+^ 239.0979, Found 239.0982.
**5-(3,4-dichlorophenyl)-3-phenyl-1*H*-pyrazole (3k):** White solid, (A: 87%, 50.1 mg; B: 71%, 40.8 mg); ^1^H NMR (400 MHz, DMSO-*_d6_*) *δ* 13.23 (s, 1H), 7.73 (d, *J* = 7.6 Hz, 2H), 7.39–7.35 (m, 4H), 7.26 (t, *J* = 7.3 Hz, 1H), 7.04–7.02 (m, 1H), 6.96 (s, 1H). ^13^C NMR (100 MHz, DMSO-*_d6_*) *δ* 128.95, 128.12, 127.77, 125.20, 123.95, 99.57. HRMS (ESI): Calculated for C_15_H_12_Cl_2_N_2_: [M+H]^+^ 289.0298, Found 289.0295.
**5-(3,5-dimethoxyphenyl)-3-phenyl-1*H*-pyrazole (3l):** White solid, (A: 35%, 19.6 mg; B: 17%, 9.5 mg); ^1^H NMR (400 MHz, DMSO-*_d6_*) *δ* 7.90–7.87 (m, 2H), 7.48–7.44 (m, 2H), 7.36–7.32 (m, 1H), 7.26 (s, 1H), 7.09 (d, *J* = 2.3 Hz, 2H), 6.51 (t, *J* = 2.2 Hz, 1H), 3.83 (s, 6H). ^13^C NMR (100 MHz, DMSO-*_d6_*) *δ* 160.89, 147.40, 133.27, 131.75, 128.82, 127.76, 125.15, 103.27, 100.08, 99.80, 55.32. HRMS (ESI): Calculated for C_17_H_17_N_2_O_2_: [M+H]^+^ 281.1285, Found 281.1280.
**3-(naphthalen-2-yl)-5-phenyl-1H-pyrazole (3m):** White solid, (A: 83%, 44.8 mg; B: 72%, 38.8 mg); ^1^H NMR (400 MHz, DMSO-*_d6_*) *δ* 13.43 (s, 1H), 8.32 (s, 1H), 8.08–7.66 (m, 6H), 7.53–7.35 (m, 4H), 7.29 (s, 2H). ^13^C NMR (100 MHz, DMSO-*_d6_*) *δ* 133.22, 132.56, 128.98, 128.42, 128.03, 127.77, 126.67, 126.12, 125.18, 123.72, 123.50, 100.11. HRMS (ESI): Calculated for C_19_H_15_N_2_: [M+H]^+^ 271.1230, Found 271.1239.
**5-phenyl-3-(thiophen-2-yl)-1*H*-pyrazole (3n):** White solid, (A: 77%, 34.8 mg; B: 62%, 28.0 mg); ^1^H NMR (400 MHz, DMSO-*_d6_*) *δ* 13.43 (s, 1H), 7.90 (d, *J* = 7.5 Hz, 2H), 7.56–7.53 (m, 4H), 7.43 (t, *J* = 7.3 Hz, 1H), 7.25–7.17 (m, 1H), 7.14 (s, 1H). ^1^3C NMR (100 MHz, DMSO-*_d6_*) *δ* 129.03, 128.20, 127.84, 125.23, 124.00, 99.61. HRMS (ESI): Calculated for C_13_H_11_N_2_S: [M+H]^+^ 227.0637, Found 227.0630.

## 4. Conclusions

This report depicted a novel solvent-switchable and thermodynamic-controlled divergent synthesis reaction for the synthesis of 1*H*- and 1-sulfonyl pyrazoles under transition-metal-catalyst and oxidant conditions. As demonstrated, various substrates could take place during the transformation smoothly, and provided the corresponding products in moderate to good yields through the model systems. Control experiments showed that reaction temperature was a pivotal trigger process for the electrophilic cyclization reaction.

## Figures and Tables

**Table 1:** Optimization of reaction conditions ^a,b^.

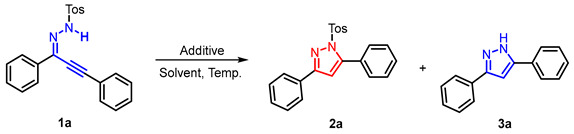			
Entry	Solvent	Yield of 2a [%] ^b^	Yield of 3a [%] ^b^
1	CH_3_CN	N.R.	N.R.
2	DMSO	N.R.	N.R.
3	THF	N.R.	N.R.
4	EtOH	N.R. (95) ^d^	N.R. (65) ^e^
5	[HDBU][OAc]	98	85 ^c^
6	[HDBU][NHS]	95	78 ^c^
7	[HTMG][NHS]	78	63 ^c^
8	[HDBU][OAc][NHS]	86	68 ^c^
9	[HTMG][HDBU][OAc][NHS]	80	58 ^c^

[a] Reaction conditions: **1a** (0.2 mmol), solvent (2.0 mL), r.t., 0.5 h; [b] Isolated yields. [c] 95 °C, 12 h. [d] DBU (1.0 equiv.) additive, r.t., 0.5 h. [e] DBU (1.0 equiv.) additive, 95 °C, 12 h. [HDBU] = 2,3,4,-6,7,8,9,10-octahydropyrimido [1,2-*a*]azepin-1-ium, [NHS] = 2,5-dioxopyrrolidin-1-olate, [HTMG] = bis(dimethylamino)-methaniminium.

**Table 2:** Scope of cyclization substrates ^a,b^.

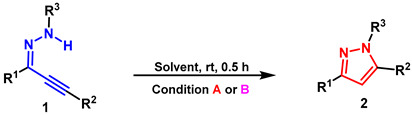			
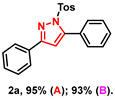	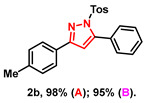	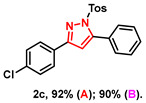	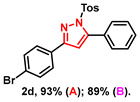
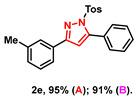	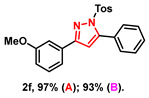	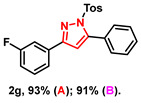	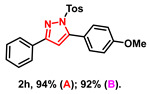
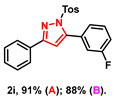	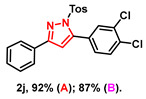	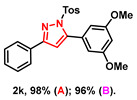	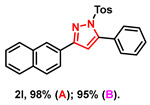
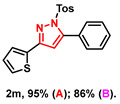	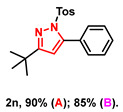	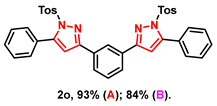	
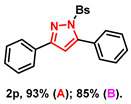	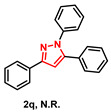		

[a] Reaction condition A: **1** (0.2 mmol), [HDBU][OAc] (2.0 mL), stirred at r.t., under air, 0.5 h. Condition B: **1** (0.2 mmol), DBU (1.0 equiv.), EtOH (2.0 mL), stirred at r.t., under air, 0.5 h. [b] Isolated yields.

**Table 3:** Scope of pyrazole substrates ^a,b^.

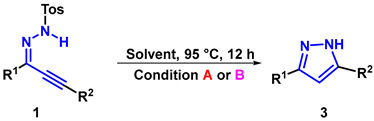			
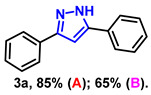	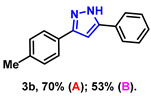	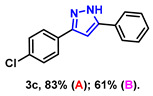	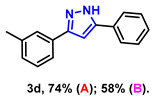
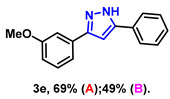	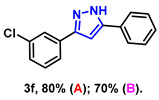	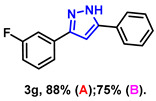	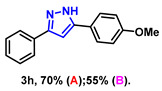
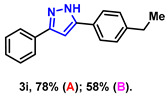	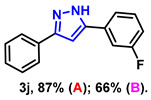	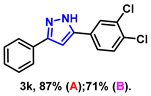	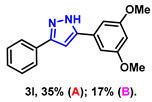
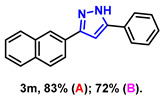	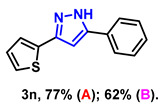	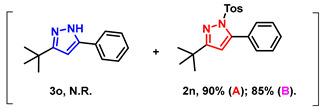	

[a] Reaction condition A: **1** (0.2 mmol), [HDBU][OAc] (2.0 mL), stirred at 95 °C, under air, 12 h. Condition B: **1** (0.2 mmol), DBU (1.0 equiv.), EtOH (2.0 mL), stirred at 95 °C, under air, 12 h. [b] Isolated yields.

## Data Availability

See Supporting Information.

## Supplementary Materials

The following supporting information can be downloaded at: https://www.mdpi.com/article/10.3390/molecules29081706/s1. Figure S1: ^1^H NMR Spectra of Compound 2a; Figure S2: ^13^C NMR Spectra of Compound 2a; Figure S3: ^1^H NMR Spectra of Compound 2b; Figure S4:^13^C NMR Spectra of Compound 2b; Figure S5: ^1^H NMR Spectra of Compound 2c; Figure S6: ^13^CNMR Spectra of Compound 2c; Figure S7: ^1^H NMR Spectra of Compound 2d; Figure S8: ^13^C NMR Spectra of Compound 2d; Figure S9: ^1^H NMR Spectra of Compound 2e; Figure S10: ^13^C NMR Spectra of Compound 2e; Figure S11: ^1^H NMR Spectra of Compound 2f; Figure S12: ^13^C NMR Spectra of Compound 2f; Figure S13: ^1^H NMR Spectra of Compound 2g; Figure S14: ^13^C NMR Spectra of Compound 2g; Figure S15: ^1^H NMR Spectra of Compound 2h; Figure S16: ^13^C NMR Spectra of Compound 2h; Figure S17: ^1^H NMR Spectra of Compound 2i; Figure S18: ^13^C NMR Spectra of Compound 2i; Figure S19: ^1^H NMR Spectra of Compound 2j; Figure S20: ^13^C NMR Spectra of Compound 2j; Figure S21: ^1^H NMR Spectra of Compound 2k; Figure S22: ^13^C NMR Spectra of Compound 2k; Figure S23: ^1^H NMR Spectra of Compound 2l; Figure S24: ^13^C NMR Spectra of Compound 2l; Figure S25: ^1^H NMR Spectra of Compound 2m; Figure S26:^13^C NMR Spectra of Compound 2m; Figure S27: ^1^H NMR Spectra of Compound 2n; Figure S28: ^13^C NMR Spectra of Compound 2n; Figure S29: ^1^H NMR Spectra of Compound 2o; Figure S30: ^13^C NMR Spectra of Compound 2o; Figure S31: ^1^H NMR Spectra of Compound 2p; Figure S32: ^13^C NMR Spectra of Compound 2p; Figure S33: ^1^H NMR Spectra of Compound 3a; Figure S34: ^13^C NMR Spectra of Compound 3a; Figure S35: ^1^H NMR Spectra of Compound 3b; Figure S36: ^13^C NMR Spectra of Compound 3b; Figure S37: ^1^H NMR Spectra of Compound 3c; Figure S38: ^13^C NMR Spectra of Compound 3c; Figure S39: ^1^H NMR Spectra of Compound 3d; Figure S40: ^13^C NMR Spectra of Compound 3d; Figure S41: ^1^H NMR Spectra of Compound 3e; Figure S42: ^13^C NMR Spectra of Compound 3e; Figure S43: ^1^H NMR Spectra of Compound 3f; Figure S44: ^13^C NMR Spectra of Compound 3f; Figure S45: ^1^H NMR Spectra of Compound 3g; Figure S46: ^13^C NMR Spectra of Compound 3g; Figure S47: ^1^H NMR Spectra of Compound 3h; Figure S48: ^13^C NMR Spectra of Compound 3h; Figure S49: ^1^H NMR Spectra of Compound 3i; Figure S50: ^13^C NMR Spectra of Compound 3i; Figure S51: ^1^H NMR Spectra of Compound 3j; Figure S52: ^13^C NMR Spectra of Compound 3j; Figure S53: ^1^H NMR Spectra of Compound 3k; Figure S54: ^13^C NMR Spectra of Compound 3k; Figure S55: ^1^H NMR Spectra of Compound 3l; Figure S56: ^13^C NMR Spectra of Compound 3l; Figure S57: ^1^H NMR Spectra of Compound 3m; Figure S58:^13^C NMR Spectra of Compound 3m; Figure S59: ^1^H NMR Spectra of Compound 3n; Figure S60: ^13^C NMR Spectra of Compound 3n.

## References

[1] Lan R.X., Liu Q., Fan P.S., Lin S., Fernando S.R., McCallion D., Pertwee R., Makriyannis A. (1999). Structure−activity relationships of pyrazole derivatives as cannabinoid receptor antagonists. J. Med. Chem..

[2] Daidone G., Maggio B., Plescia S., Raffa D., Musiu C., Milia C., Perra G., Marongiu M.E. (1998). Antimicrobial and antineoplastic activities of new 4-diazopyrazole derivatives. Eur. J. Med. Chem..

[3] Haque T.S., Tadesse S., Marcinkeviciene J., Rogers M.J., Sizemore C., Kopcho L.M., Amsler K., Ecret L.D., Zhan D.L., Hobbs F. (2002). Parallel Synthesis of Potent, Pyrazole-Based Inhibitors of Helicobacter pylori Dihydroorotate Dehydrogenase. J. Med. Chem..

[4] Castagnolo D., Manetti F., Radi M., Bechi B., Pagano M., Logu A., Meleddu R., Saddi M., Botta M. (2009). Synthesis, biological evaluation, and SAR study of novel pyrazole analogues as inhibitors of Mycobacterium tuberculosis: Part 2. Synthesis of rigid pyrazolones. Bioorg. Med. Chem..

[5] Moore K.W., Bonner K., Jones E.A., Emms F., Leeson P.D., Marwood R., Patel S., Patel S., Rowley M., Thomas S. (1999). 4-*N*-*linked*-heterocyclic piperidine derivatives with high affinity and selectivity for human dopamine D_4_ receptors. Bioorg. Med. Chem..

[6] Bekhit A.A., Ashour H.M.A., Guemei A.A. (2005). Novel Pyrazole Derivatives as Potential Promising Anti-inflammatory Antimicrobial Agents. Arch. Pharm. Chem. Life Sci..

[7] Almansa C., Gómez L.A., Cavalcanti F.L., Arriba A.F., García-Rafanell J., Forn J. (1997). Synthesis and Structure−Activity Relationship of a New Series of Potent AT1 Selective Angiotensin II Receptor Antagonists:  5-(Biphenyl-4-yl-methyl)pyrazoles. J. Med. Chem..

[8] Silvestri R., Ligresti A., La Regina G., Piscitelli F., Gatti V., Brizzi A., Pasquini S., Lavecchia A., Allarà M., Fantini N.M. (2009). Synthesis, cannabinoid receptor affinity, molecular modeling studies and in vivo pharmacological evaluation of new substituted 1-aryl-5-(1*H*-pyrrol-1-yl)-1*H*-pyrazole-3-carboxamides. 2. Effect of the 3-carboxamide substituent on the affinity and selectivity profile. Bioorg. Med. Chem..

[9] Kees K.L., Fitzgerald J.J., Steiner K.E., Mattes J.F., Mihan B., Tosi T., Mondoro D., McCaleb M.L. (1996). New potent antihyperglycemic agents in db/db mice: Synthesis and structure-activity relationship studies of (4-substituted benzyl) (trifluoromethyl)pyrazoles and -pyrazolones. J. Med. Chem..

[10] Zhang Y.Q., Wu C.Y., Zhang N.N., Fan R., Ye Y., Xu J. (2023). Recent advances in the development of pyrazole derivatives as anticancer agents. Int. J. Mol. Sci..

[11] Grotjahn D.B., Van S., Combs D., Lev D.A., Schneider C., Rideout M., Meyer C., Hernandez G., Mejorado L. (2002). New flexible synthesis of pyrazoles with different, functionalized substituents at C3 and C5. J. Org. Chem..

[12] Dastrup D.M., Yap A.H., Weinreb S.M., Henryb J.R., Lechleiter A.J. (2004). Synthesis of *β*-tosylethylhydrazine and its use in preparation of *N*-protected pyrazoles and 5-aminopyrazoles. Tetrahedron.

[13] Smith C.D., Tchabanenko K., Adlington R.M., Baldwin J.E. (2006). Synthesis of linked heterocycles *via* use of bis-acetylenic compounds. Tetrahedron Lett..

[14] Liu H.L., Jiang H.F., Zhang M., Yao W.J., Zhu Q.H., Tang Z. (2008). One-pot three-component synthesis of pyrazoles through a tandem coupling-cyclocondensation sequence. Tetrahedron Lett..

[15] Wu Y., Chen J.-Y., Ning J., Jiang X., Deng J., Deng Y., Xu R., He W.-M. (2021). Electrochemical multicomponent synthesis of 4-selanylpyrazoles under catalyst-and chemical-oxidant-free conditions. Green Chem..

[16] Bamoniri A., Yaghmaeiyan N., Khaje S. (2023). Synthesis and characterization of highly substituted pyrazoles using silicaphosphoric acid nanoparticles as a recoverable heterogeneous solid acid catalyst. Indian J. Chem. Technol..

[17] Unoh Y., Hirano K., Miura M. (2017). Metal-Free Electrophilic phosphination/cyclization of alkynes. J. Am. Chem. Soc..

[18] Slivka M., Onysko M. (2021). The use of electrophilic cyclization for the preparation of condensed heterocycles. Synthesis.

[19] Zhang Z., Wang S.L., Tan P.P., Gu X.W., Sun W.J., Liu C., Chen J.C., Li J.Z., Sun K. (2022). K_2_S_2_O_8_/I_2_-Promoted Electrophilic Selenylative Cyclization To Access Seleno-Benzo[b]azepines. Org. Lett..

[20] Muzalevskiy V.M., Rulev A.Y., Romanov A.R., Kondrashov E.V., Ushakov I.A., Chertkov A.V., Nenajdenko G. (2017). Selective, Metal-Free Approach to 3- or 5-CF_3_-Pyrazoles: Solvent Switchable Reaction of CF_3_-Ynones with Hydrazines. J. Org. Chem..

[21] Zora M., Kivrak A. (2011). Synthesis of pyrazoles *via* CuI-mediated electrophilic cyclizations of *α, β*-alkynic hydrazones. J. Org. Chem..

[22] Zora M., Kivrak A., Yazici C. (2011). Synthesis of pyrazoles via electrophilic cyclization. J. Org. Chem..

[23] Qian J.Q., Liu Y.K., Zhu J., Jiang B., Xu Z.Y. (2011). A novel synthesis of fluorinated pyrazoles *via* gold (I)-catalyzed tandem aminofluorination of alkynes in the presence of selectfluor. Org. Lett..

[24] Wang Q.D., He L.S., Li K.K., Tsui G.C. (2017). Copper-Mediated Domino Cyclization/Trifluoromethylation/Deprotection with TMSCF_3_: Synthesis of 4-(Trifluoromethyl)pyrazoles. Org. Lett..

[25] Yu X.Z., Shang Y.-Z., Cheng Y.-F., Tian J., Niu Y.L., Gao W.-C. (2020). Synthesis of 4-chalcogenyl pyrazoles *via* electrophilic chalcogenation/cyclization of *α,β*-alkynic hydrazones. Org. Biomol. Chem..

[26] Yao H.-F., Li F.-H., Li J., Wang S.-Y., Ji S.-J. (2020). Iron(III) chloride-promoted cyclization of *α,β*-alkynic tosylhydrazones with diselenides: Synthesis of 4-(arylselanyl)-1*H*-pyrazoles. Org. Biomol. Chem..

[27] Cao Z., Zhu Q., Lin Y.-W., He W.-M. (2019). The concept of dual roles design in clean organic preparation. Chin. Chem. Lett..

[28] Lu L.-H., Wang Z., Xia W., Cheng P., Zhang B., Cao Z., He W.-M. (2019). Sustainable routes for quantitative green selenocyanation of activated alkynes. Chin. Chem. Lett..

[29] Zheng H., Cao X.-T., Du K., Xu J., Zhang P.F. (2014). A highly efficient way to capture CX_2_ (O, S) mildly in reusable ReILs at atmospheric pressure. Green Chem..

[30] Taheri A., Lai B., Cheng C., Gu Y. (2015). Brønsted acid ionic liquid-catalyzed reductive Friedel–Crafts alkylation of indoles and cyclic ketones without using an external reductant. Green Chem..

[31] Liu F.S., Ping R., Gu Y.Q., Zhao P.H., Liu B., Gao J., Liu M.S. (2020). Efficient one pot capture and conversion of CO_2_ into quinazoline-2,4(1*H*, 3*H*)-diones using triazolium-based ionic liquids. ACS Sustain. Chem. Eng..

