# Supplementary material for: Temperature-Controlled Divergent Synthesis of Pyrazoles and 1-Tosyl-1H-pyrazoles under Transition-Metal-Catalyst- and Oxidant-Free Conditions
Source: Molecules. 2024 Apr 10;29(8):1706. doi: 10.3390/molecules29081706 (PMC11052136; doi:10.3390/molecules29081706)

Supporting Information

Temperature-controlled divergent synthesis of pyrazoles and 1-tosyl-1*H*-pyrazoles under transition-metal-catalyst and oxidant-free conditions

Kai Wang,[1] Wenjing Xu,[1] Chengcai Xia [2,*] and Xian-Ting Cao[1,*]

1. College of Medical Engineering & the Key Laboratory for Medical Functional Nanomaterials, Jining Medical University, Jining, 272067, China.
2. Institute of Pharmacology, Pharmacy College, Shandong First Medical University & Shandong Academy of Medical Sciences, Jinan 271016, China. Emails:

E-mail address: chemcxt@mail.jnmc.edu.cn; xiachc@163.com.

Table of Contents

1. Reaction progress diagram of 2a................ 2
2. Optimization of reaction conditions...3
3. Yields of recyclability test..3
4. ^{1}H and ^{13}C NMR spectra of the products..4

1. Reaction progress diagram of 2a.

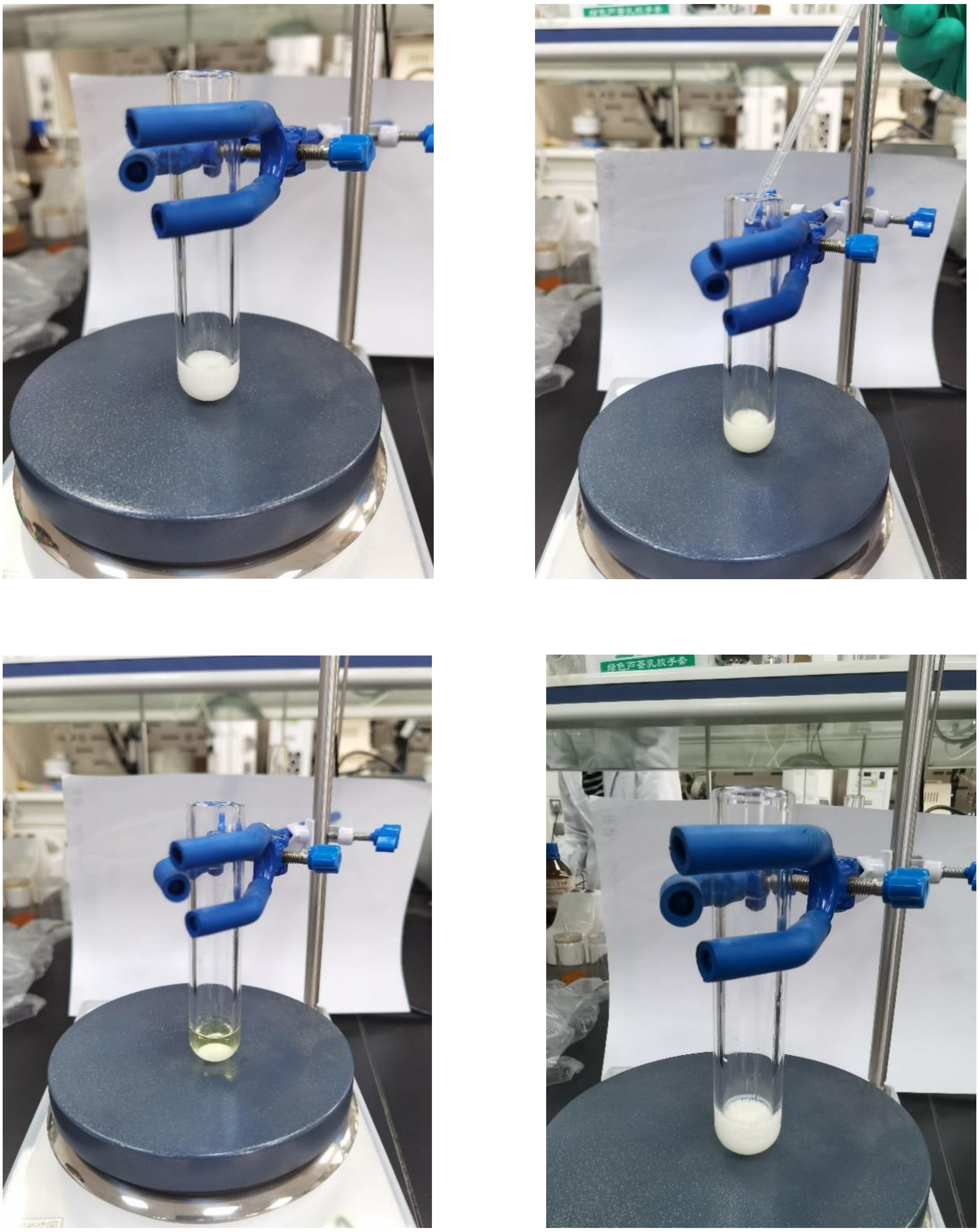

2. Optimization of Reaction Conditions [a,b]

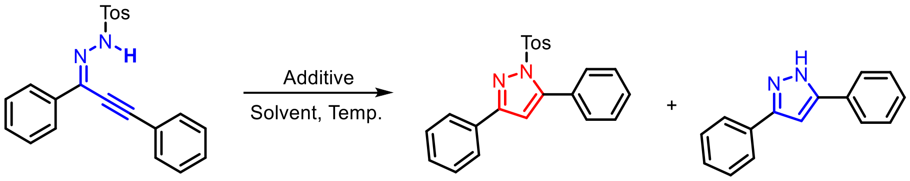

1a 2a 3a

Entry	Solvent	Yield of 2a [%] [b]	Yield of 3a [%] [b]
1	CH_3CN	N.R.	N.R.
2	DMSO	N.R.	N.R.
3	THF	N.R.	N.R.
4	AcOH	N.R.	N.R.
5	EtOH	N.R. (95)[d]	N.R. (65)[e]
6	EtOH	73 [f]	38 [g]
7	EtOH	47[h] (97)[i]	28[h] (68)[i]
8	[HDBU][OAc]	98	85[c](13)[j]
9	[HDBU][NHS]	95	78[c]
10	[HTMG][NHS]	78	63[c]
11	[HDBU][OAc][NHS]	86	68[c]
12	[HTMG][HDBU][OAc][NHS]	80	58[c]

[a] Reaction conditions: **1a** (0.2 mmol), solvent (2.0 mL), r.t., 0.5 h; [b] Isolated yields. [c] 95 °C, 12 h. [d] DBU (1.0 equiv.) additive, r.t., 0.5 h. [e] DBU (1.0 equiv.) additive, 95 °C, 12 h. [f] DBU (1.0 equiv.) additive, 50 °C., 0.5 h. [g] DBU (1.0 equiv.) additive, 50 °C., 12 h. [h] DBU (0.5 equiv.) additive, r.t., 0.5 h. [i] DBU (2.0 equiv.) additive, 95 °C, 12 h. [j] 40 °C, 12 h. [HDBU] = 2,3,4,6,7,8,9,10-octahydropyrimido[1,2-*a*]azepin-1-ium, [NHS] = 2,5-dioxopyrrolidin-1-olate, [HTMG] = bis(dimethylamino)-methaniminium.

3. Yields of recyclability test.

Run / Yield (%)	1	2	3	4	5
**2a**	95	93	92	88	84
**3a**	85	82	77	71	72

4. ¹H and ¹³C NMR spectra of the products.

Figure S1 ¹H NMR Spectra of Compound of 2a

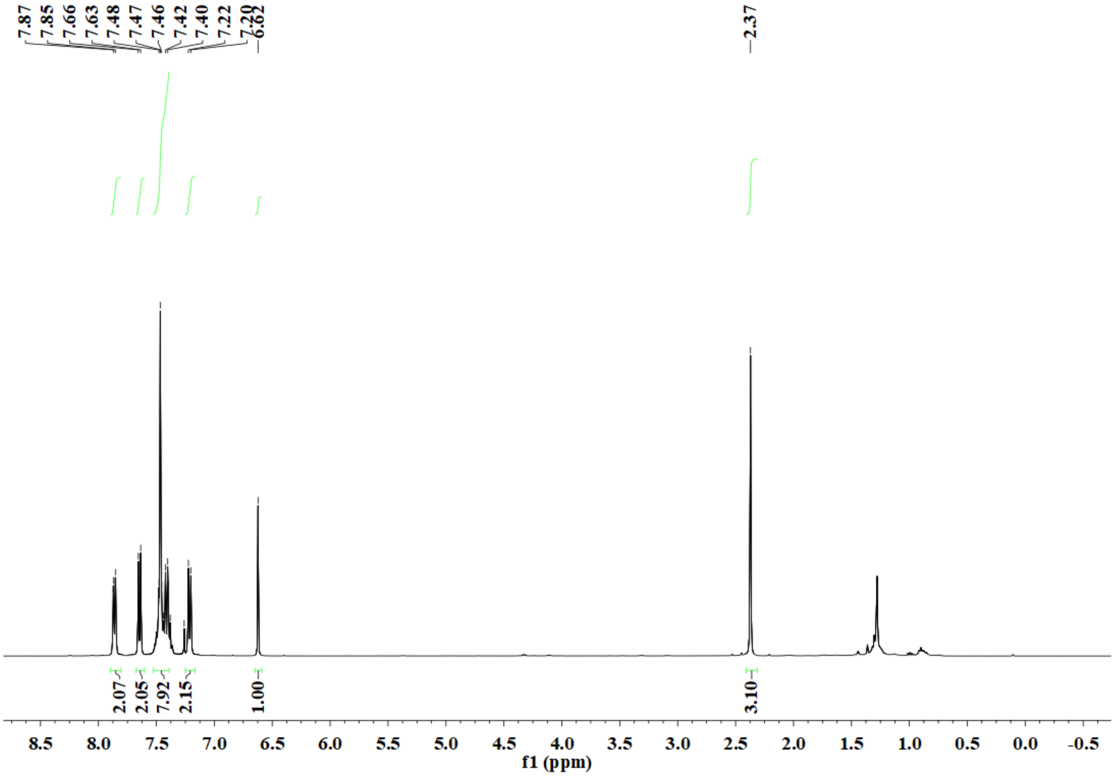

Figure S2 ¹³C NMR Spectra of Compound of 2a

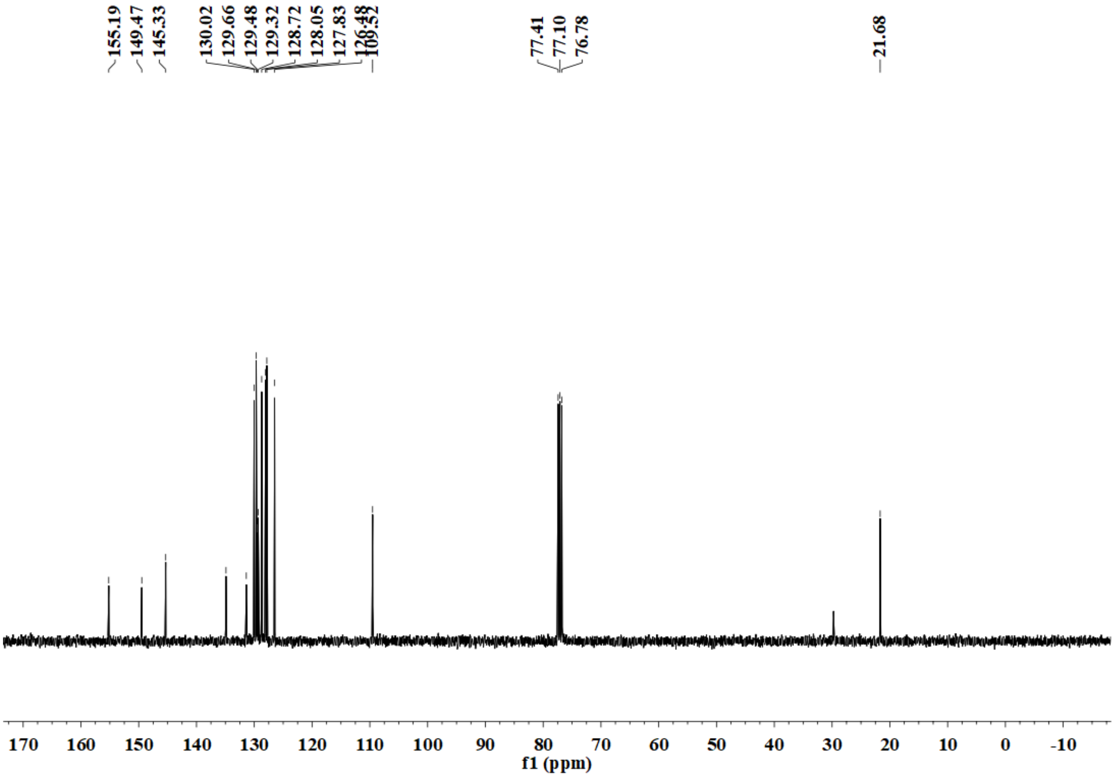

Figure S3 ^{1}H NMR Spectra of Compound of 2b

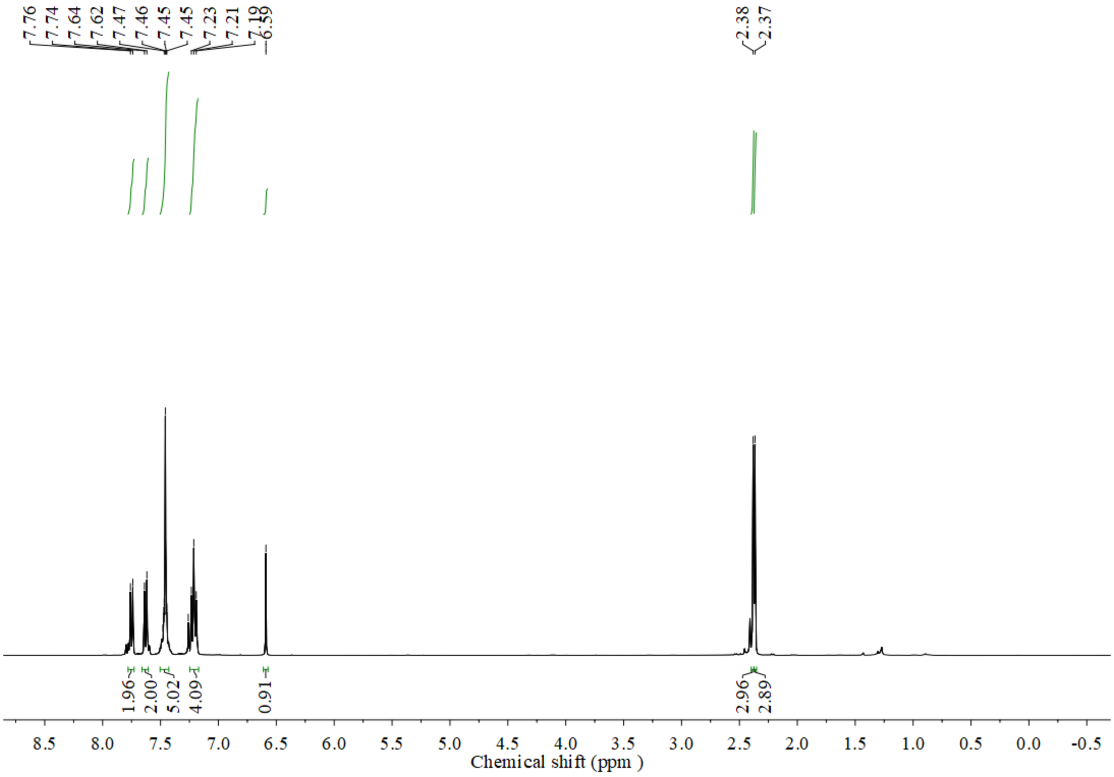

Figure S4 ^{13}C NMR Spectra of Compound of 2b

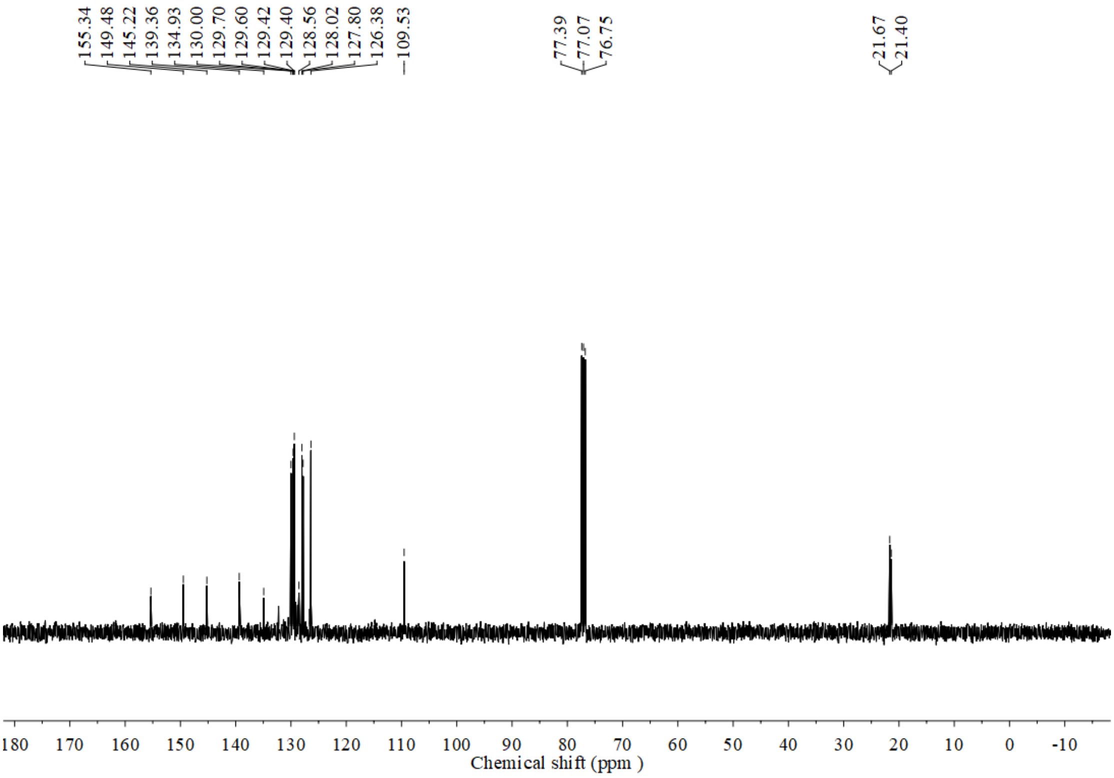

Figure S5 ^{1}H NMR Spectra of Compound of 2c

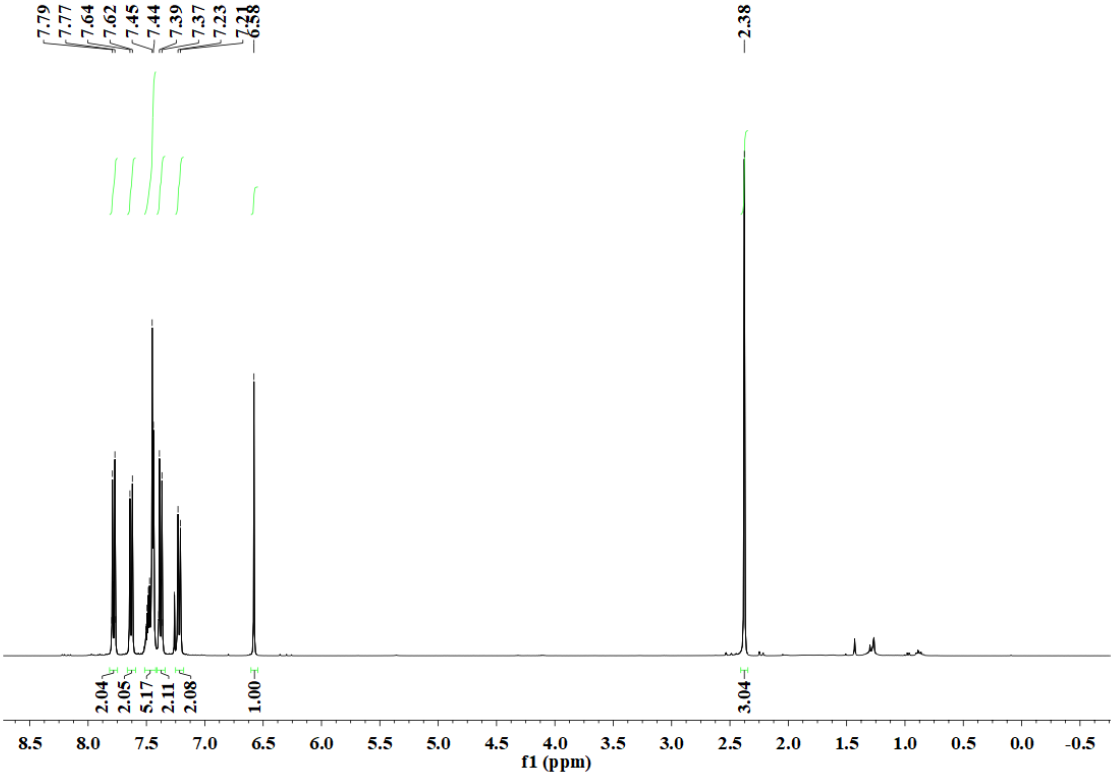

Figure S6 ^{13}C NMR Spectra of Compound of 2c

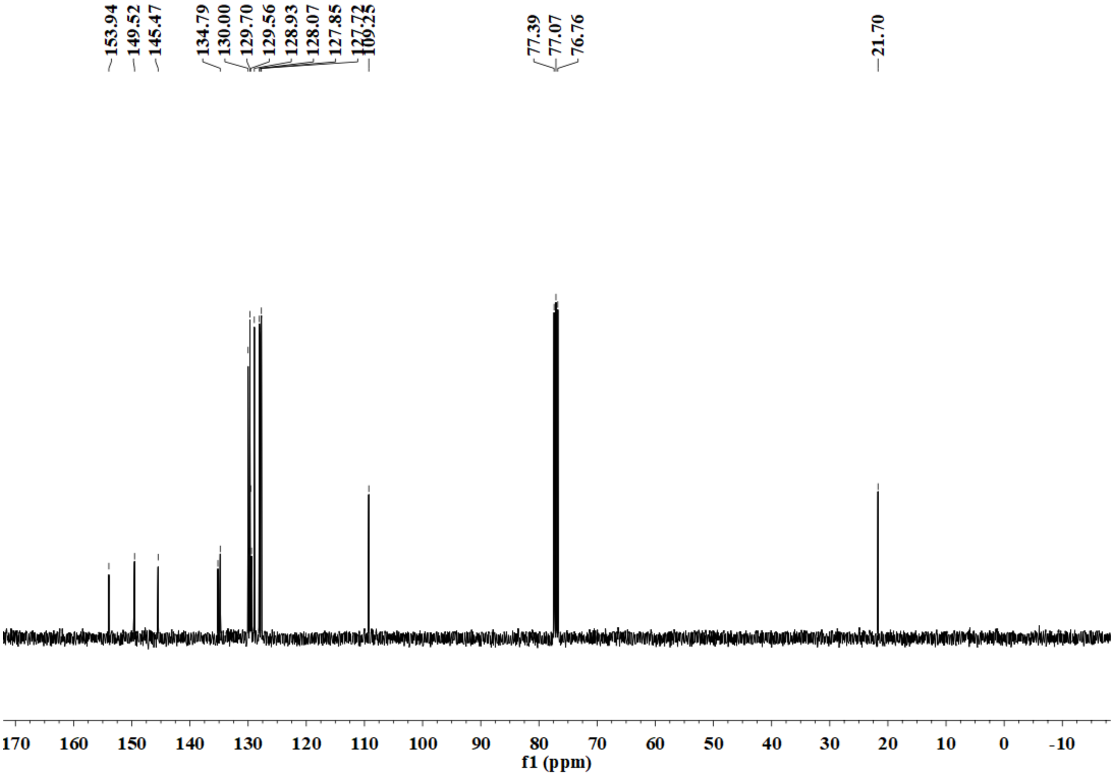

Figure S7 ^{1}H NMR Spectra of Compound of 2d

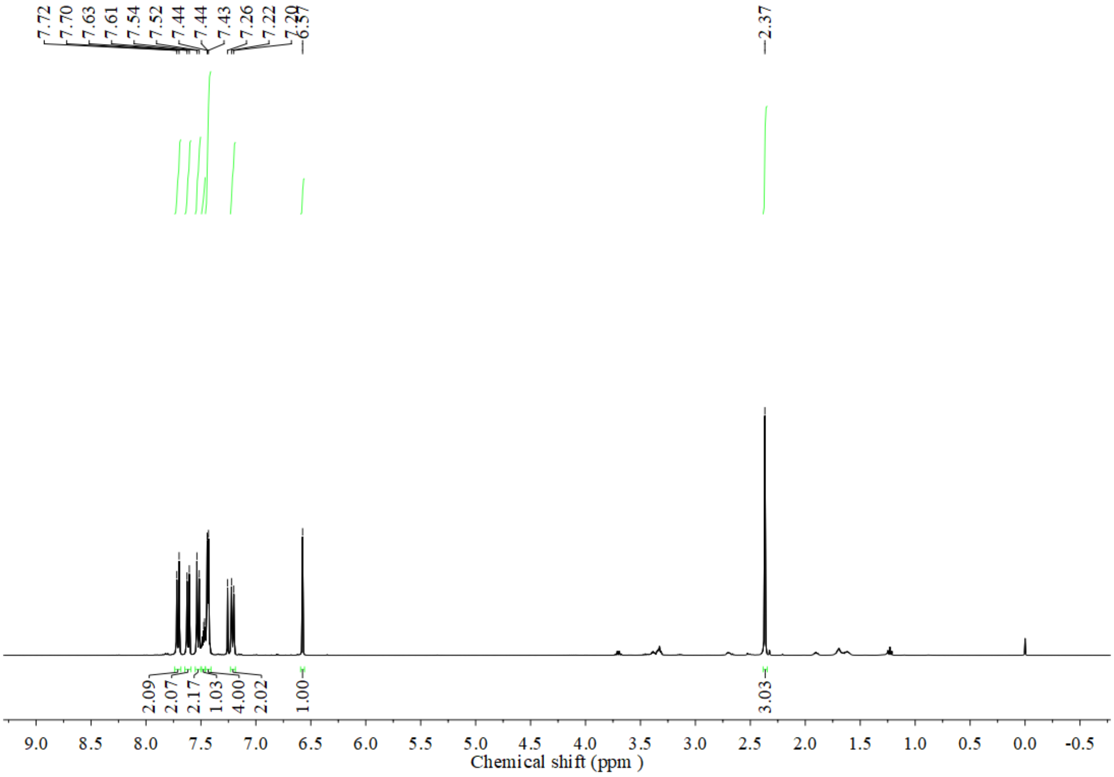

Figure S8 ^{13}C NMR Spectra of Compound of 2d

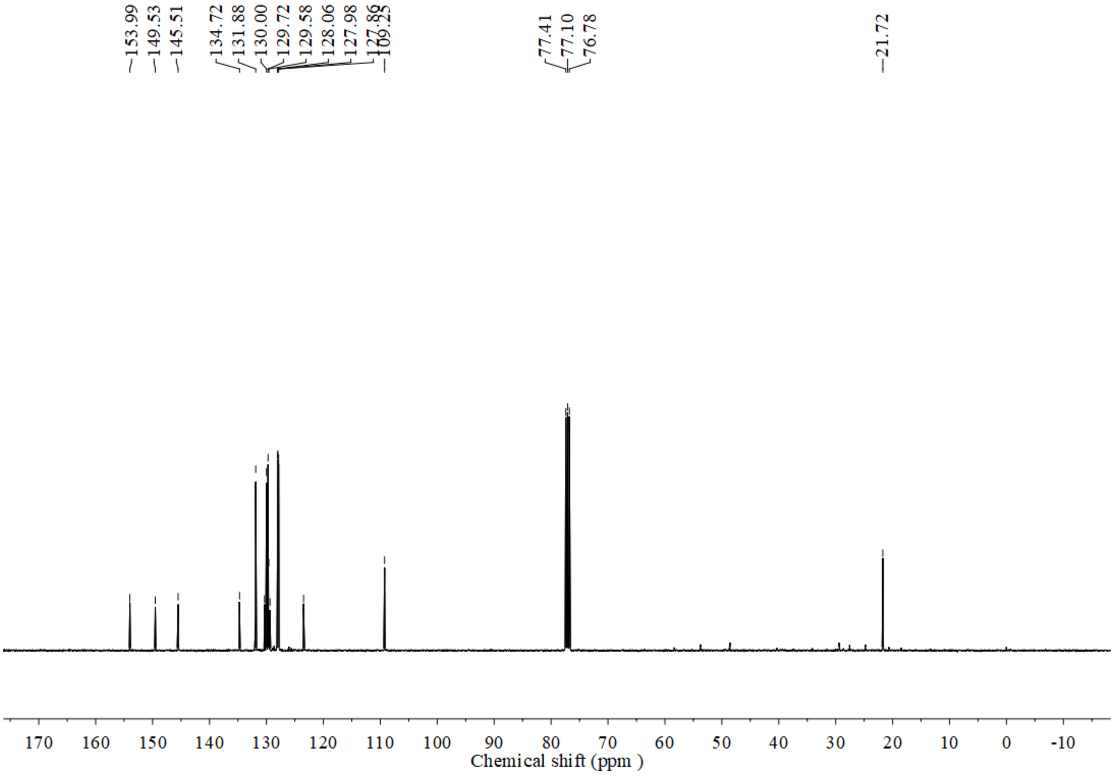

Figure S9 ^{1}H NMR Spectra of Compound of 2e

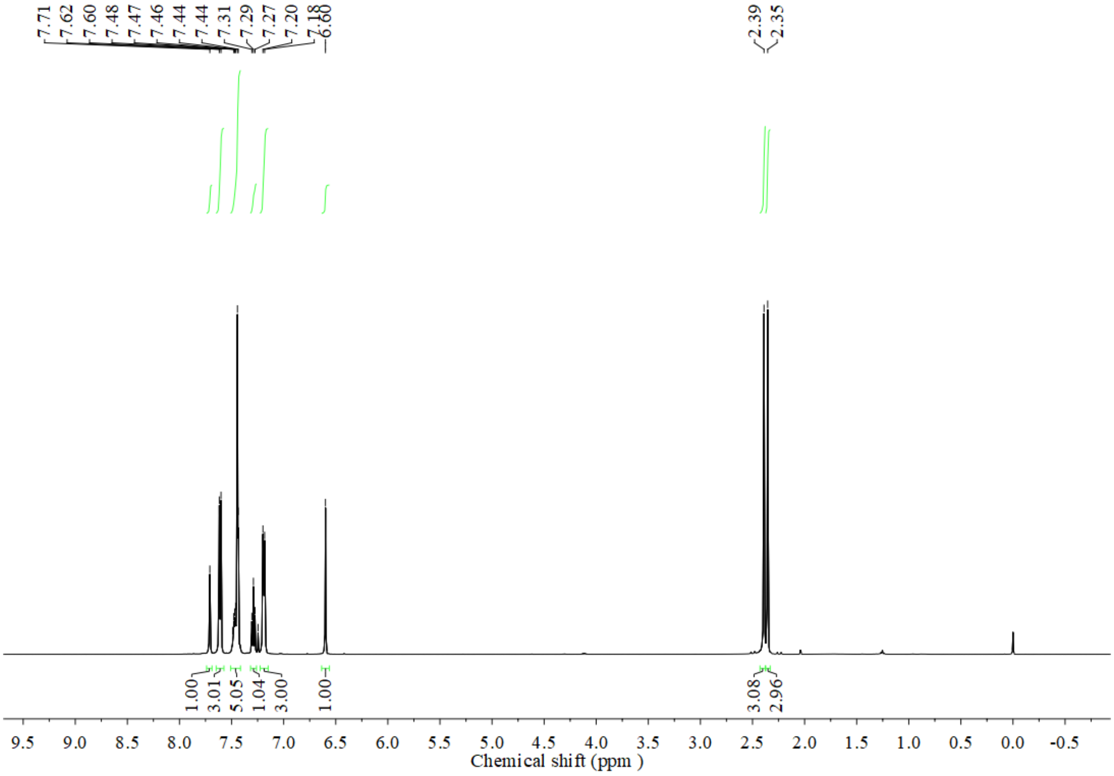

Figure S10 ^{13}C NMR Spectra of Compound of 2e

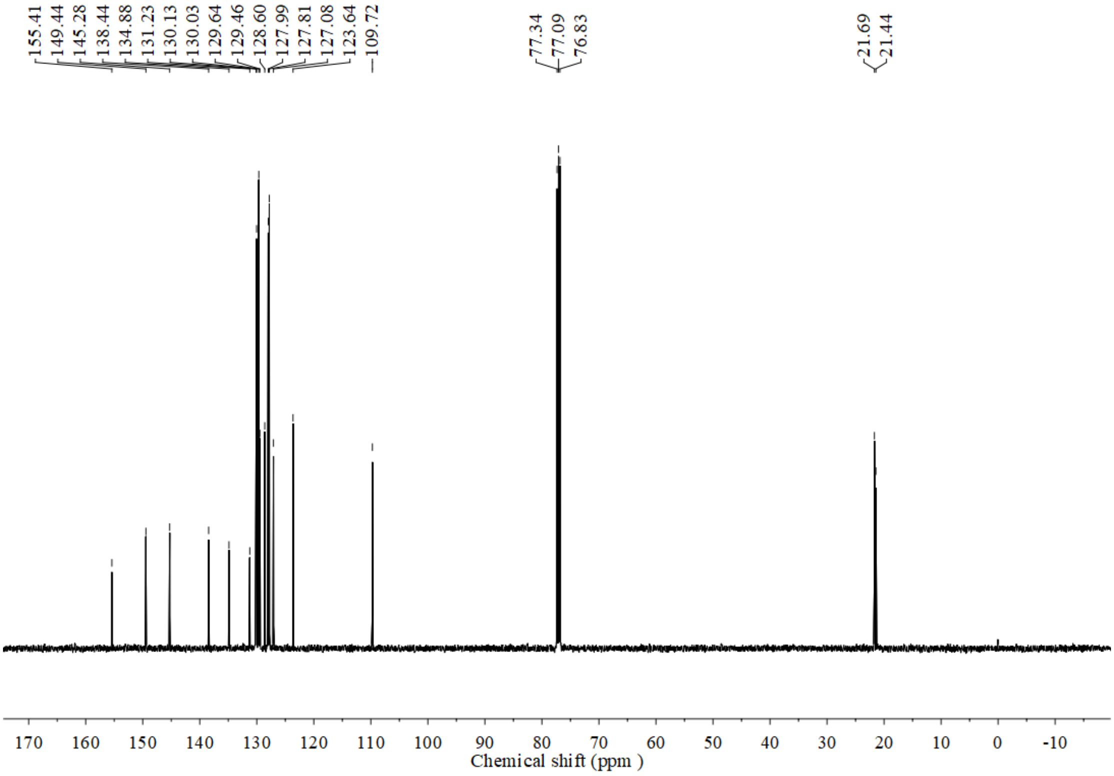

Figure S11 ^{1}H NMR Spectra of Compound of 2f

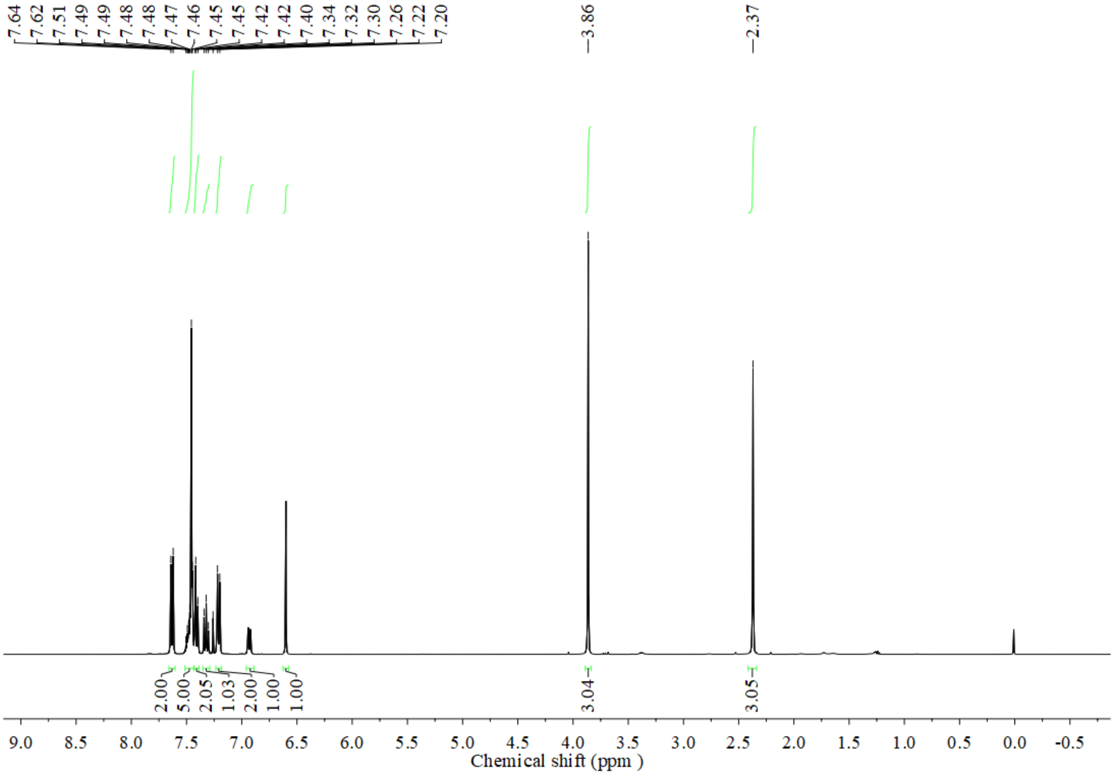

Figure S12 ^{13}C NMR Spectra of Compound of 2f

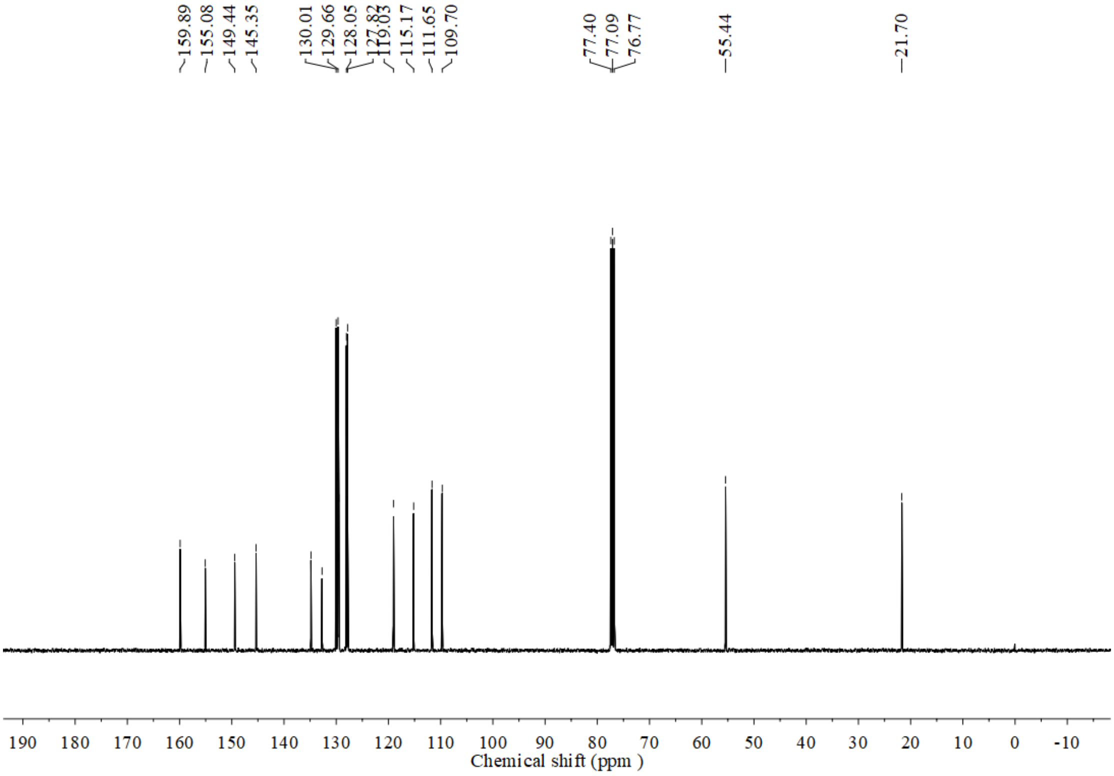

Figure S13 ^{1}H NMR Spectra of Compound of 2g

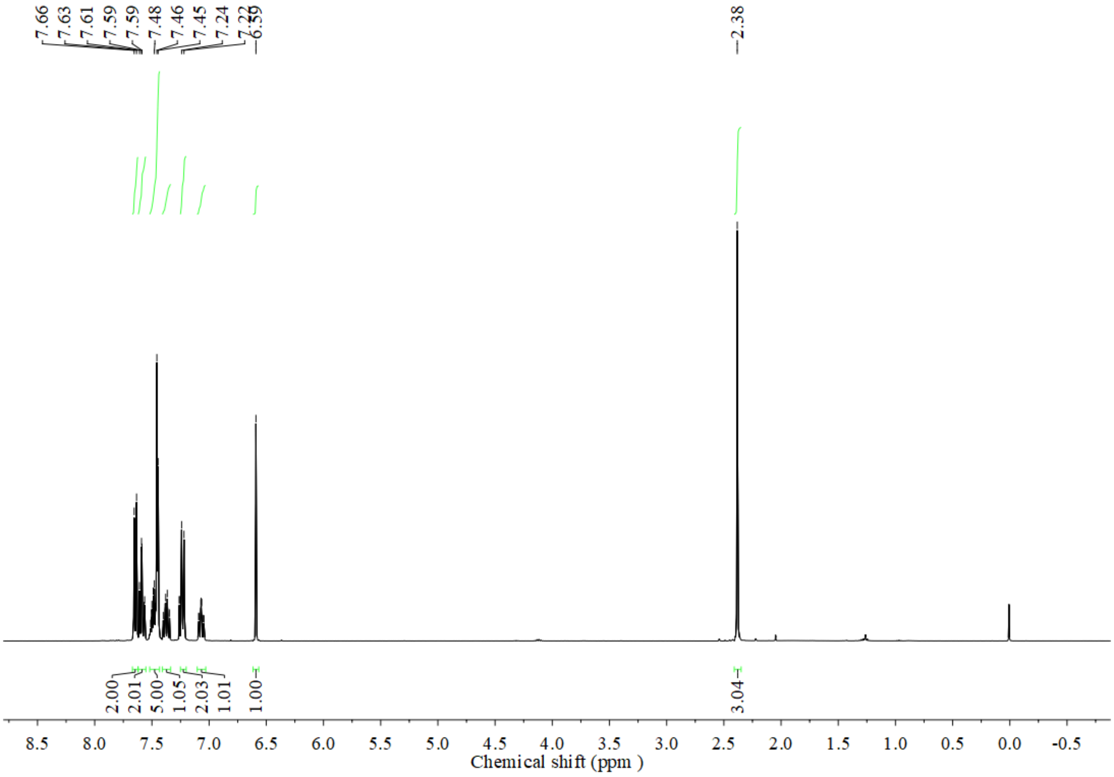

Figure S14 ^{13}C NMR Spectra of Compound of 2g

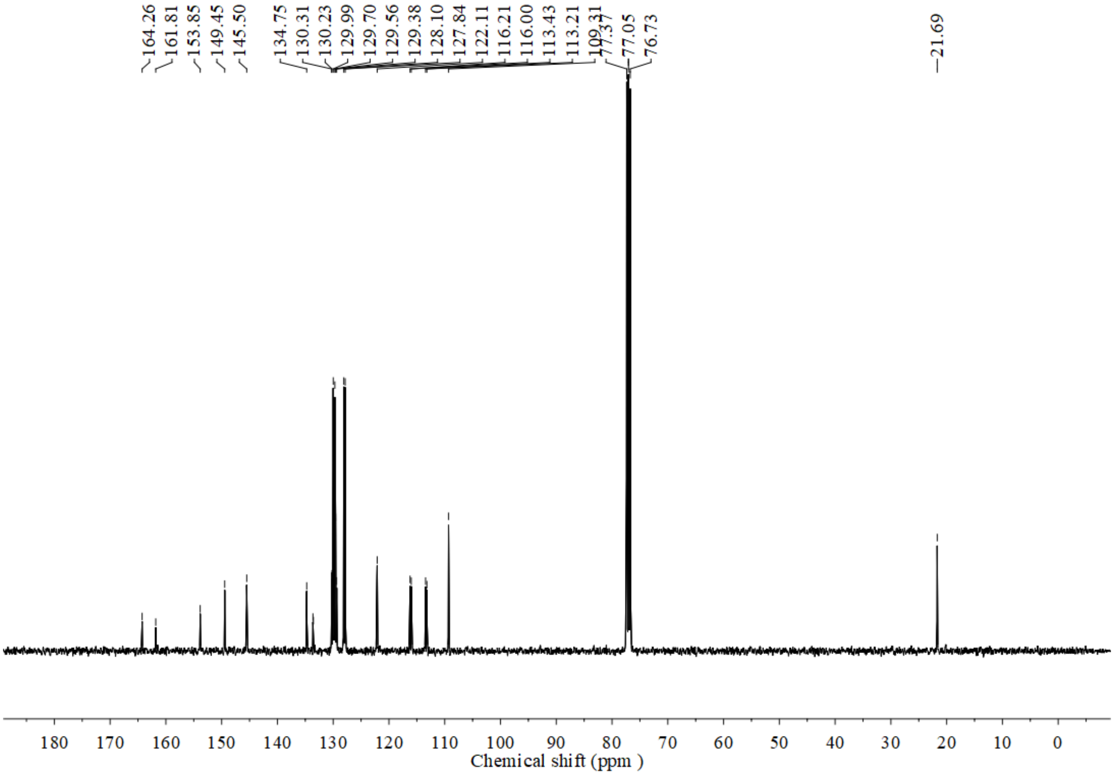

Figure S15 ^{1}H NMR Spectra of Compound of 2h

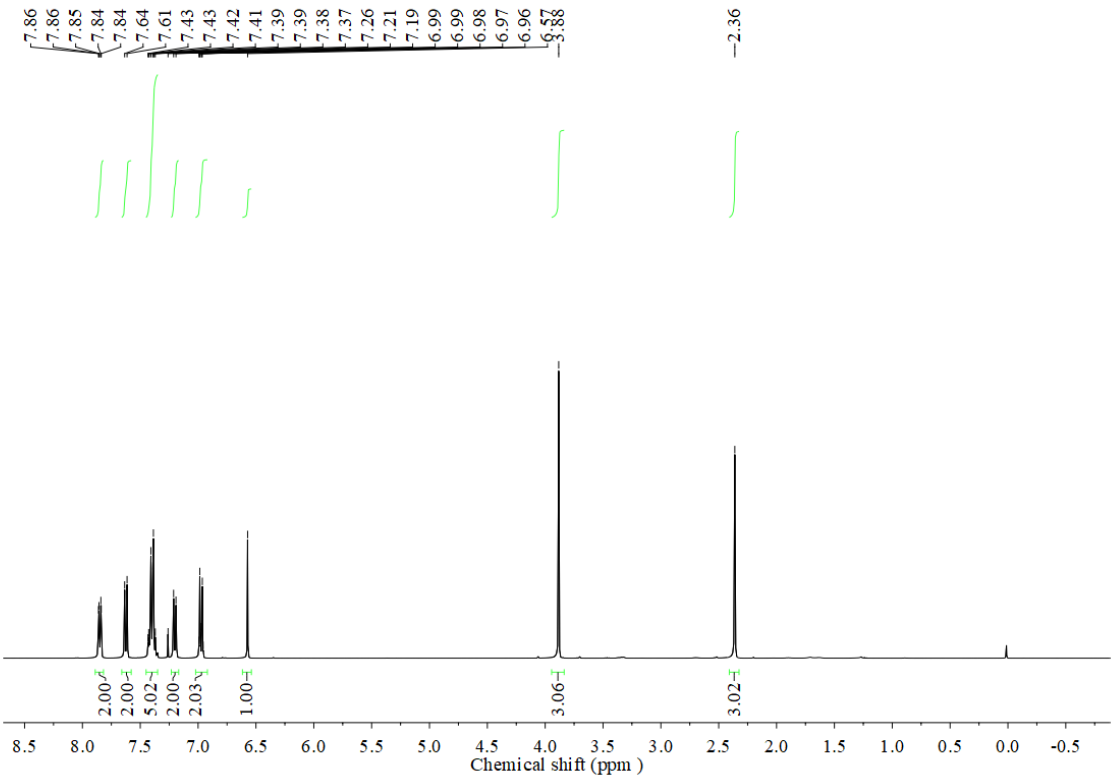

Figure S16 ^{13}C NMR Spectra of Compound of 2h

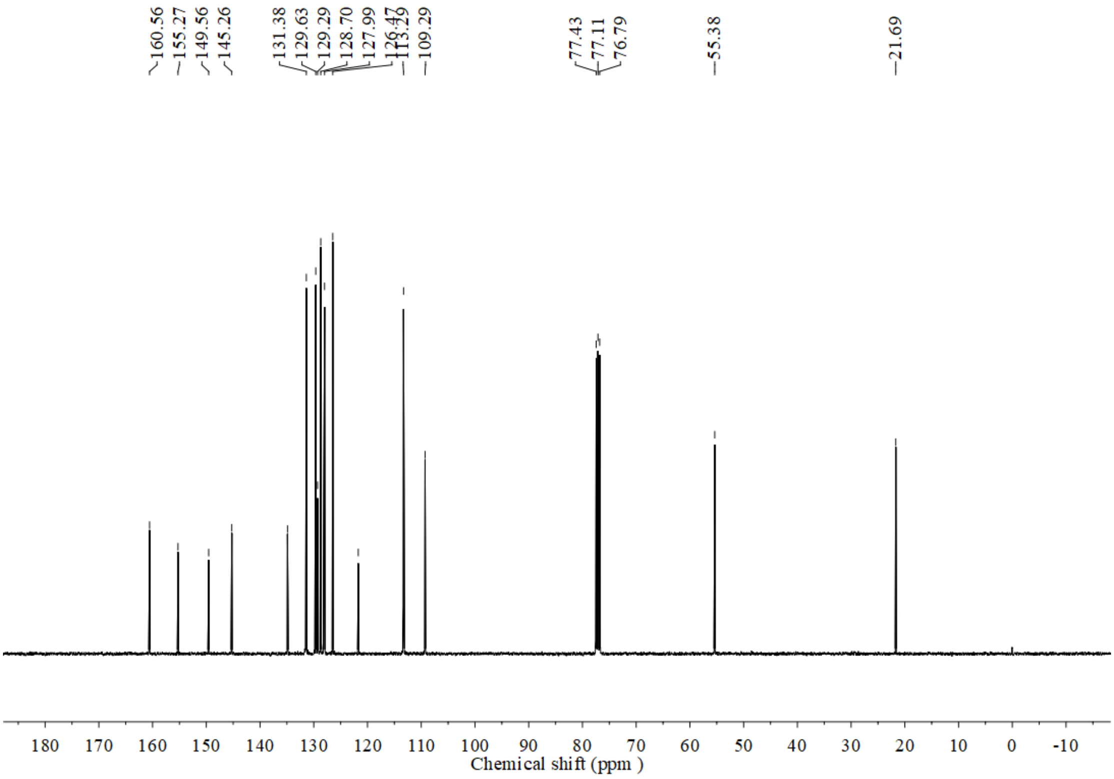

Figure S17 ^{1}H NMR Spectra of Compound of 2i

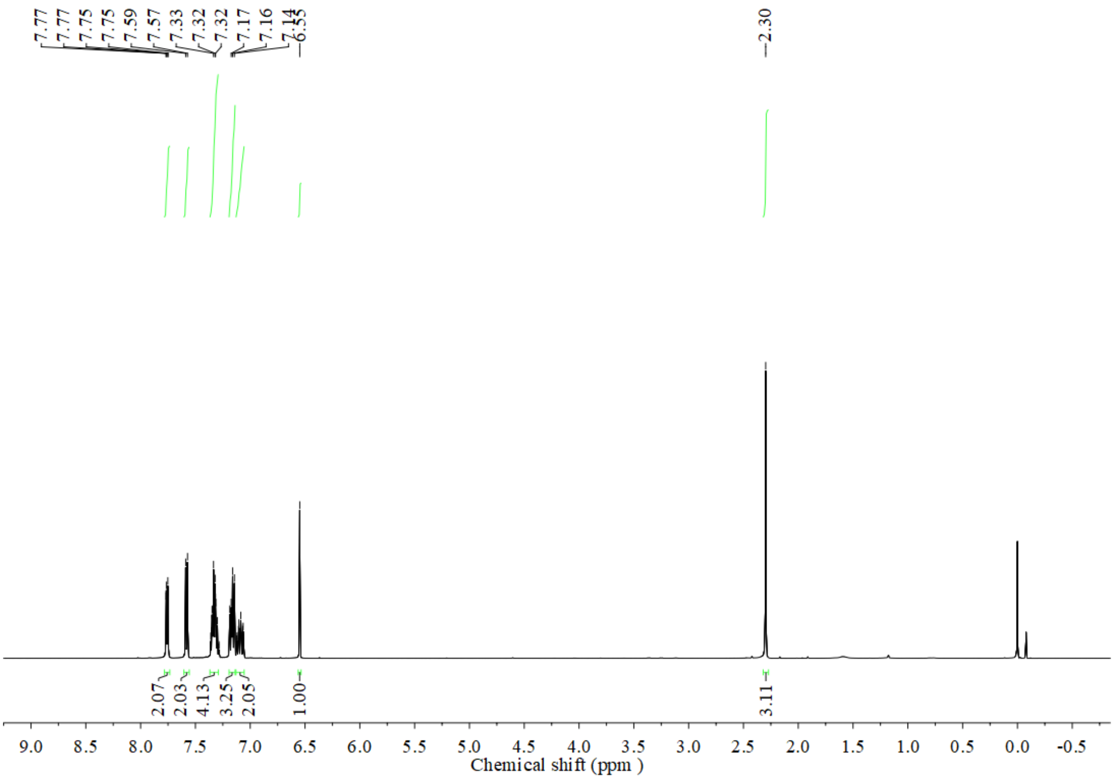

Figure S18 ^{13}C NMR Spectra of Compound of 2i

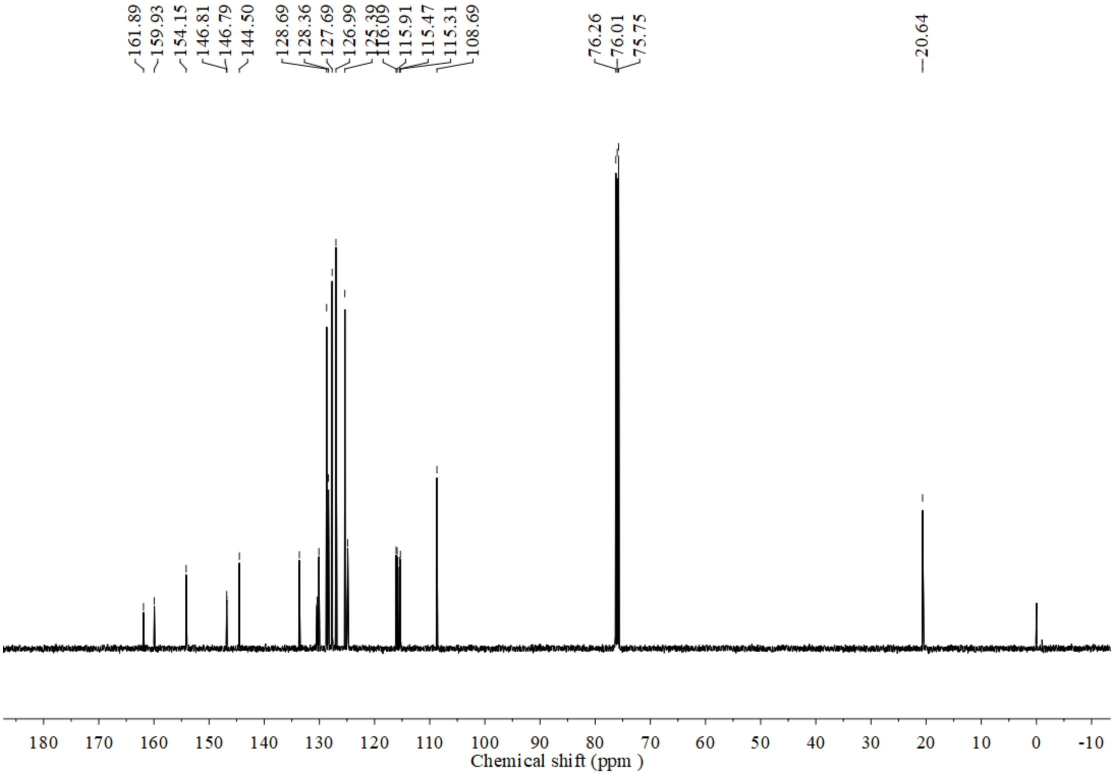

Figure S19 ^{1}H NMR Spectra of Compound of 2j

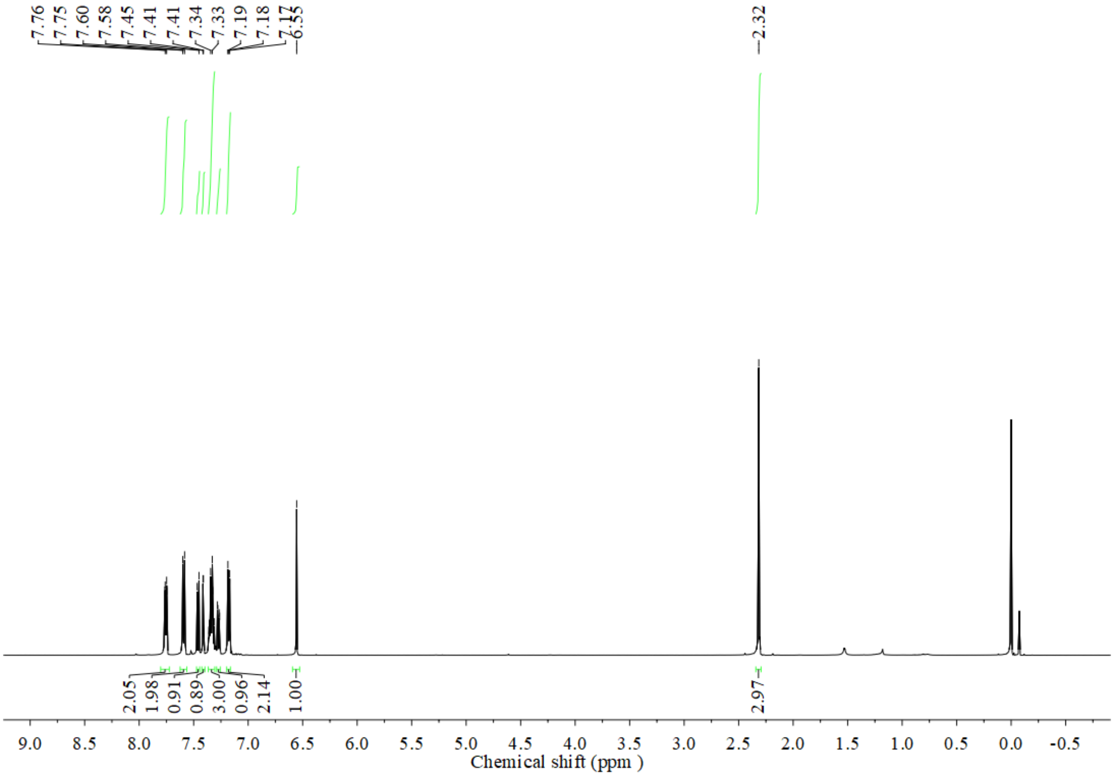

Figure S20 ^{13}C NMR Spectra of Compound of 2j

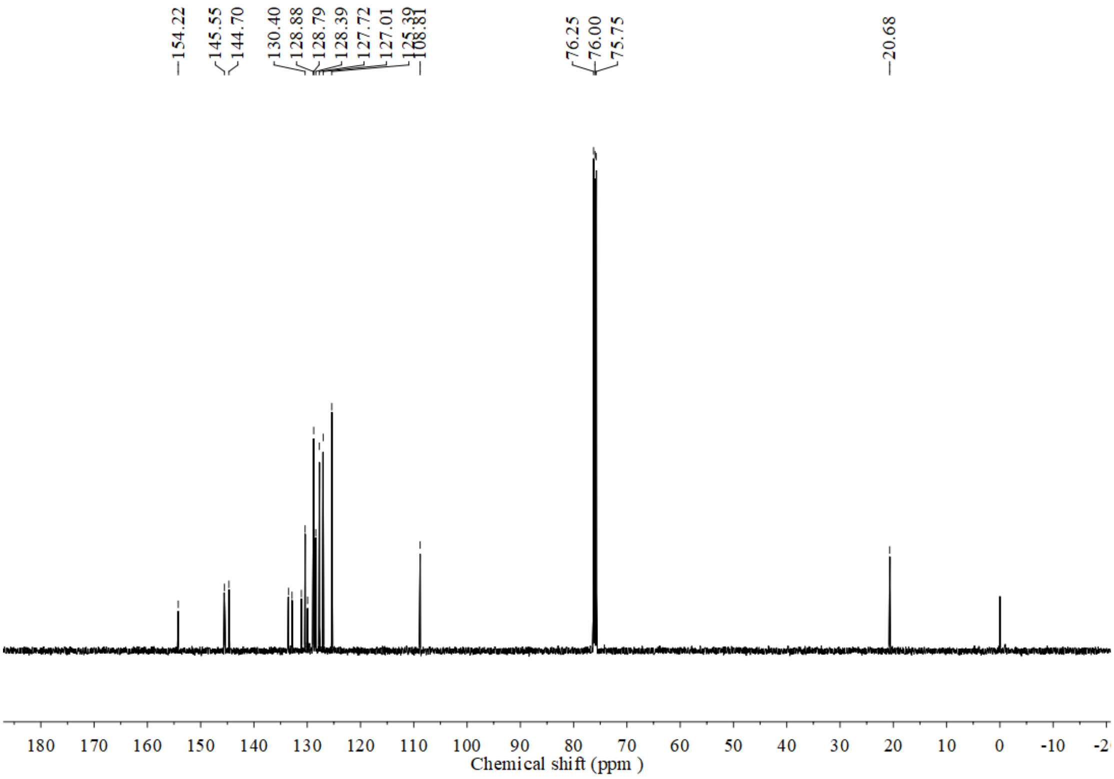

Figure S21 ^{1}H NMR Spectra of Compound of 2k

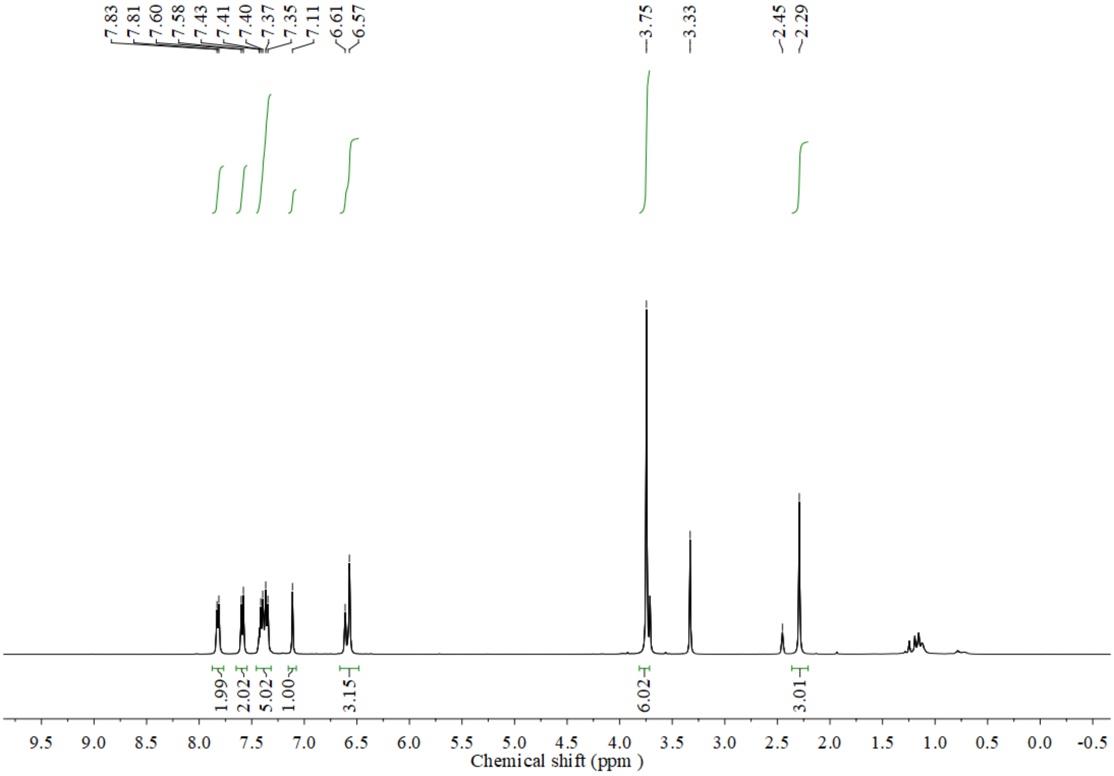

Figure S22 ^{13}C NMR Spectra of Compound of 2k

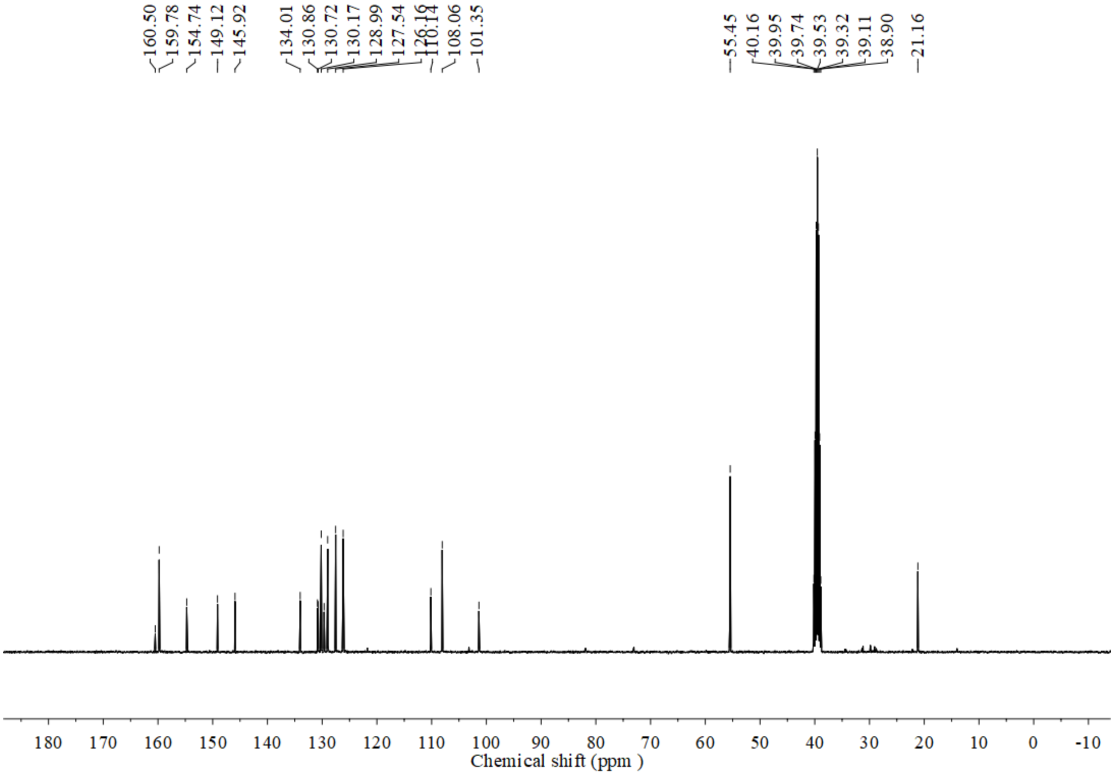

Figure S23 ^{1}H NMR Spectra of Compound of 2l

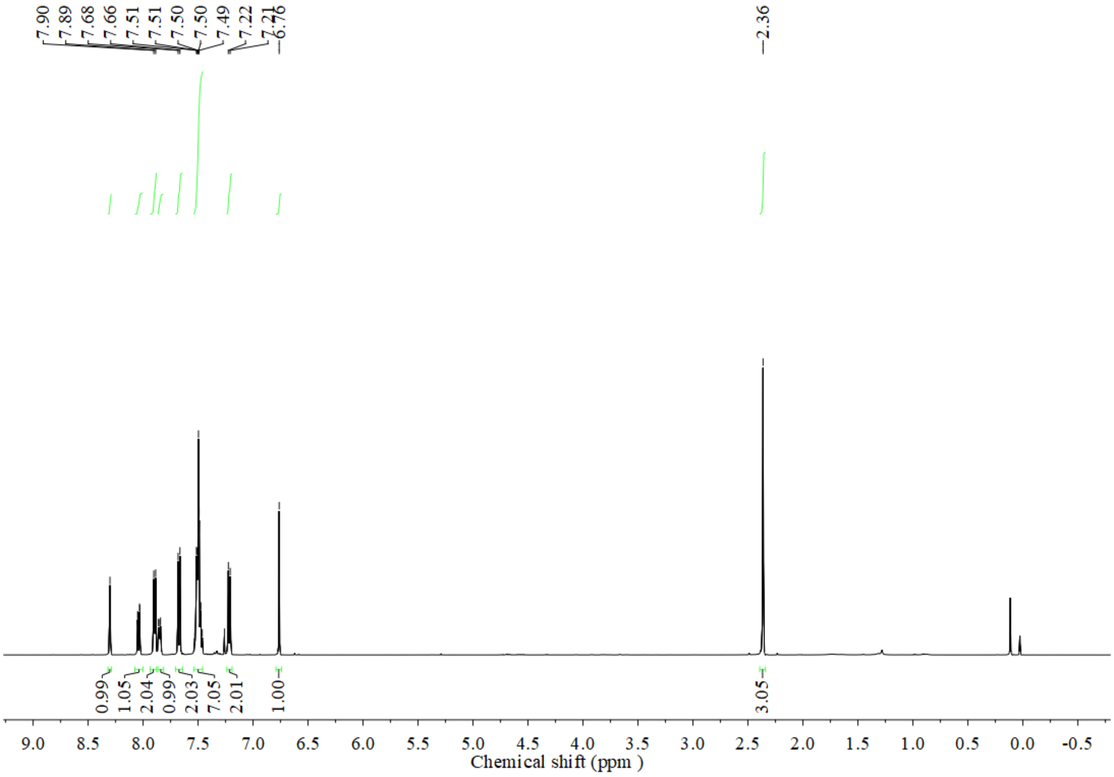

Figure S24 ^{13}C NMR Spectra of Compound of 2l

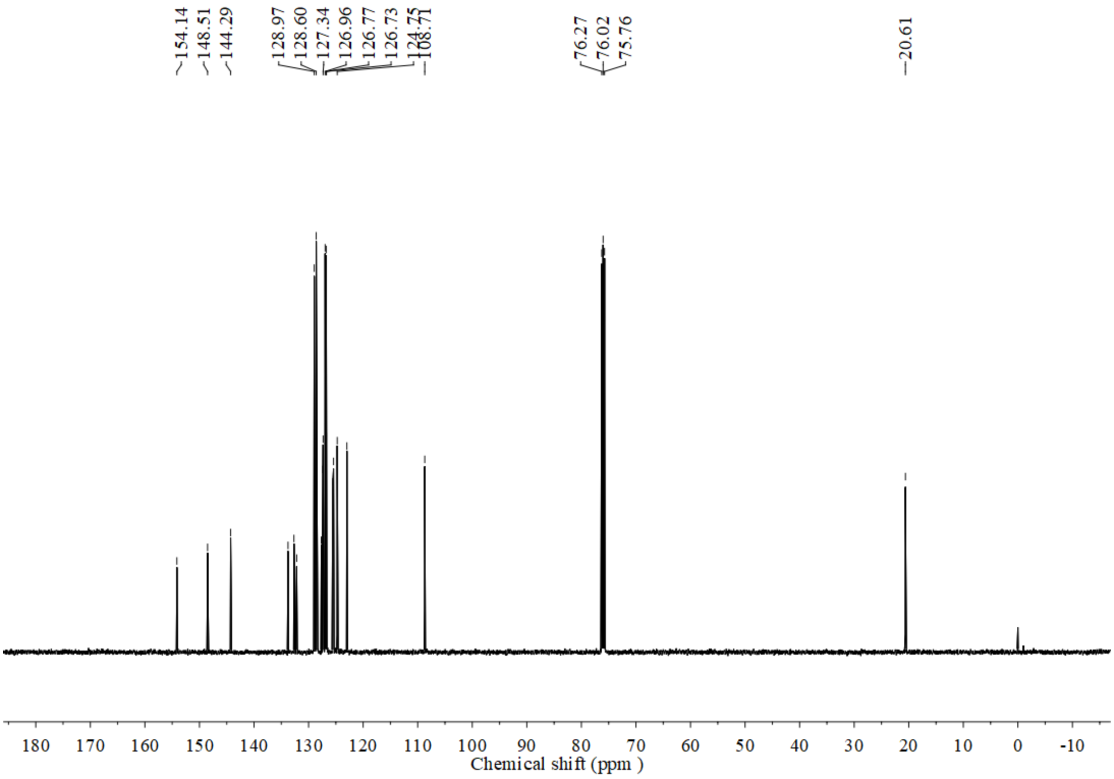

Figure S25 ^{1}H NMR Spectra of Compound of 2m

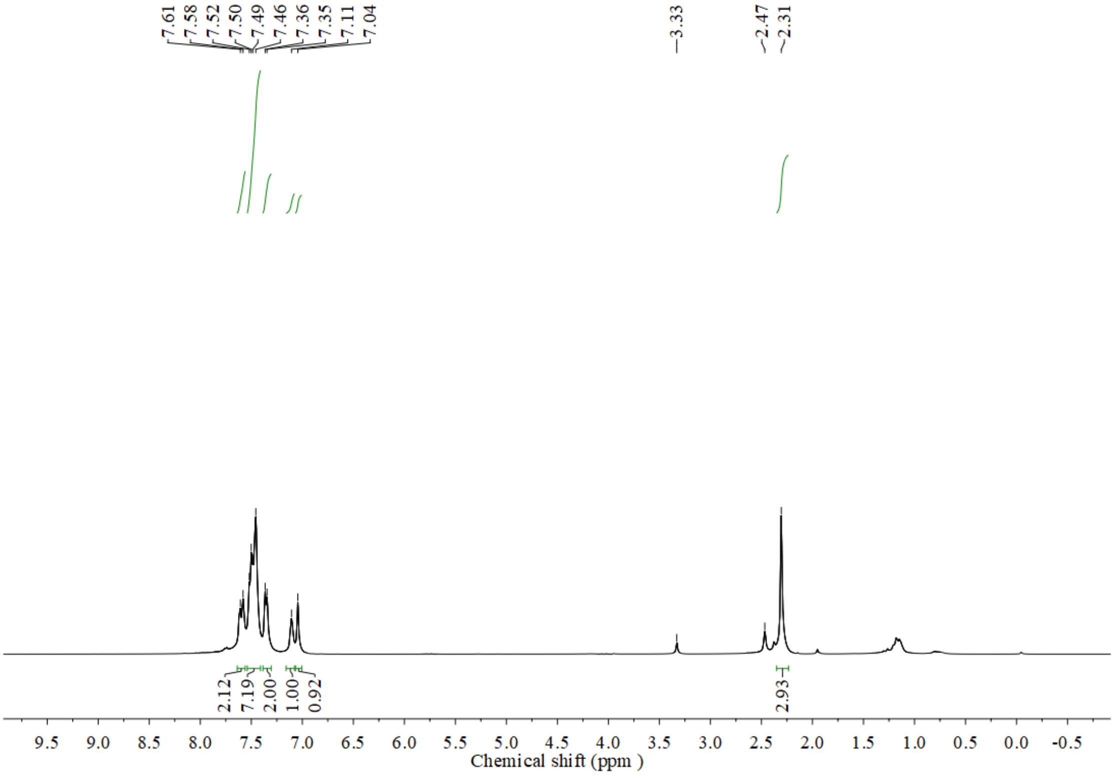

Figure S26 ^{13}C NMR Spectra of Compound of 2m

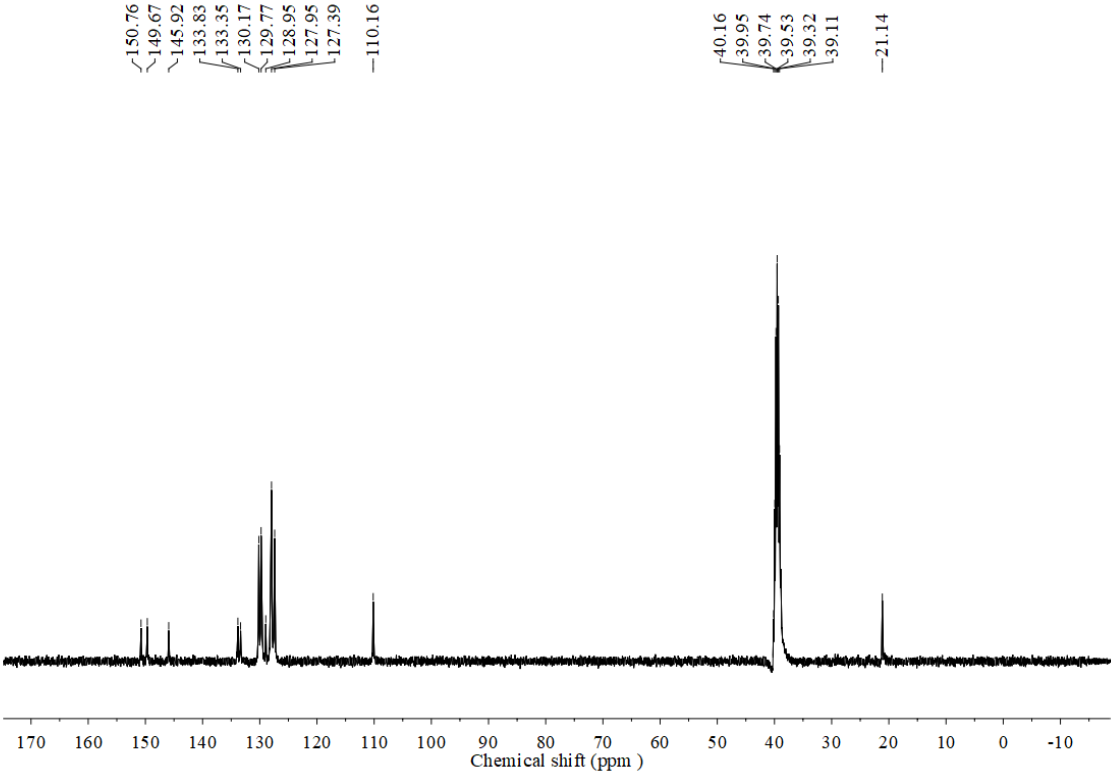

Figure S27 ^{1}H NMR Spectra of Compound of 2n

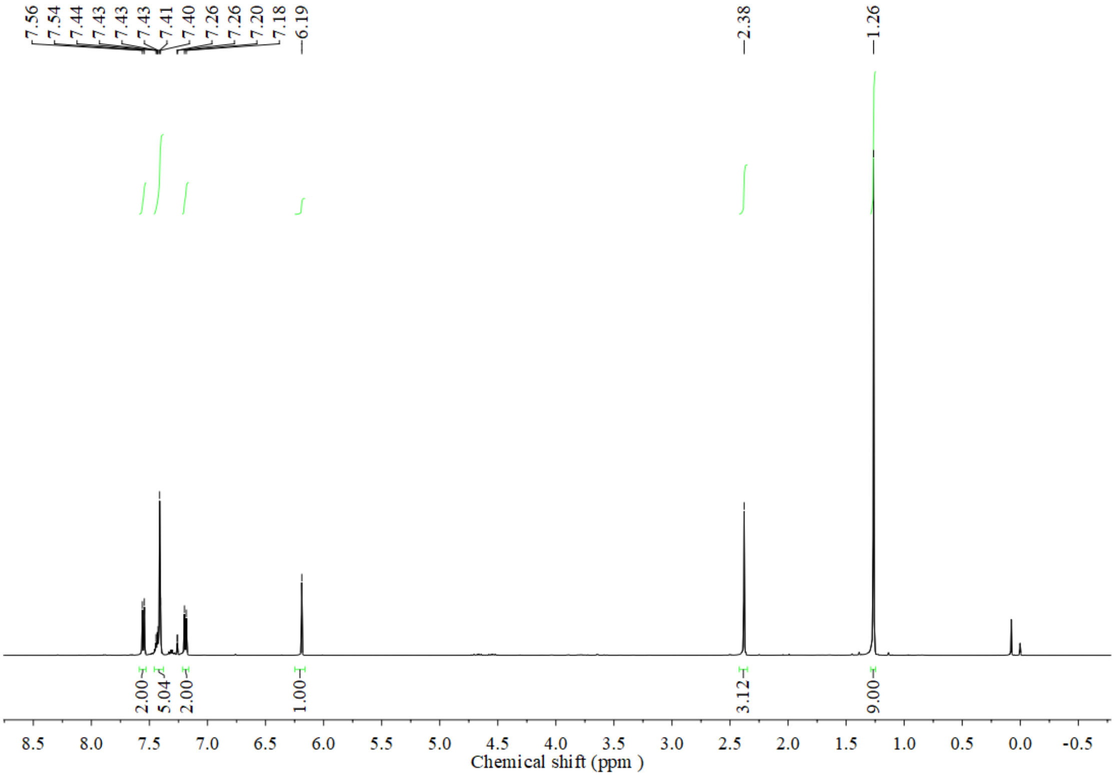

Figure S28 ^{13}C NMR Spectra of Compound of 2n

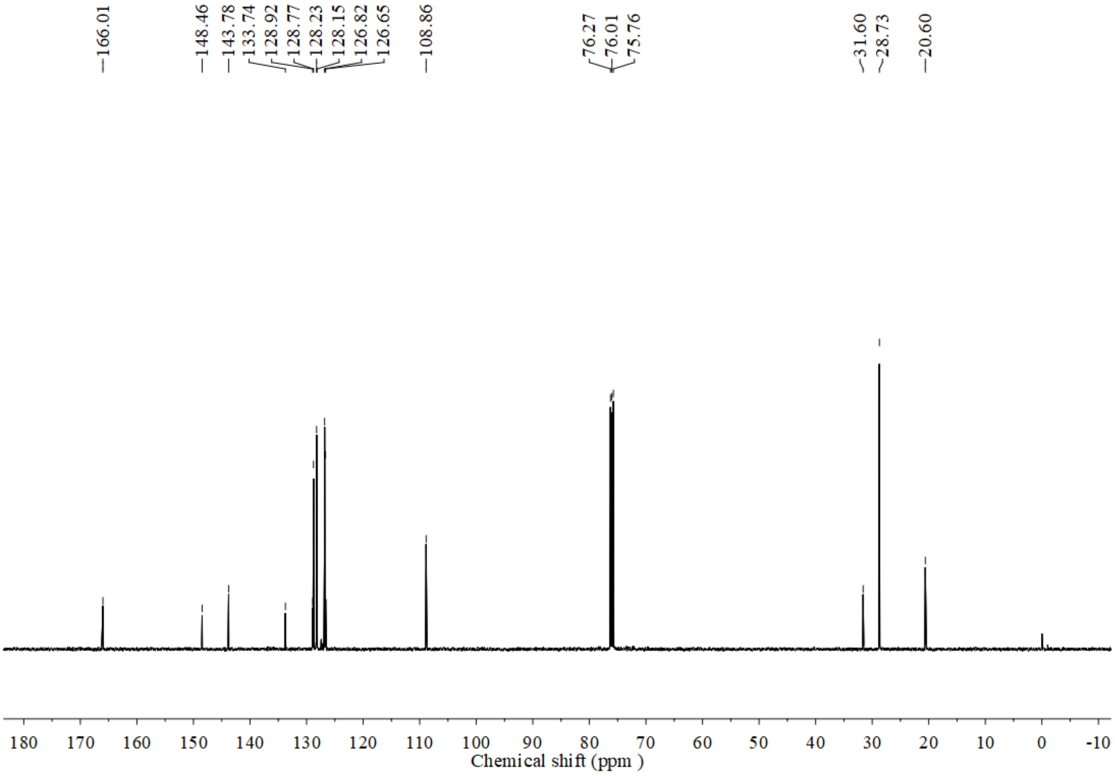

Figure S29 ^{1}H NMR Spectra of Compound of 2o

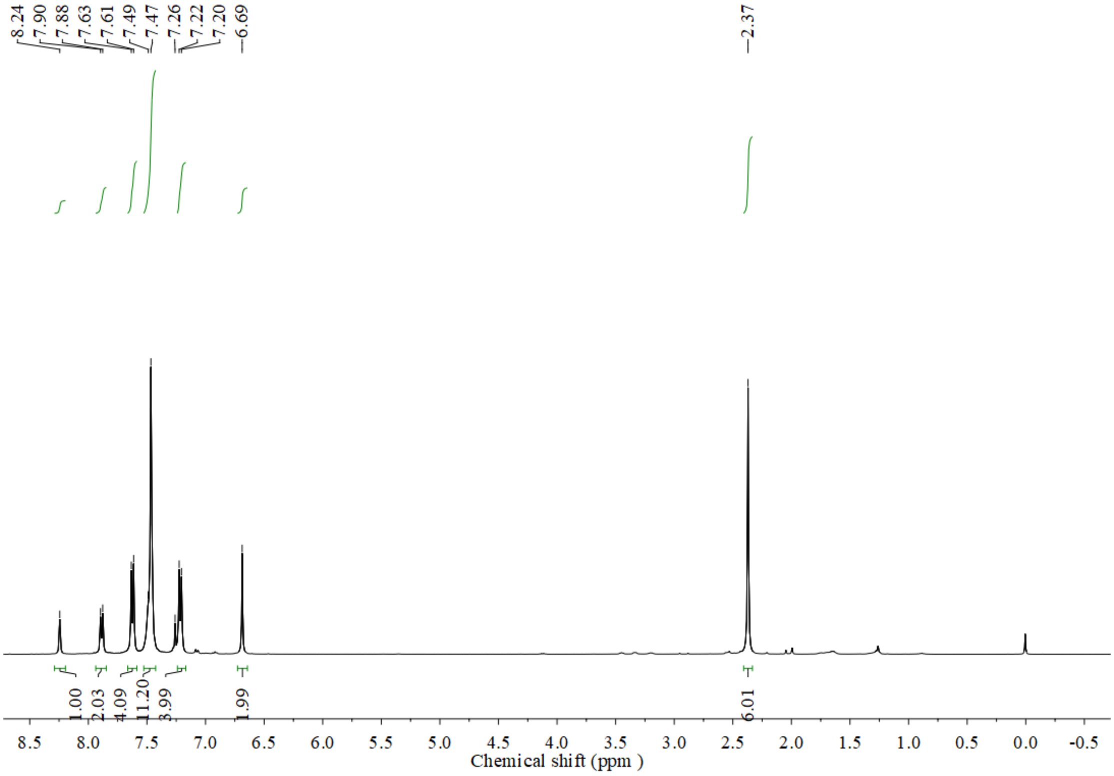

Figure S30 ^{13}C NMR Spectra of Compound of 2o

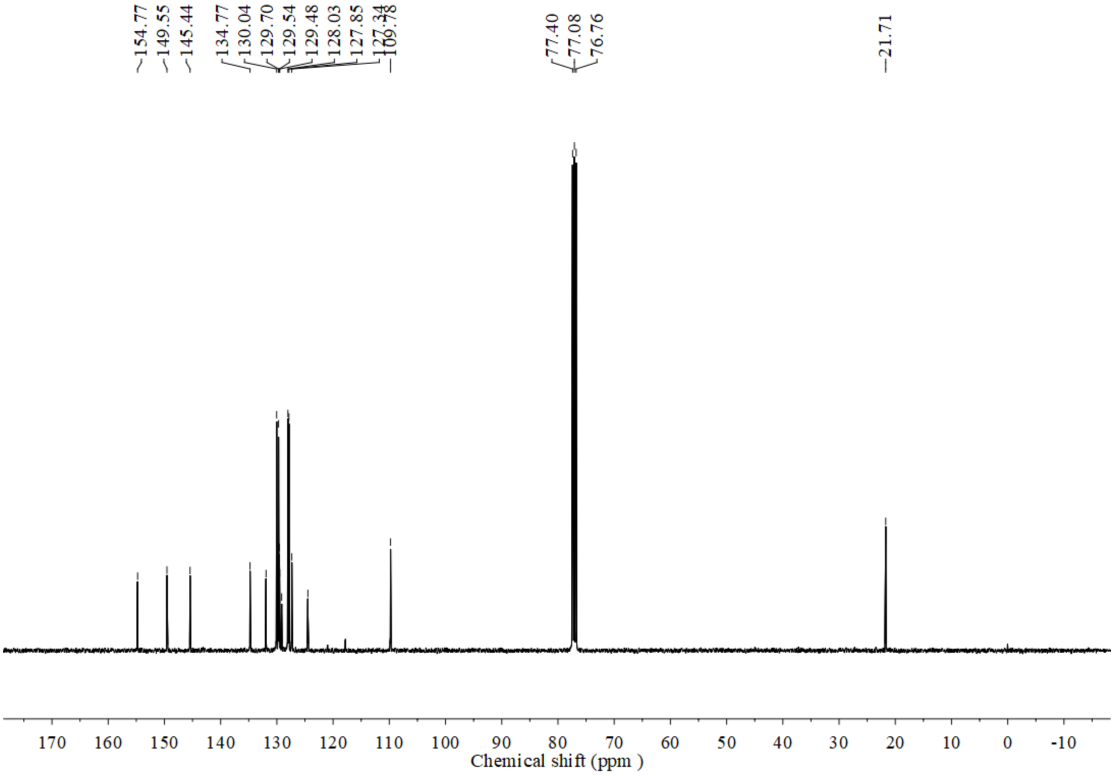

Figure S31 ^{1}H NMR Spectra of Compound of 2p

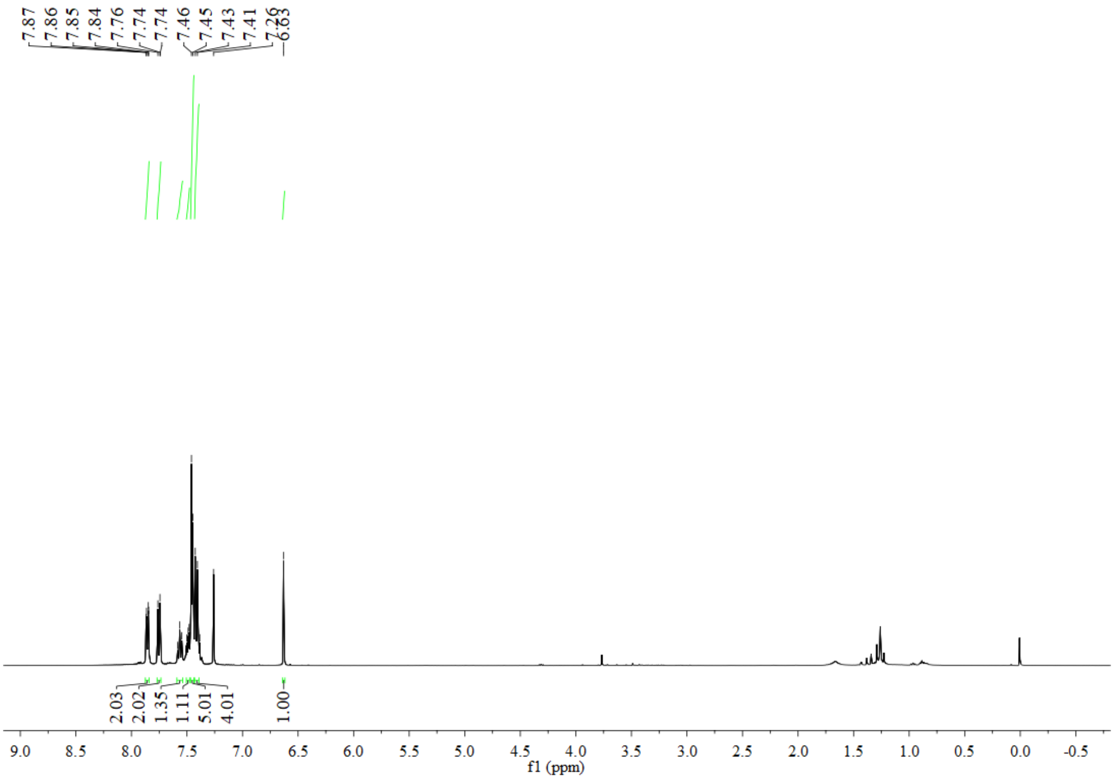

Figure S32 ^{13}C NMR Spectra of Compound of 2p

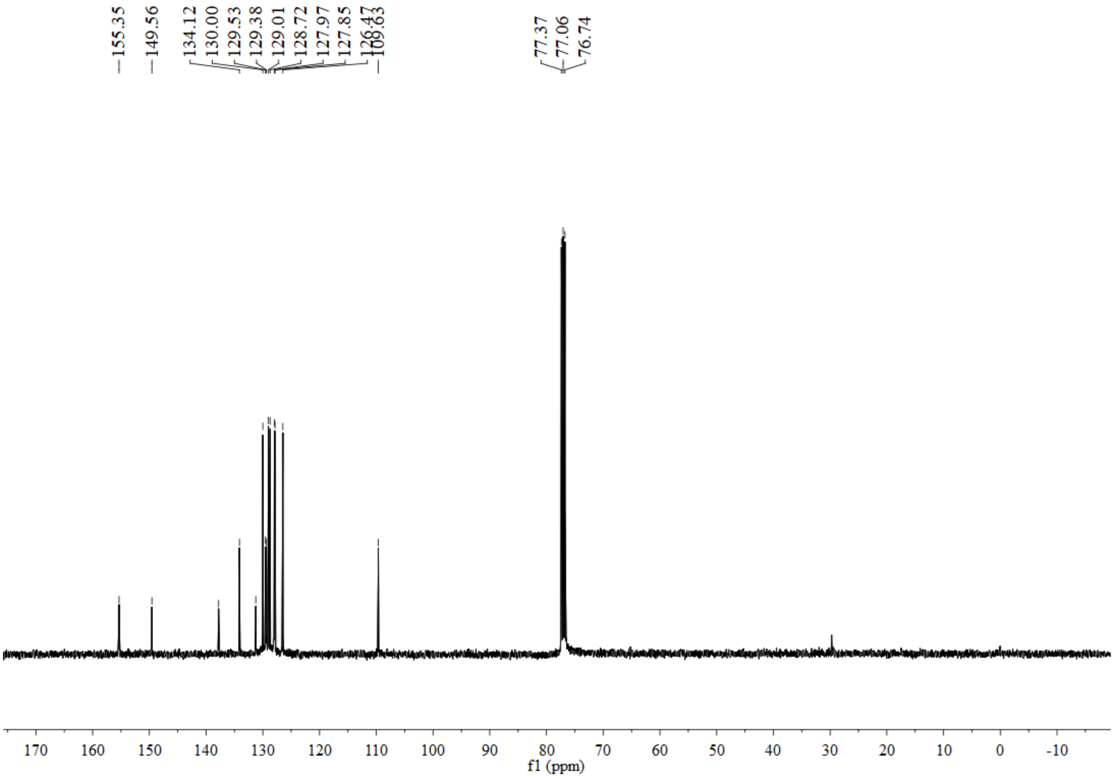

Figure S33 ^{1}H NMR Spectra of Compound of 3a

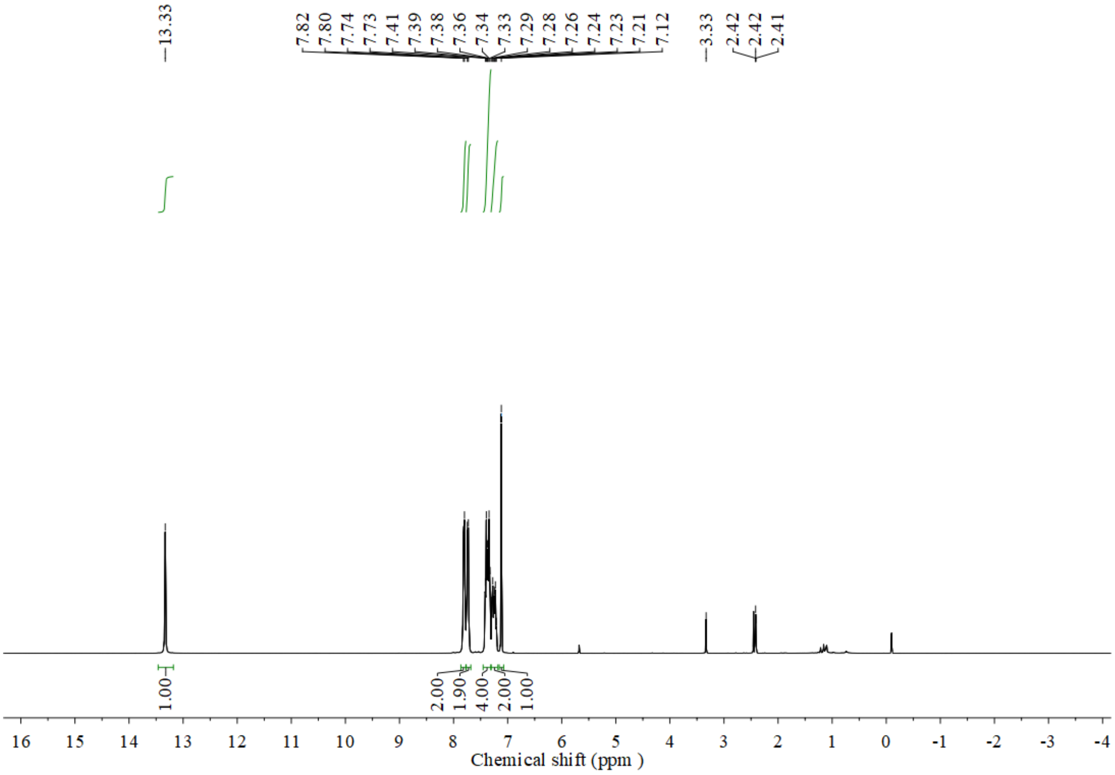

Figure S34 ^{13}C NMR Spectra of Compound of 3a

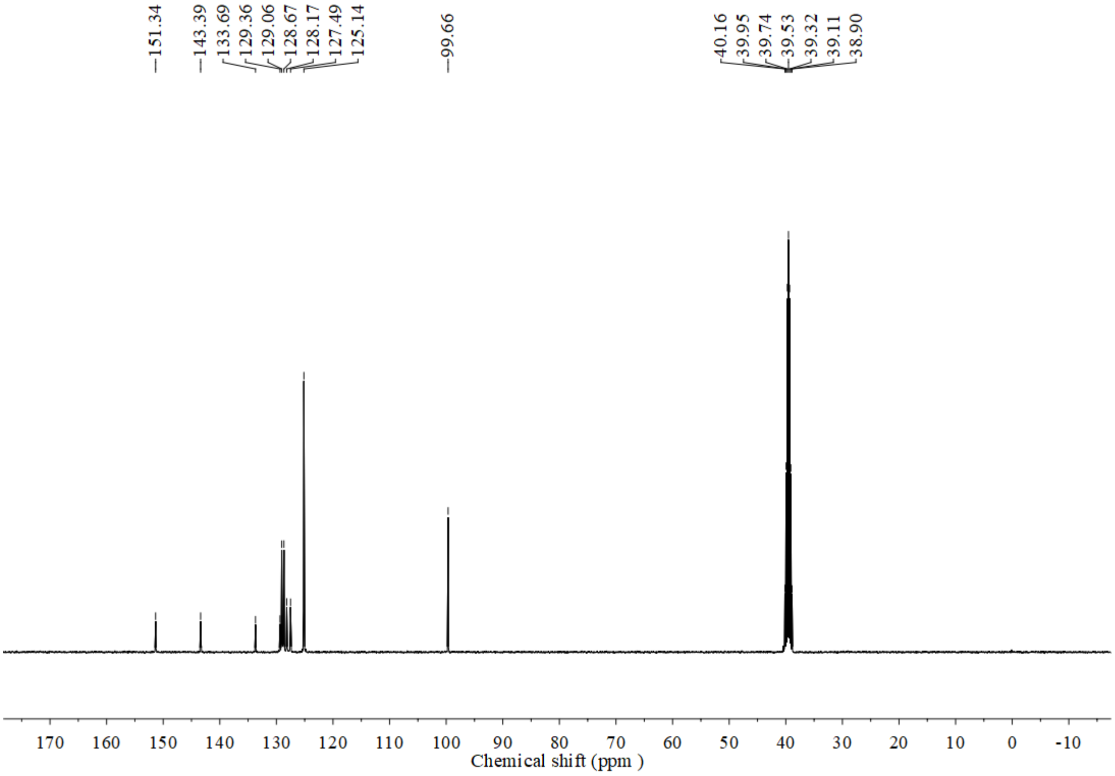

Figure S35 ¹H NMR Spectra of Compound of 3b

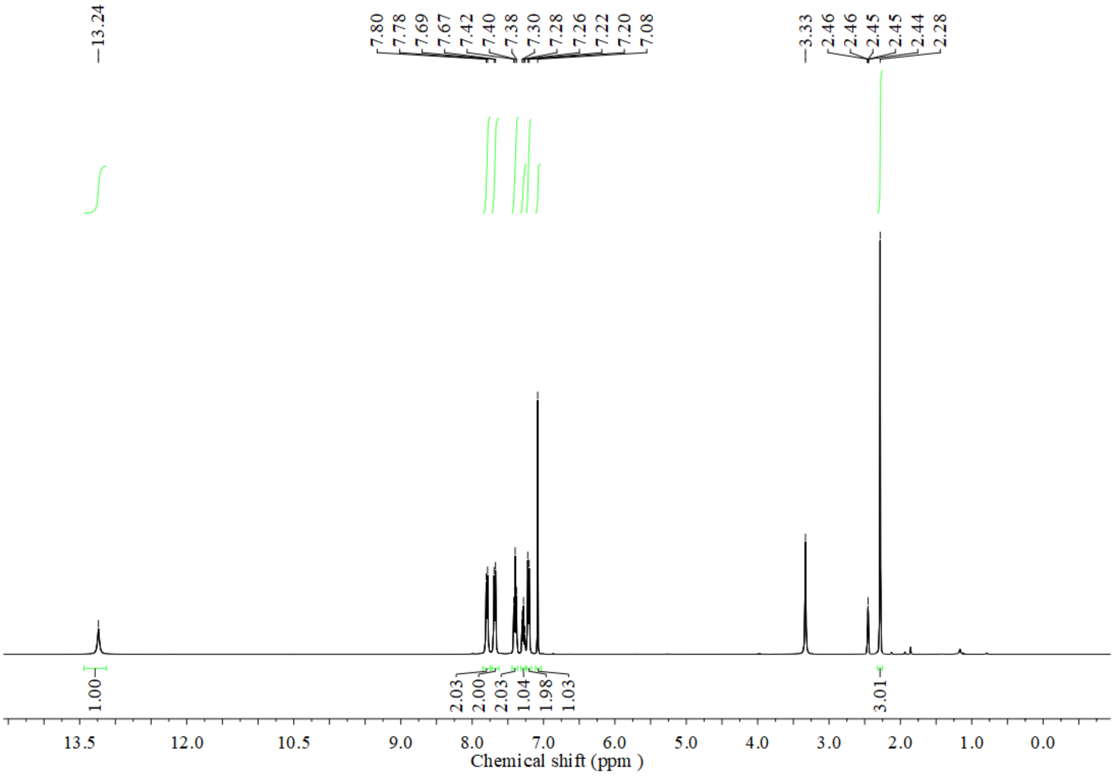

Figure S36 ¹³C NMR Spectra of Compound of 3b

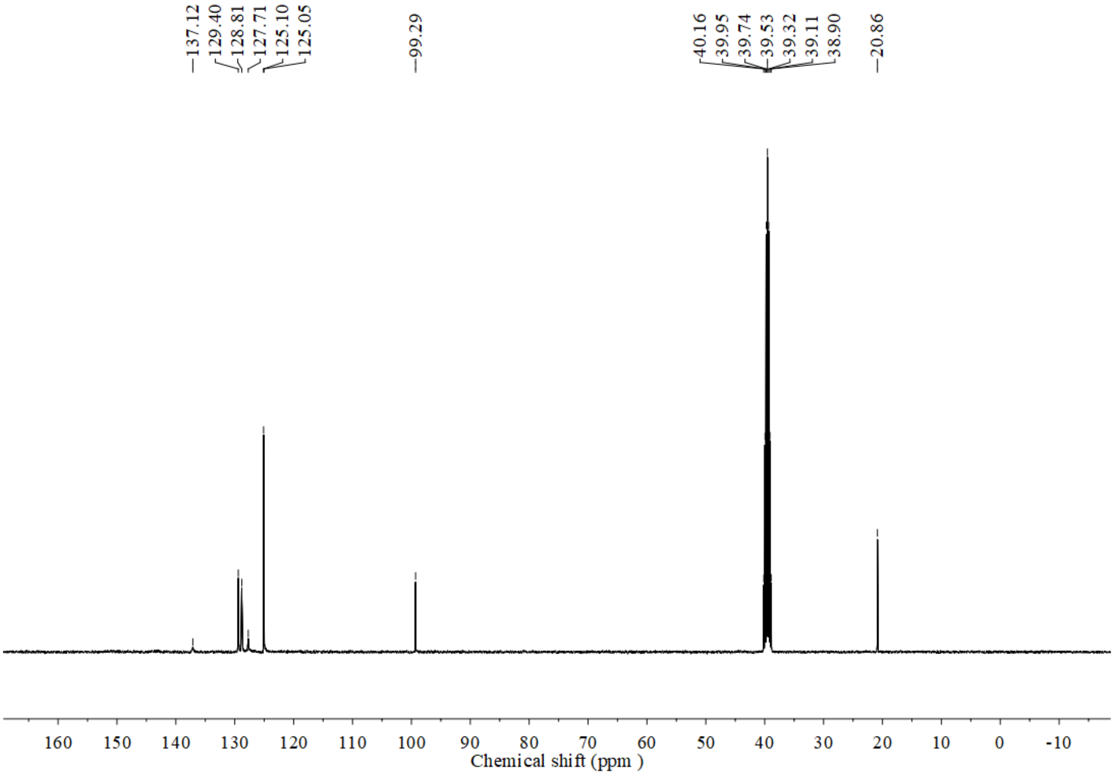

Figure S37 ^{1}H NMR Spectra of Compound of 3c

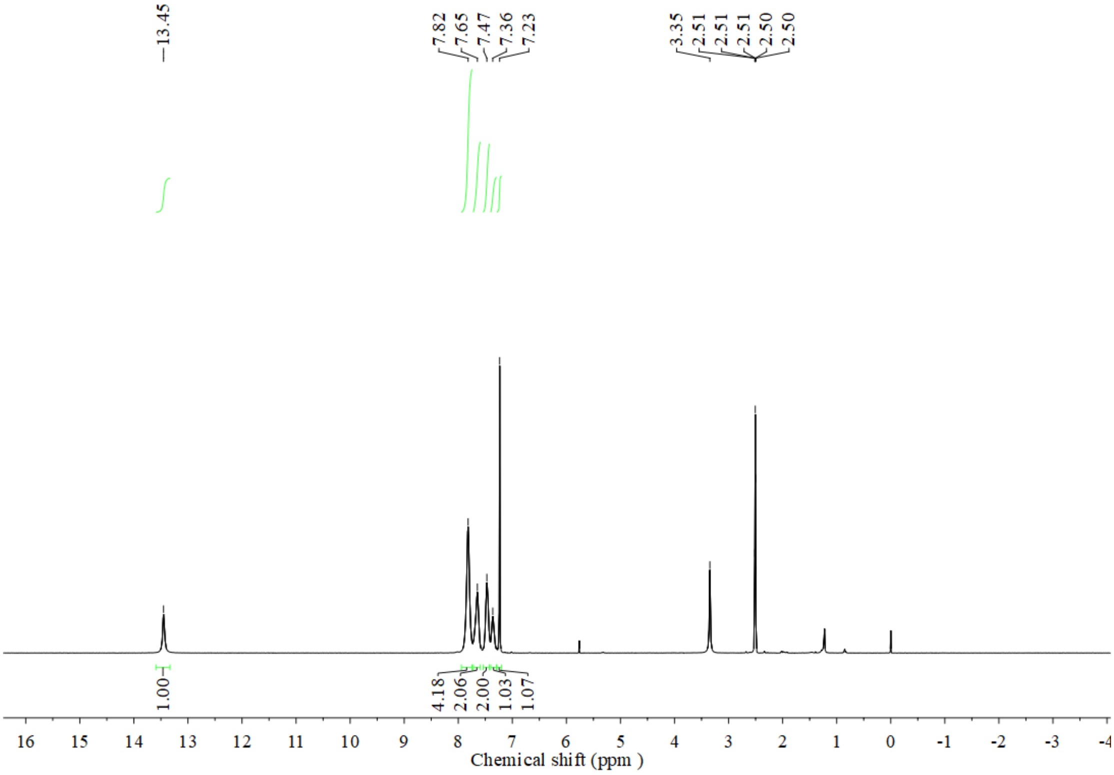

Figure S38 ^{13}C NMR Spectra of Compound of 3c

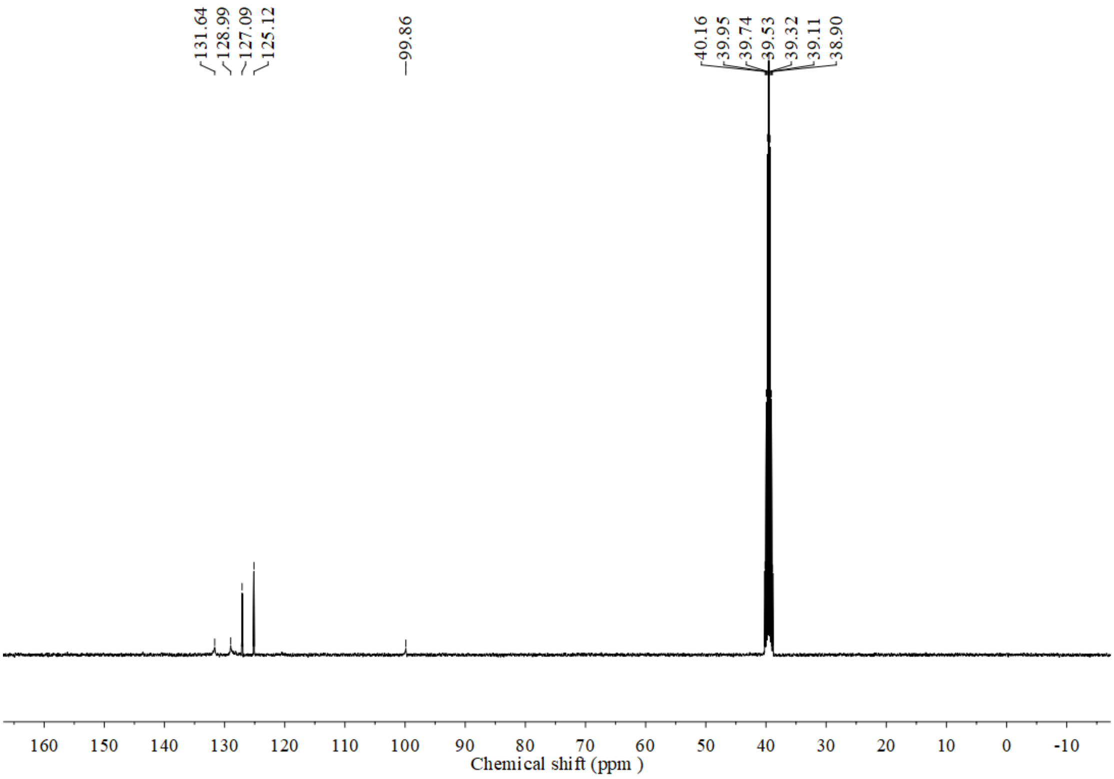

Figure S39 ^{1}H NMR Spectra of Compound of 3d

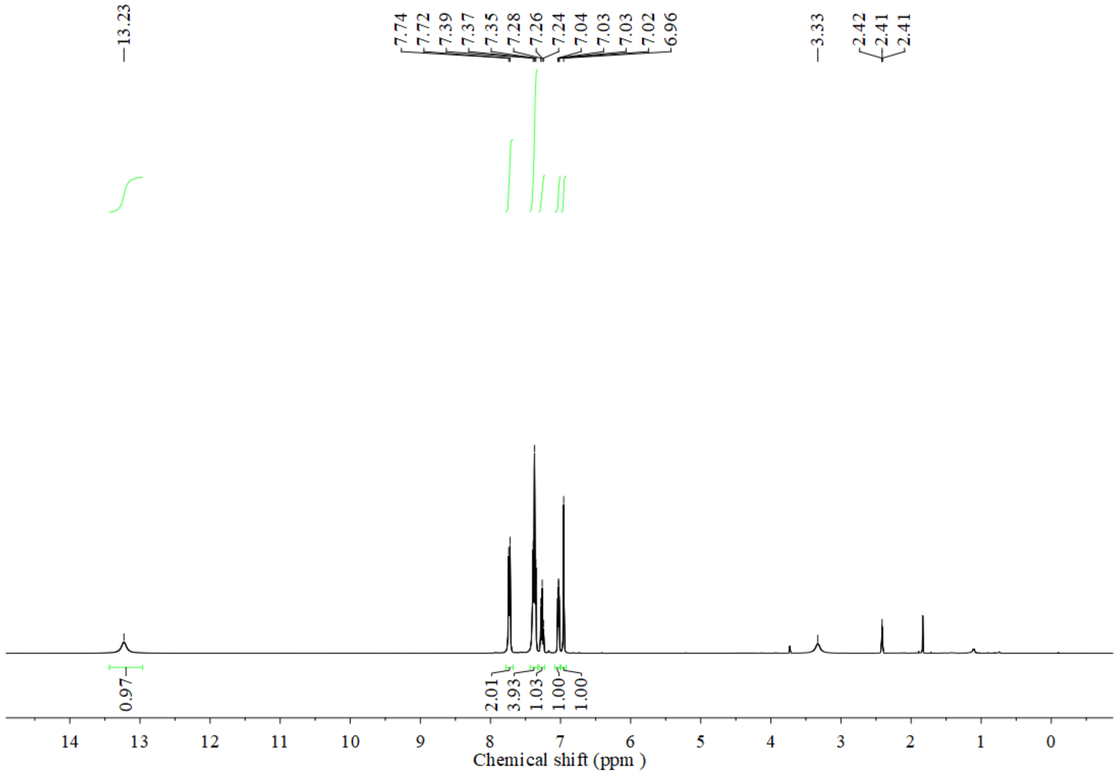

Figure S40 ^{13}C NMR Spectra of Compound of 3d

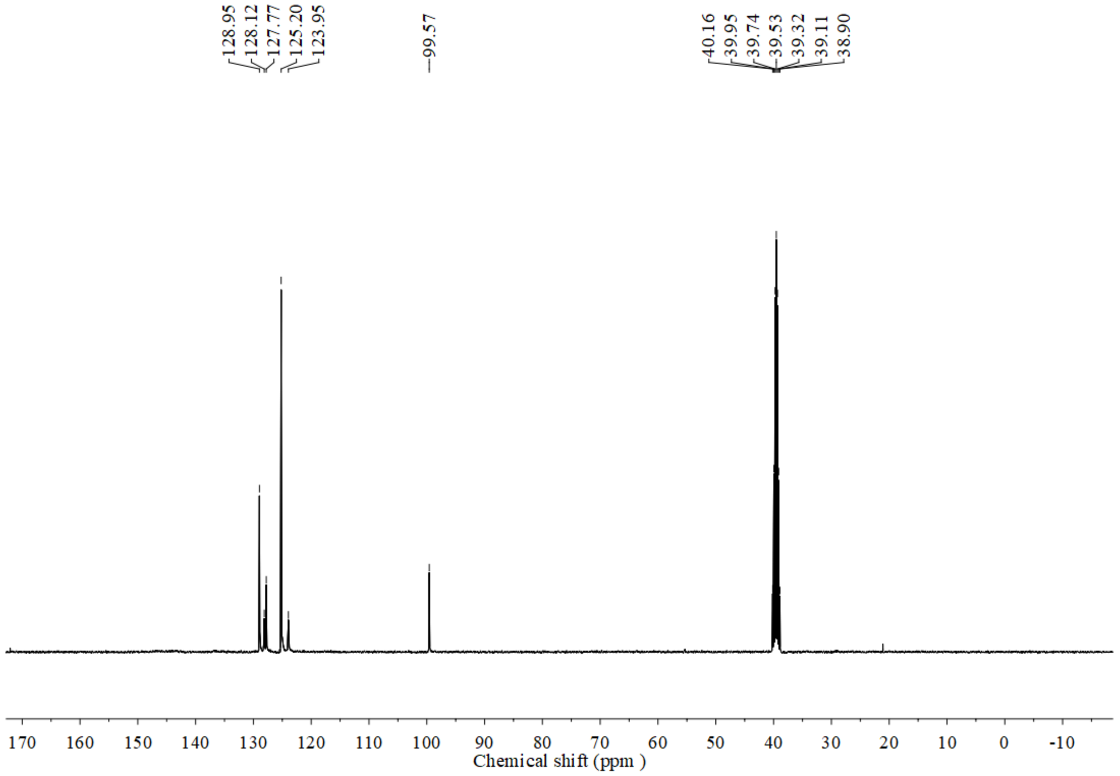

Figure S41 ^{1}H NMR Spectra of Compound of 3e

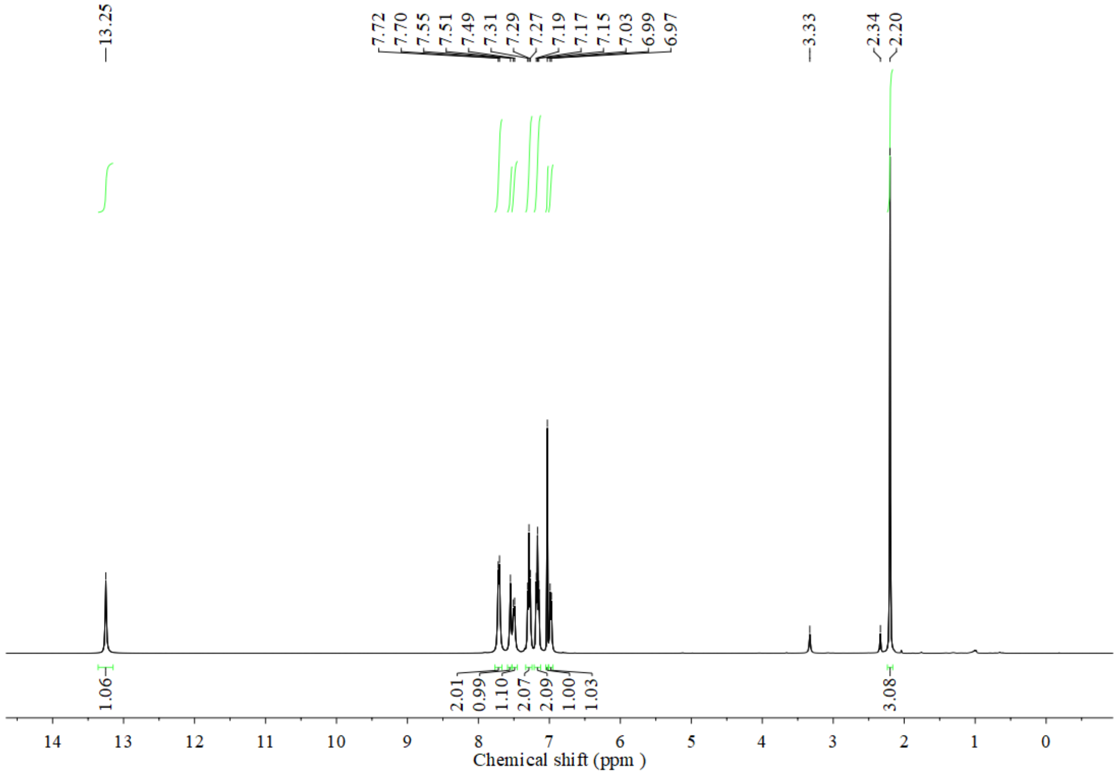

Figure S42 ^{13}C NMR Spectra of Compound of 3e

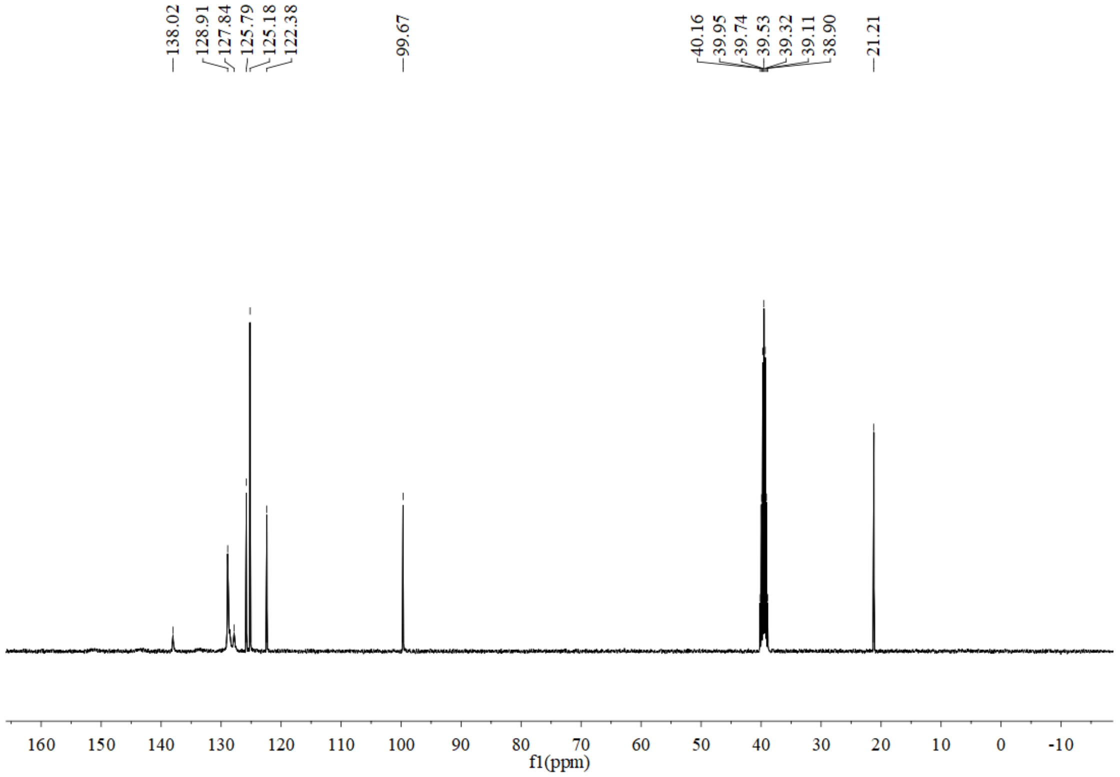

Figure S43 ^{1}H NMR Spectra of Compound of 3f

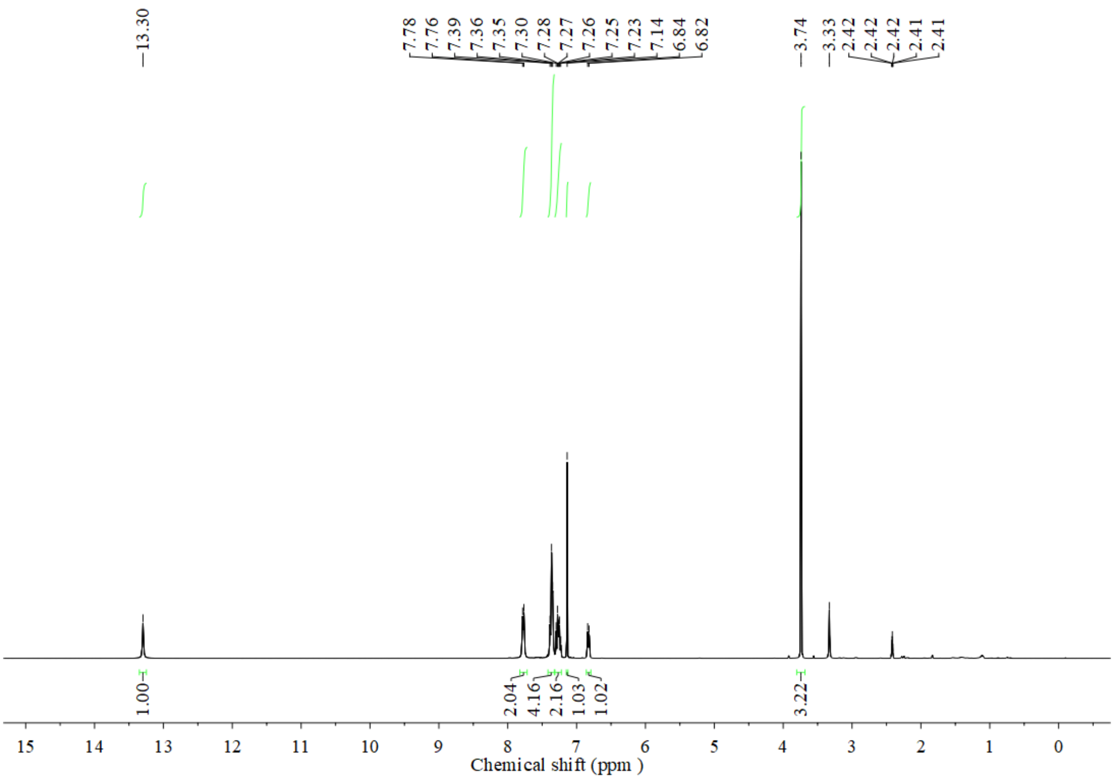

Figure S44 ^{13}C NMR Spectra of Compound of 3f

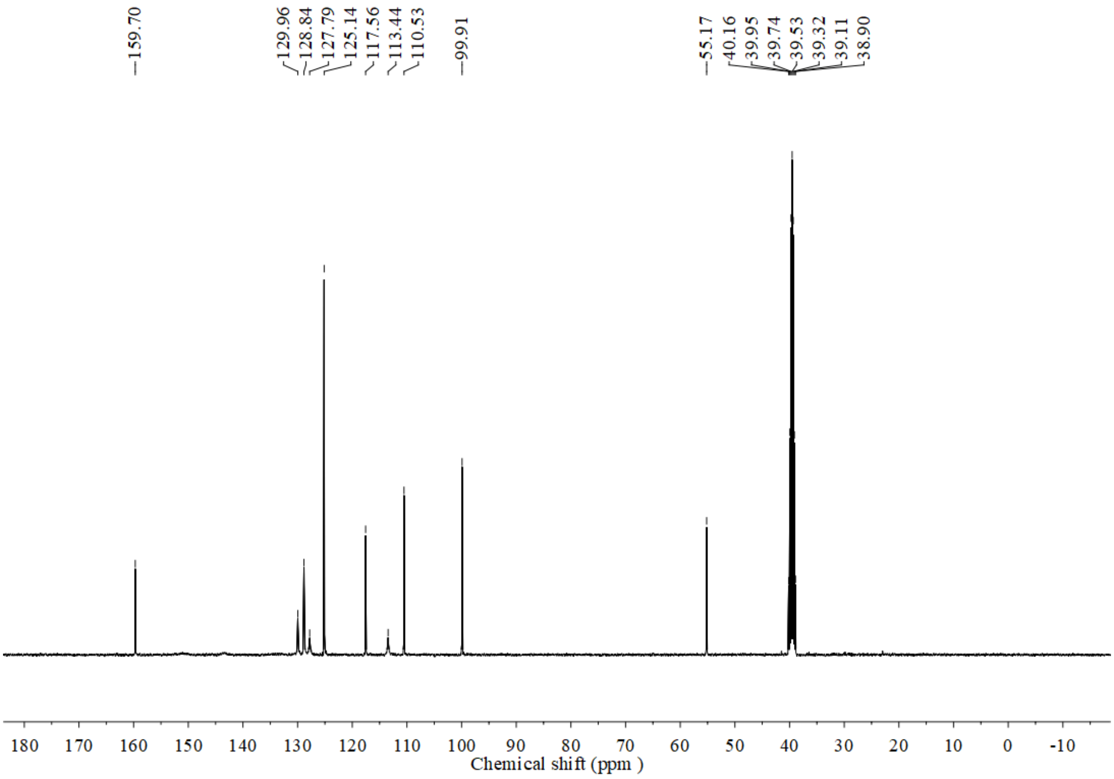

Figure S45 ^{1}H NMR Spectra of Compound of 3g

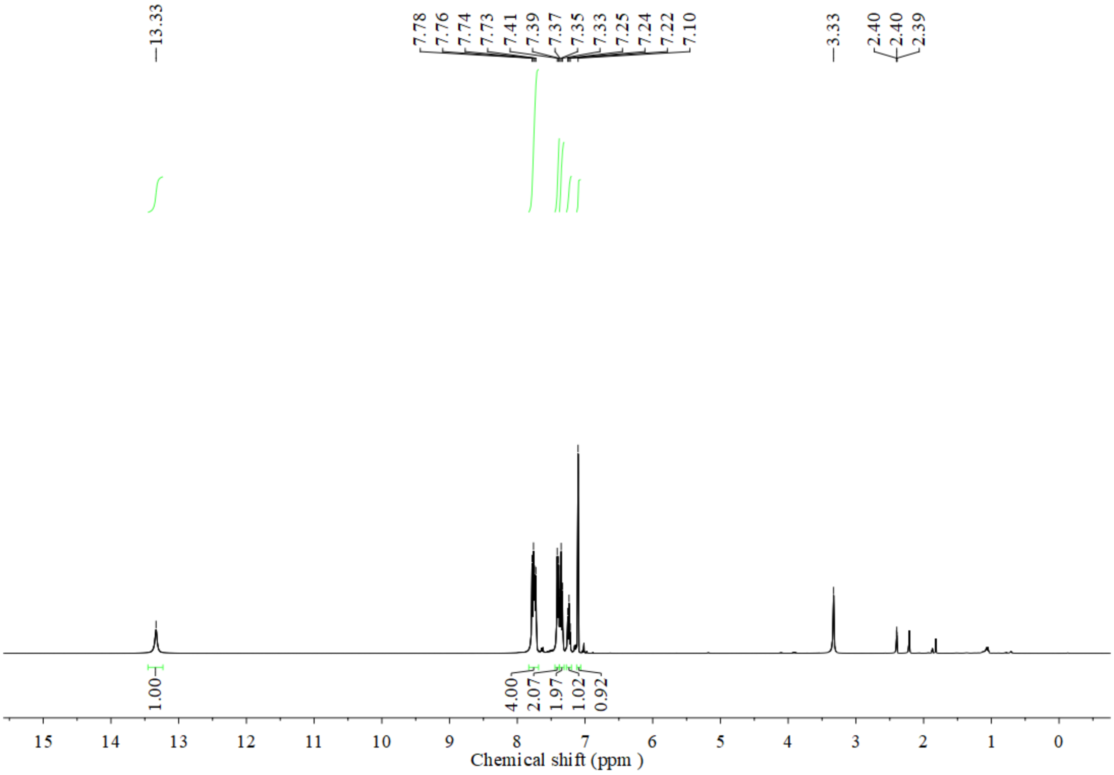

Figure S46 ^{13}C NMR Spectra of Compound of 3g

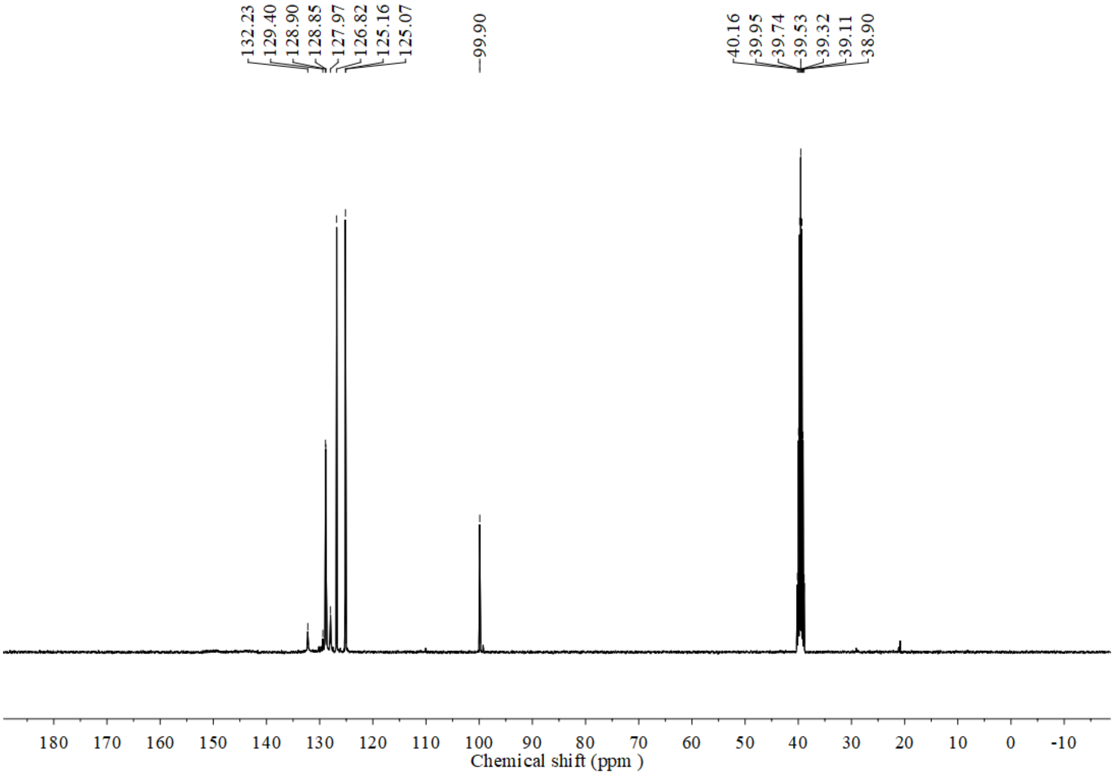

Figure S47 ^{1}H NMR Spectra of Compound of 3h

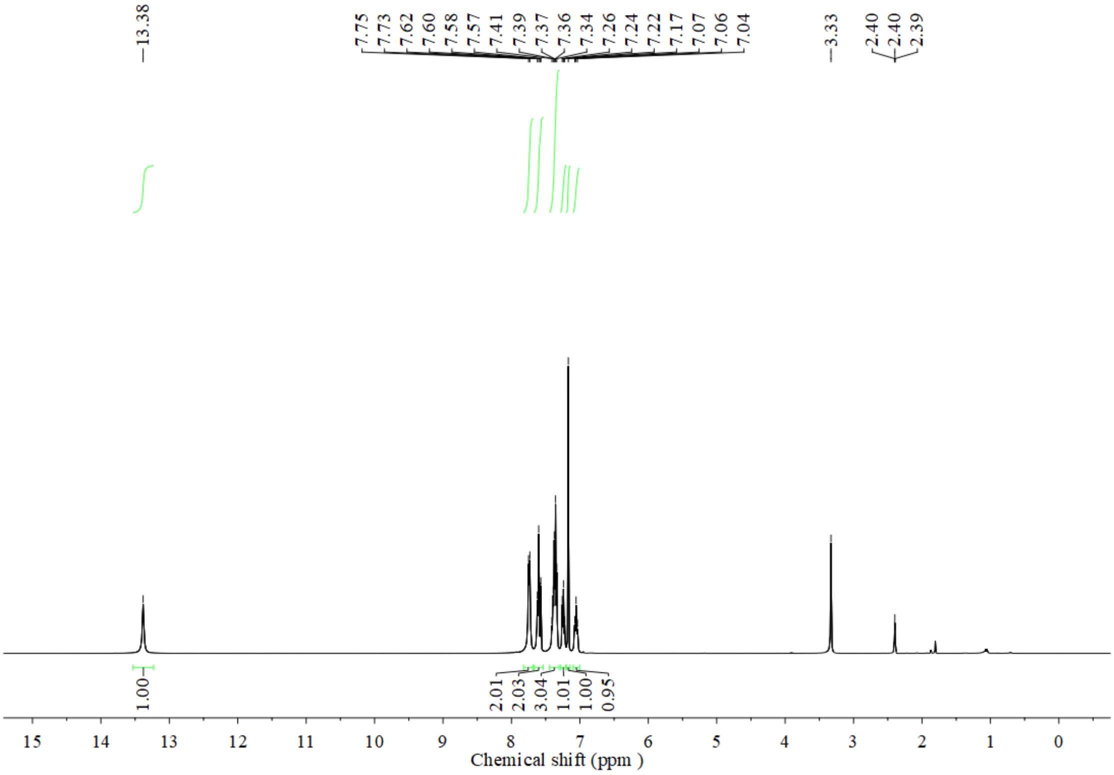

Figure S48 ^{13}C NMR Spectra of Compound of 3h

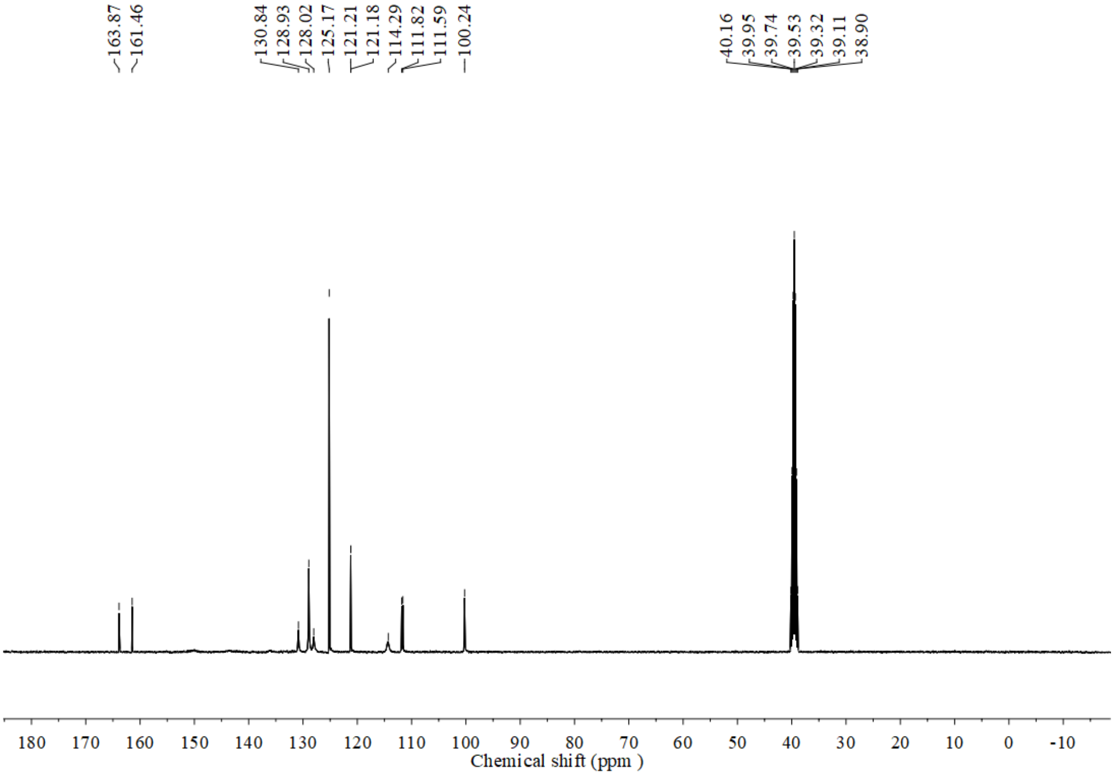

Figure S49 ^{1}H NMR Spectra of Compound of 3i

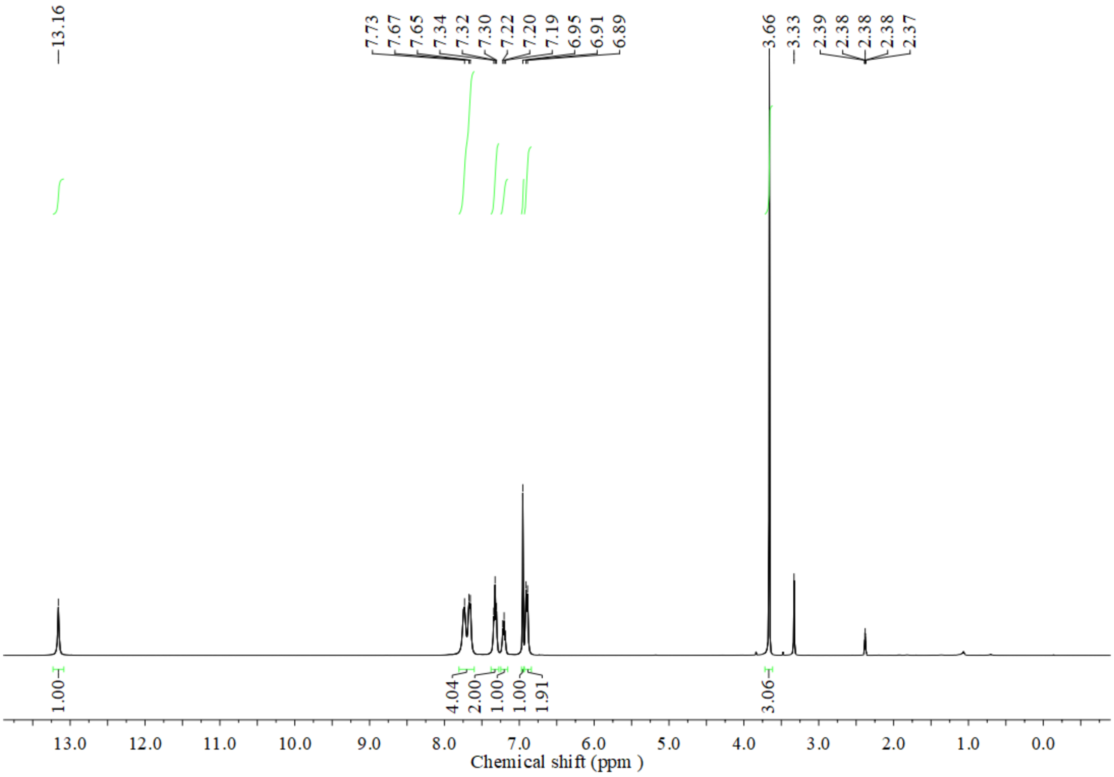

Figure S50 ^{13}C NMR Spectra of Compound of 3i

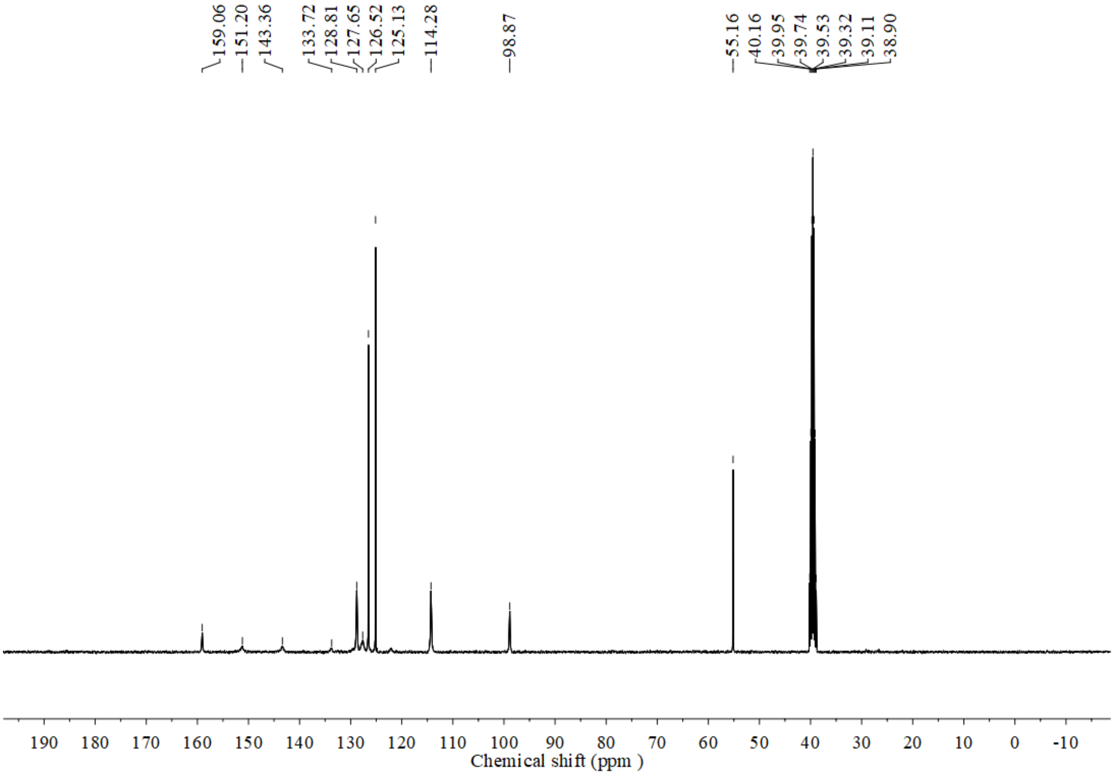

Figure S51 ^{1}H NMR Spectra of Compound of 3j

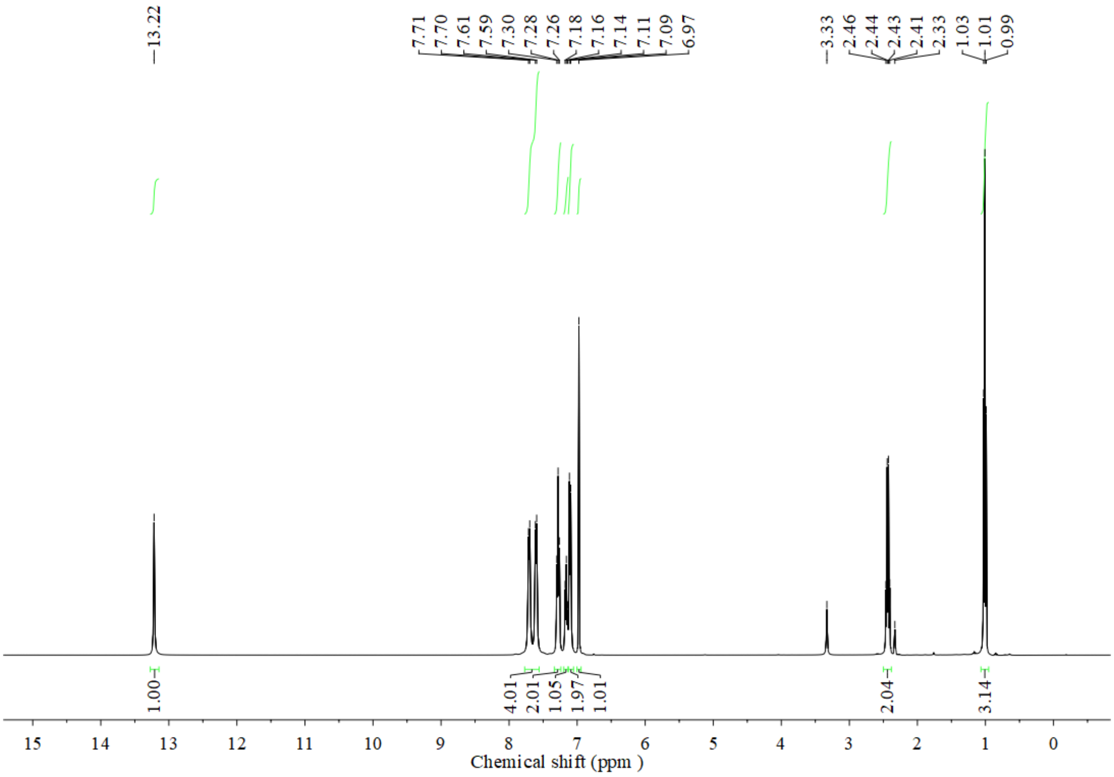

Figure S52 ^{13}C NMR Spectra of Compound of 3j

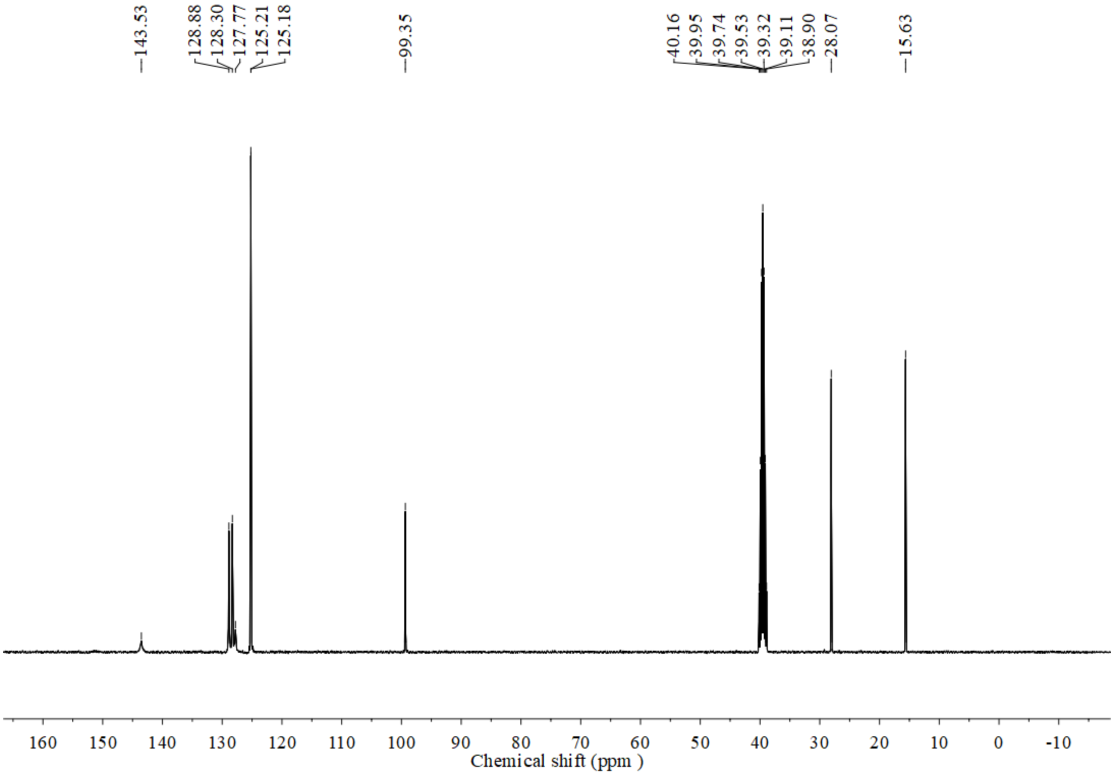

Figure S53 ^{1}H NMR Spectra of Compound of 3k

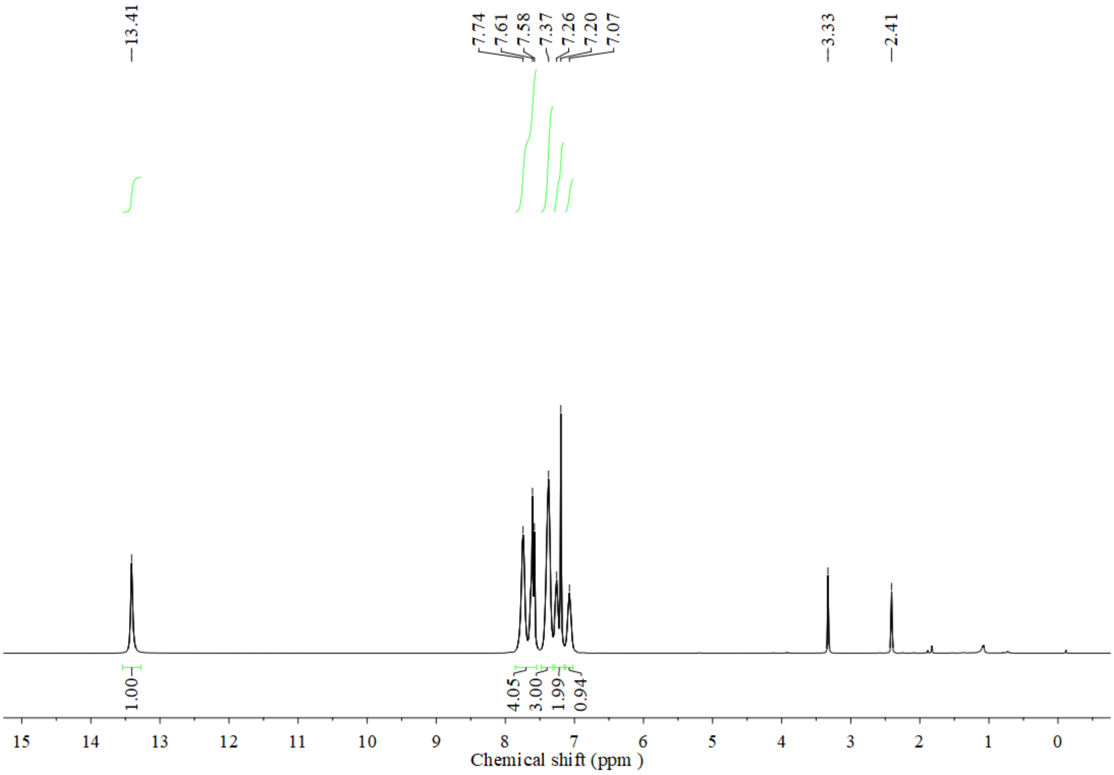

Figure S54 ^{13}C NMR Spectra of Compound of 3k

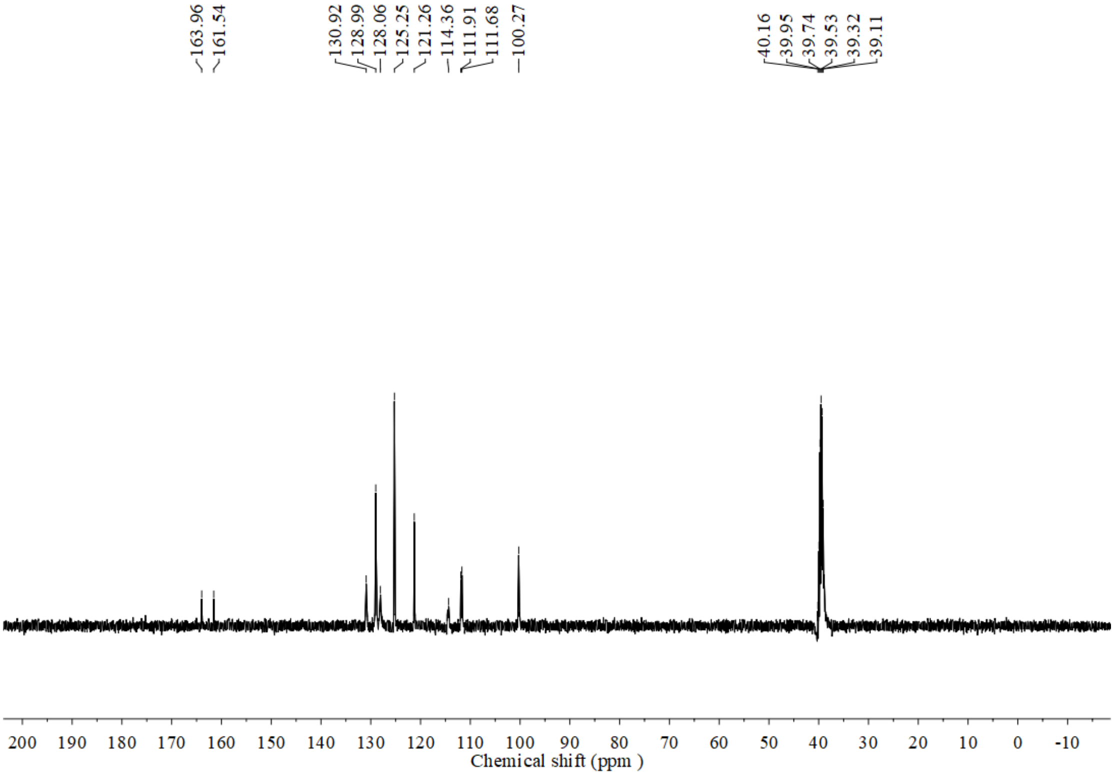

Figure S55 ^{1}H NMR Spectra of Compound of 3l

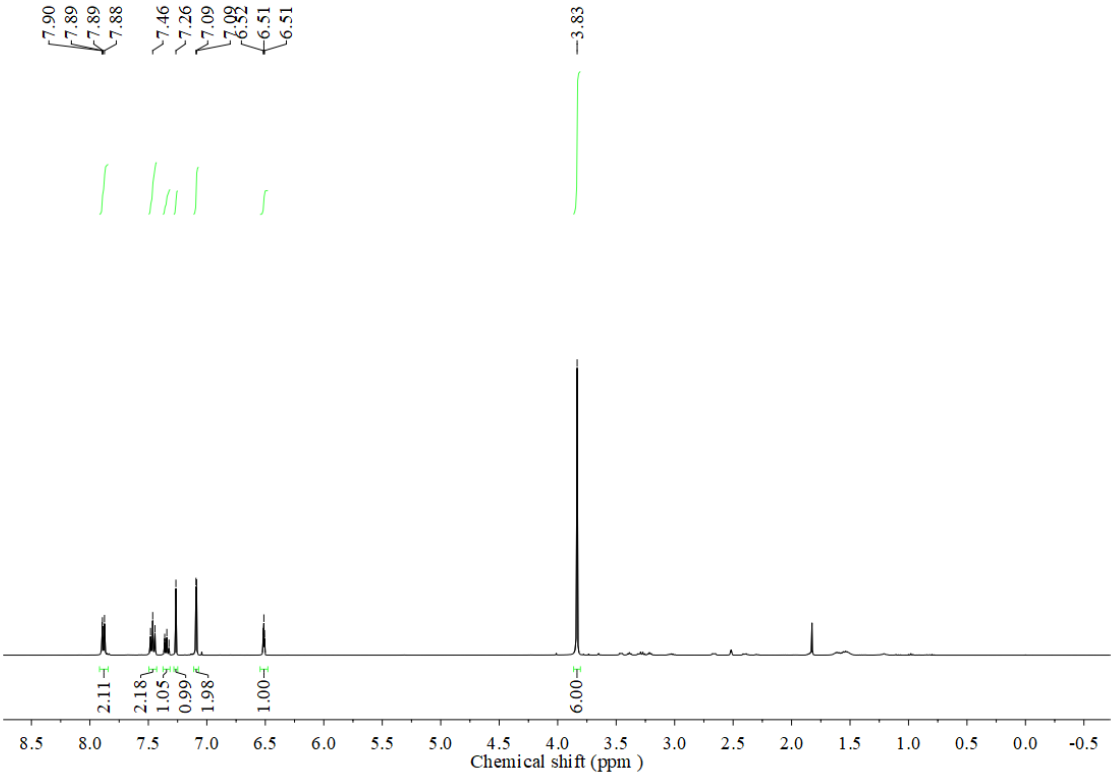

Figure S56 ^{13}C NMR Spectra of Compound of 3l

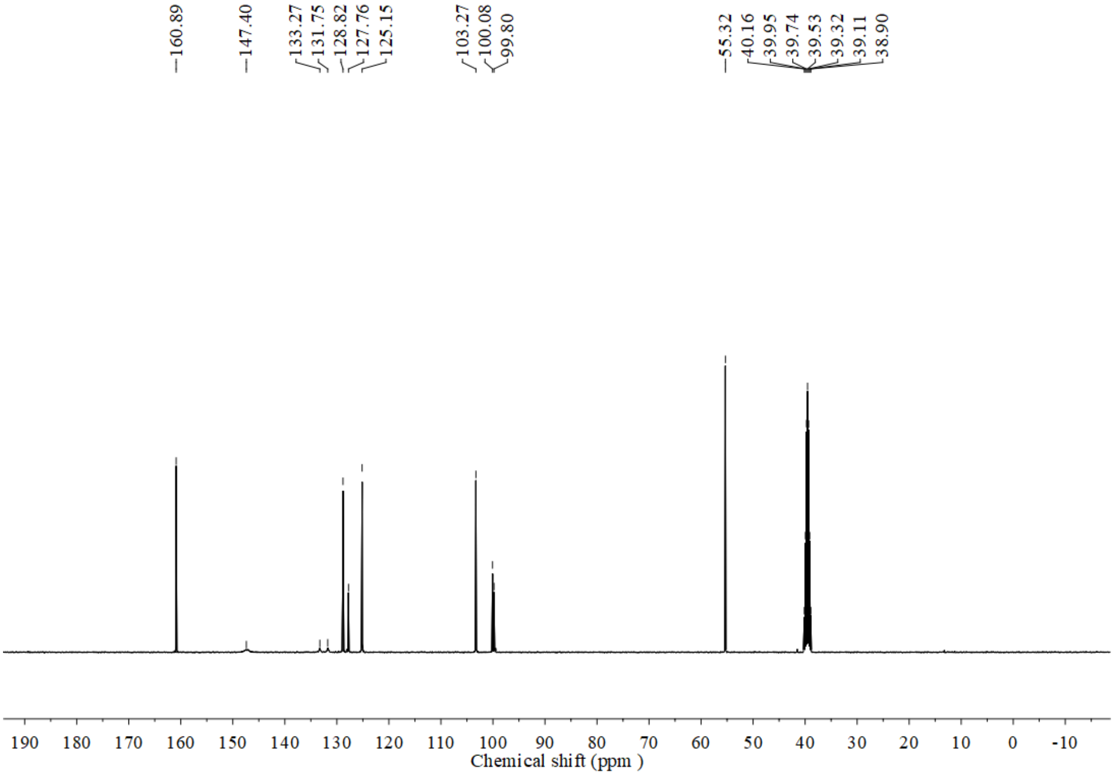

Figure S57 ^{1}H NMR Spectra of Compound of 3m

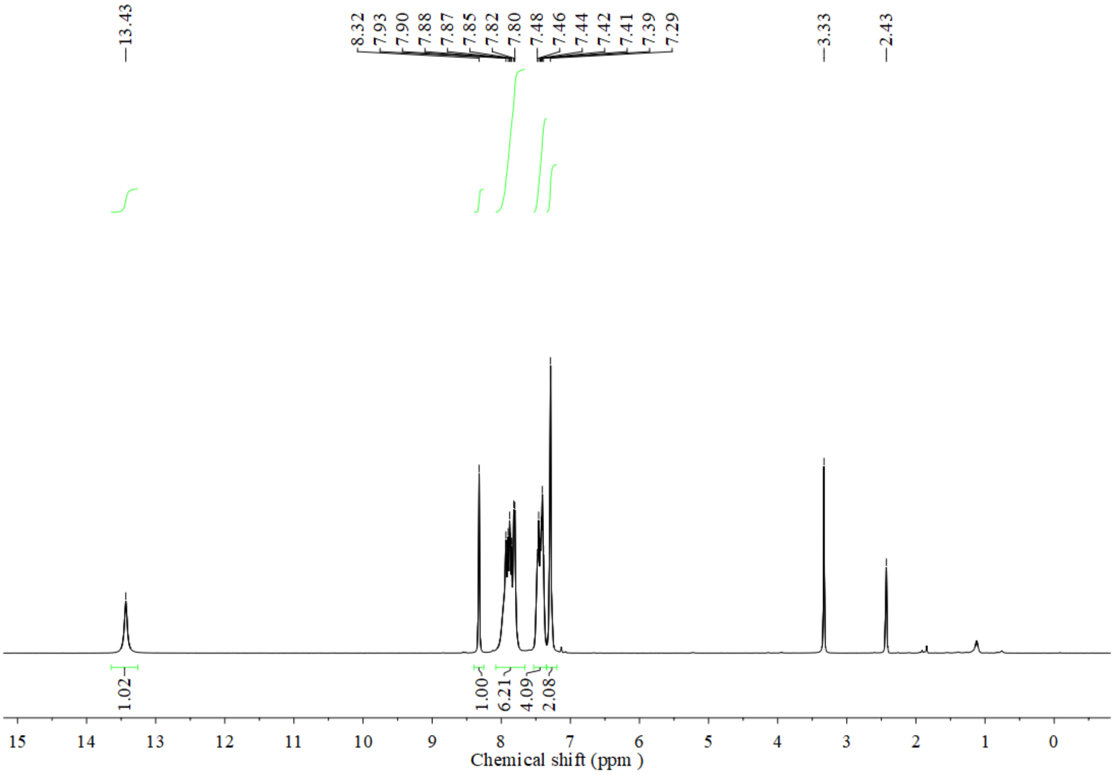

Figure S58 ^{13}C NMR Spectra of Compound of 3m

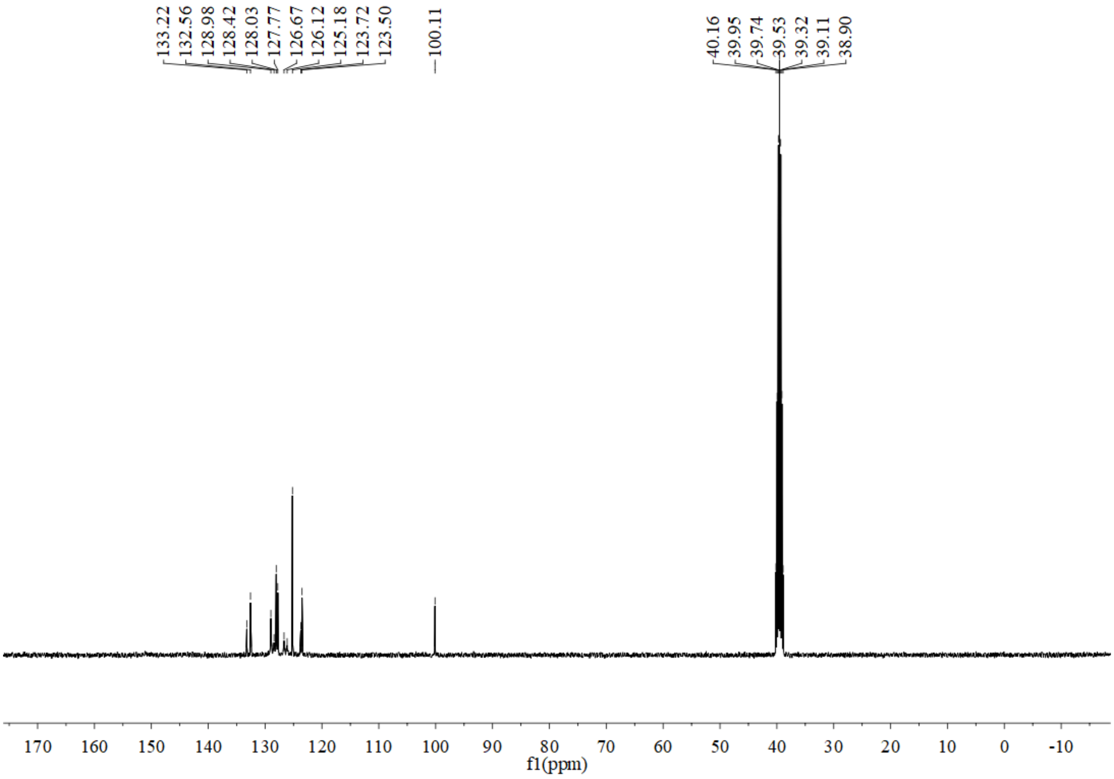

Figure S59 ^{1}H NMR Spectra of Compound of 3n

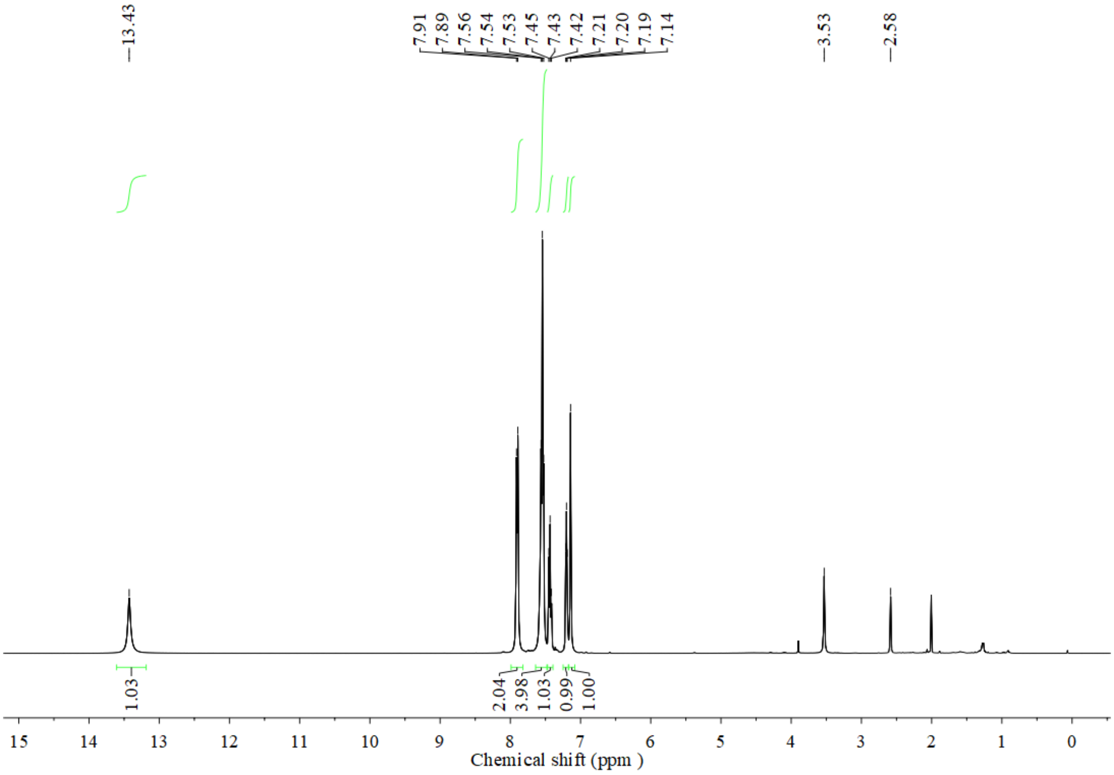

Figure S60 ^{13}C NMR Spectra of Compound of 3n

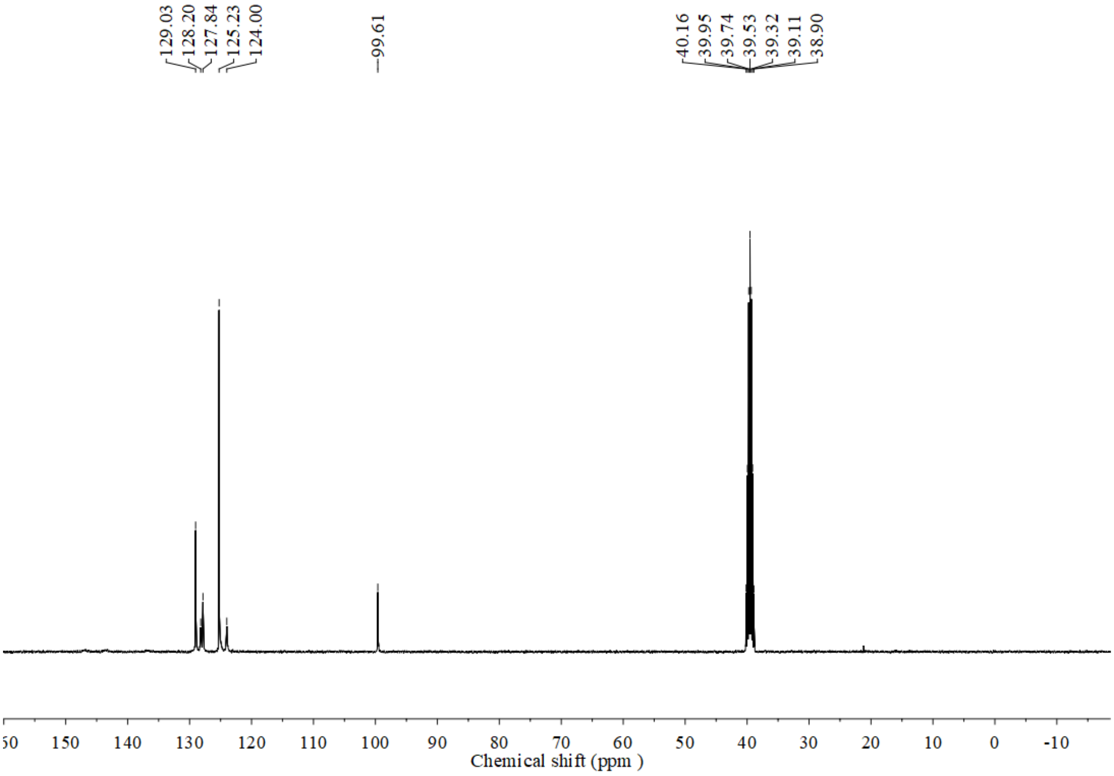

Supplement: Supplementary file 1 [file molecules-29-01706-s001.zip › molecules-2910401-supplementary.pdf]
